# A Mitochondria-Targeting SIRT3 Inhibitor with Activity against Diffuse Large B Cell Lymphoma

**DOI:** 10.1021/acs.jmedchem.4c01053

**Published:** 2024-08-27

**Authors:** Sadhan Jana, Jialin Shang, Jun Young Hong, Michael K. Fenwick, Rishi Puri, Xuan Lu, Ari M. Melnick, Meng Li, Hening Lin

**Affiliations:** † Department of Chemistry and Chemical Biology, Cornell University, Ithaca, New York 14853, United States; ‡ College of Veterinary Medicine, Cornell University, Ithaca, New York 14853, United States; § Department of Medicine, Division of Hematology & Medical Oncology, Weill Cornell Medicine, New York, New York 10065, United States; ∥ Howard Hughes Medical Institute; Department of Chemistry and Chemical Biology; Department of Molecular Biology and Genetics Cornell University Ithaca New York 14853 United States

## Abstract

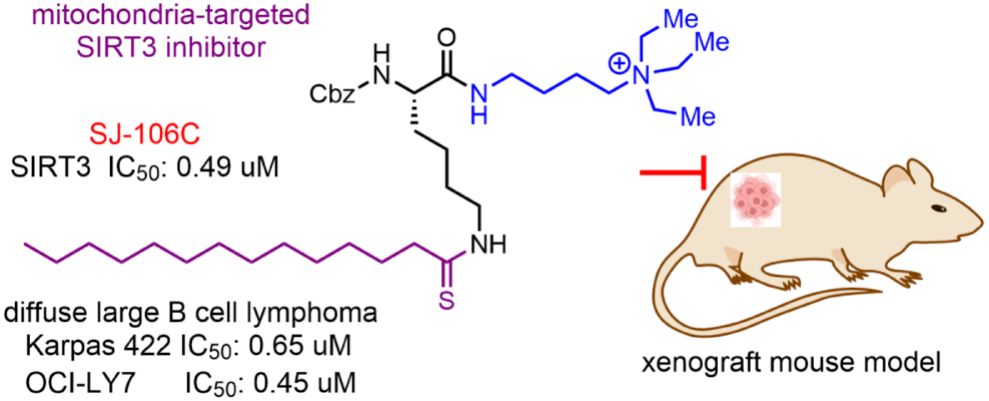

Diffuse large B-cell lymphomas (DLBCLs) are heterogeneous cancers that still require better and less toxic treatments. SIRT3, a member of the sirtuin family of NAD^+^-dependent protein deacylase, is critical for DLBCL growth and survival. A mitochondria-targeted SIRT3 small-molecule inhibitor, YC8-02, exhibits promising activity against DLBCL. However, YC8-02 has several limitations including poor solubility. Here, we report our medicinal chemistry efforts that led to an improved mitochondria-targeted SIRT3 inhibitor, SJ-106C, achieved by using a triethylammonium group, which helps to increase both solubility and SIRT3 inhibition potency. SJ-106C, while still inhibiting SIRT1 and SIRT2, is enriched in the mitochondria to help with SIRT3 inhibition. It is more active against DLBCL than other solid tumor cells and effectively inhibits DLBCL xenograft tumor growth. The findings provide useful insights for the development of SIRT3 inhibitors and mitochondrial targeting agents and further support the notion that SIRT3 is a promising druggable target for DLBCL.

## Introduction

Diffuse large B-cell lymphomas (DLBCLs) represent the most common and aggressive subtype of non-Hodgkin’s lymphoma (NHL) which is the most common hematological malignancy. DLBCLs are highly proliferative and genetically heterogeneous and rank among the most extensively mutated cancers due to their origin from B-cells undergoing somatic hypermutation in germinal center (GC) reactions. Next-generation sequencing studies on DLBCL tumors have revealed intra- and inter-tumor heterogeneity at genetic and epigenetic levels.^1,2^ Patients have highly variable mutation profiles, complicating efforts to develop precision therapies via targeting specific oncogenic mutations. In spite of its many side effects, R-CHOP chemoimmunotherapy is still the standard of care for DLBCL patients. Up to 40% of patients remain incurable despite receiving second-line therapies. It is thus necessary to identify therapeutic vulnerabilities relevant to a broad cross-section of patients regardless of their mutational profiles.^3−5^

We recently identified the mitochondrial sirtuin, SIRT3, a master regulator of mitochondrial stress metabolism, as a critical driver of DLBCL growth and survival through its role in promoting glutaminolysis.^6^ Of note, highly active mitochondrial glutaminolysis and oxidative phosphorylation are associated with DLBCL resistance to current therapies including R-CHOP, venetoclax, and ibrutinib.^5,7,8^ Hence, a potent SIRT3 inhibitor has the potential to significantly improve the efficacy of DLBCL treatments.

Sirtuins constitute a conserved family of nicotinamide adenine dinucleotide (NAD^+^)-dependent lysine deacylases with seven members known as SIRT1–7 in mammals. Sirtuins differ in their subcellular localization and substrate specificities. They play key roles in regulating metabolism, stress responses, and aging processes, and are considered therapeutic targets for various diseases.^9^ The mitochondrial sirtuins SIRT3, SIRT4, and SIRT5 have emerged as potential therapeutic targets for diseases such as metabolic disorders and cancers.^10^ Among the seven sirtuins, only SIRT3 was broadly required for the growth and survival of DLBCL cells.^6^

SIRT3 deacetylates a broad variety of mitochondrial proteins involved in cellular metabolism. This contributes to diverse functions within mitochondrial physiology, including energy metabolism, reactive oxygen species (ROS) detoxification, autophagy, and apoptosis.^6,11,12^ Among sirtuins, SIRT1, 2, and 3 share similar enzymatic activities, which is challenging for the design of SIRT3 specific inhibitors. In our prior work, we developed the first mitochondrial localized sirtuin inhibitor, YC8-02. YC8-02 concentrated in mitochondria due to its inclusion of a triphenylphosphonium (TPP) mitochondrial-targeting group and had potent activity against DLBCLs in vitro and in vivo,^6^ but little effect against cell types that were not dependent on SIRT3. However, the TPP group has poor solubility and limits further preclinical development. In this study, we developed a second-generation SIRT3 inhibitor, SJ-106C, with a triethylammonium mitochondrial-targeting moiety. In vitro and in vivo characterization of SJ-106C showed that the triethylammonium functional group efficiently targets SJ-106C to mitochondria and suppresses DLBCL tumor growth, further supporting the therapeutic potential of SIRT3 inhibition in DLBCL and providing critical new insights for developing improved SIRT3 inhibitors in the future.

## Results

### Development of a New SIRT3 Inhibitor SJ-106C

**Figure 1:**
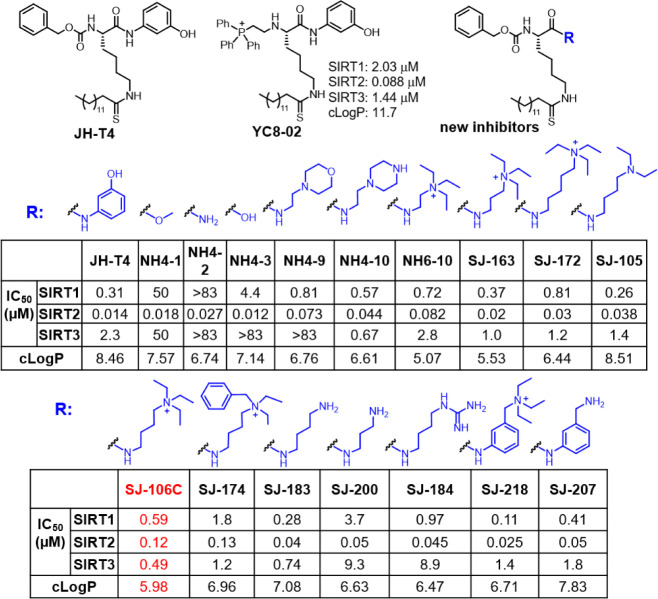
Structures of various SIRT3 inhibitors developed and in vitro IC_50_ values against SIRT1/2/3. Enzymatic activity was detected by using an HPLC assay.

DLBCLs are broadly addicted to SIRT3, and SIRT3 deletion or inhibition efficiently kills DLBCL cells in vitro and in vivo.^6^ The previously reported SIRT3 inhibitor YC8-02 (Figure 1) was the first reported highly active SIRT3 inhibitor. It uses a TPP group to increase mitochondria and SIRT3 targeting (SIRT3 is localized in the mitochondria).^6^ TPP is very hydrophobic with a poor aqueous solubility. Hydrophobicity also limits bioavailability, as serum albumin may bind and sequester it. Furthermore, the IC_50_ value of YC8-02 for SIRT3 was 1.44 μM, which could be improved for greater therapeutic efficacy. Therefore, our goal is to develop new SIRT3 inhibitors with different mitochondria-targeting moieties to further improve the solubility and potency.

YC8-02 is a mechanism-based inhibitor containing a thiomyristoyl-lysine moiety (Figure 1). The thiomyristoyl group is important for SIRT3 inhibition, and thus, we decided to keep this constant. While both the N- and C-termini of the lysine could be modified to obtain compounds with improved pharmacological properties and SIRT3 potency/selectivity, in this study, we focused on changing the C-terminus of lysine (including attachment of potential mitochondria-targeting moieties to the C-terminus) while keeping the N-terminus as benzyl carbamate (Cbz). Later, structural data revealed that the choice of fixing the N-terminus as Cbz is beneficial. Given the desire to decrease hydrophobicity and increase solubility, we tried to use less hydrophobic mitochondria-targeting moieties such as triethylammonium.^13−15^ We chose a few positively charged quaternary ammoniums (or tertiary amines that can be protonated and positively charged) to increase the aqueous solubility and act as a mitochondrial targeting moiety (Figure 1). Importantly, we found that many of these groups also increased the SIRT3 inhibition potency. Among these improved compounds, SJ-106C is the most potent, with an SIRT3 IC_50_ value of 0.49 μM, which is a nearly 3-fold enhancement over that of YC8-02.

SJ-106C has a calculated *C* log *P* value of 5.9, suggesting a more favorable solubility profile compared with YC8-02, which has a *C* log *P* value of 11.7. To compare their solubilities, both compounds were dissolved in the vehicle containing 10% DMSO and 90% PBS, and diluted to a final concentration of 50 mg/mL for dosing at 100 mg/kg in mice. As expected, SJ-106C remained soluble under these conditions, while precipitation immediately formed for YC8-02 (Figure S1). This finding confirmed the improved solubility of SJ-106C. We attempted to further increase the solubility of SJ-106C by shortening the thioacyl chain (Figure S2). Unfortunately, the modification led to a dramatic decrease in SIRT3 inhibition potency and was not further pursued.

### X-Ray Crystal Structure of SIRT3 in the Complex with NH6-10 Explains the Inhibition Potency of Many SIRT3 Inhibitors

All the compounds shown in Figure 1 can inhibit SIRT2 very well (with IC_50_ values less than 1 μM, but only a few can inhibit SIRT3 with IC_50_ values of 1 μM or lower. These observations show that mechanism-based thiomyristoyl-lysine inhibitors work the best for SIRT2. For SIRT2 inhibition, the capping groups on the N or C-termini do not matter much as long as the thiomyristoyl-lysine is present. In contrast, for SIRT3, the structures on the *N*- and C-termini of the thiomyristoyl-lysine are critical for inhibiting SIRT3. For example, NH4-1 to NH4-9 do not really inhibit SIRT3 even though they inhibit SIRT2 very well. Interestingly, those that can inhibit SIRT3 with IC_50_ values of around 1 μM all have amine or quaternary ammonium groups at the C-termini of the thiomyristoyl-lysine compounds.

**Figure 2:**
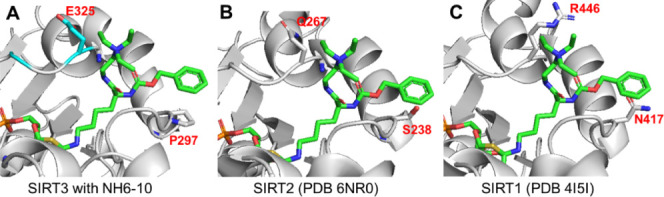
X-ray crystal structure of SIRT3 in the complex with NH6-10 explains the structure–activity trends observed for the inhibitors shown in Figure 1. (A) Structure of SIRT3 in a complex with NH6-10. The covalent intermediate formed between NH6-10 and NAD^+^ is shown in stick representation. The two key residues, P297 and E325, are also shown. (B,C) The structures of SIRT2 (PDB 6NR0) (B) and SIRT1 (PDB 4I5I) (C) were superimposed to that of SIRT3 in (A). The covalent intermediate formed between NH6-10 and NAD^+^ on SIRT3 is shown in stick representation to highlight the active sites. The residues corresponding to P297 and E325 of SIRT3 are also shown (S238 and Q267 in SIRT2, and N417 and R446 in SIRT1).

To explain the SIRT3 inhibitor potency trend, we tried to obtain the SIRT3 X-ray crystal structure in complex with newly developed SIRT3 inhibitors. Among many of the inhibitors we tried, we were able to obtain the X-ray crystal structures of SIRT3 in complex with NH6-10 (Figure 2), which has high aqueous solubility and facilitated the crystallization process. The complex appears to crystallize as a weakly associating dimer, and the unit cell parameters are unique among the human SIRT3 structures. The inhibitor resides at the interface of the two chains related by noncrystallographic symmetry and forms several favorable contacts with the opposite chain, which complicates the design somewhat.

NH6-10 formed a covalent adduct with the cosubstrate NAD^+^ as expected. We aligned the structure to previously published SIRT2 (PDB 6NR0) and SIRT1 (PDB 4I5I) structures. While most residues near the NH6-10 binding sites in the three sirtuins are identical, we identified two residues, P297 and E325, that can differentiate SIRT3 from SIRT1 and SIRT2. P297 of SIRT3 interacts with the benzene ring of NH6-10. In the SIRT1 and SIRT2 structures, the residues corresponding to SIRT3 P297 are N417 and S238, respectively, which cannot interact with the benzene ring of NH6-10 (Figure 2). The interaction between the benzene ring of NH6-10 and P297 of SIRT3 can explain the inhibition trends we observed for several SIRT3 inhibitors. For example, when the Cbz group in NH4-10 is changed to an acetyl group, leading to SJ-112A (Figure S3), the SIRT3 inhibition is decreased. This is one of the major reasons that we kept the Cbz group at the N-terminal to efficiently target SIRT3.

The second SIRT3-unique residue, E325, likely interacts with the positively charged triethylammonium at the C-terminus of NH6-10. The distance may not be ideal, but such an interaction may become stronger for SJ-106C with a slightly longer linker. The corresponding residues are Q267 in SIRT2 (Figure 2) and R446 in SIRT1. The E325 residue of SIRT3 can explain the potency of many of our inhibitors. For example, NH4-10 inhibits SIRT3 with an IC_50_ value of 0.67 μM, while a very similar compound, NH4-9, with an “O” replacing the “NH” in NH4-10, does not inhibit SIRT3 even at 83 μM (Figure 1). This is because the “NH” of NH4-10 is positively charged at physiological pH and interacts with E325 while NH4-9 could not. Similarly, the favorable charge–charge interaction with E325 also explains why the positively charged SIRT3 inhibitors shown in Figure 1 (NH4-10, NH6-10, SJ-105, and SJ-106C) are better than those that do not have a positively charged group at the C-terminus (NH4-1, NH4-2, and NH4-3). It was fortunate that positively charged structures are advantageous for SIRT3 inhibition because positively charged structures should increase aqueous solubility and mitochondrial targeting, which are our major objectives. Overall, the structure of SIRT3 in complex with NH6-10 provided a nice explanation for the activities of the SIRT3 inhibitors. In other words, we have identified structural features that are important for engaging SIRT3, but these features do not exclude binding to SIRT1/SIRT2 because thiomyristoyl-lysine alone is enough and these other structural features do not contribute much to SIRT1/SIRT2 binding (especially for SIRT2).

### SJ-106C Inhibits the Proliferation of Various Cancer Cell Lines with the Strongest Effect in DLBCL Cells

**Figure 3:**
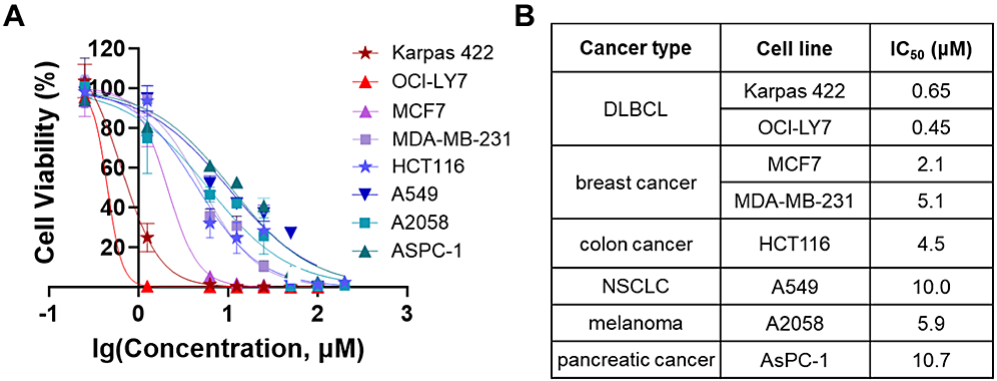
SJ-106C inhibits the viability of various cancer cells, particularly in DLBCLs. (A) Relative cell viability of the indicated cells was assessed after treatment with increasing concentrations of SJ-106C for 72 h. (B) Calculated IC_50_ values (μM) of SJ-106C for 72 h in various cancer cells.

With SJ-106C, which has improved SIRT3 inhibition potency and aqueous solubility, we next investigated whether it can inhibit the proliferation of DLBCL cell lines such as the previously reported SIRT3 inhibitor YC8-02. In addition to DLBCL, we used various other cancer cell lines, including breast cancer, colon cancer, nonsmall cell lung cancer (NSCLC), melanoma, and pancreatic cancer cells. The cells were treated with various concentrations of SJ-106C for 72 h. While antiproliferative effects of SJ-106C were observed in all the cancer cell lines, the highest cytotoxicity was observed in the two DLBCL cell lines, OCI-LY7 and Karpas 422 (Figure 3), with cellular IC_50_ values of less than 1 μM. This is consistent with previous observation that DLBCL cells are addicted to SIRT3 and thus are more sensitive to SIRT3 inhibition.

### Sirtuin Inhibition Is Important for the Inhibitory Effect of SJ-106C in DLBCL Cells

**Figure 4:**
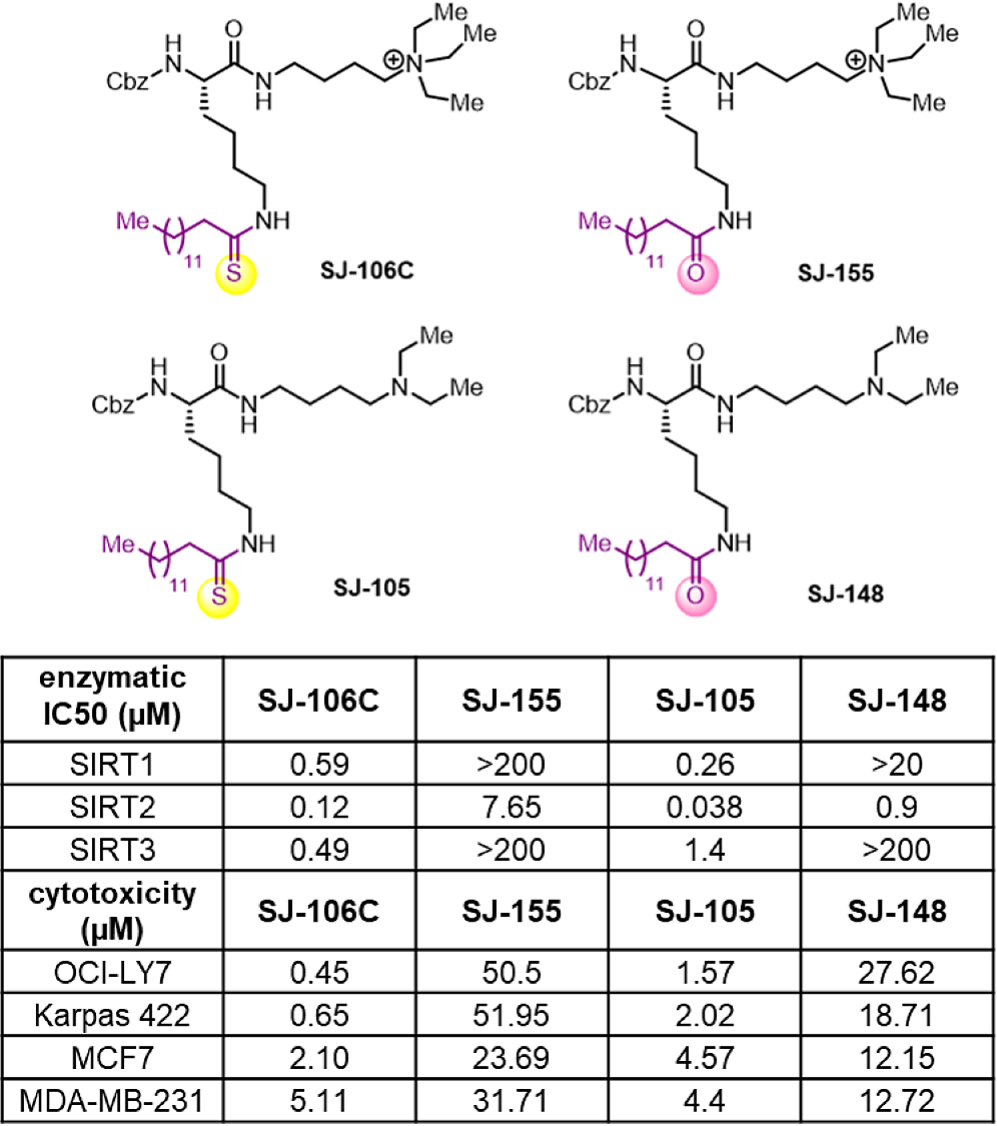
Negative control compound, SJ-155, lost its ability to inhibit SIRT1/2/3 (compared to SJ-106C). Enzymatic activity was detected using an HPLC assay.

As far as we know, we are the first to introduce quaternary ammonium into sirtuin inhibitors. We wanted to ensure that the inhibitory effect of SJ-106C was not due to undesirable toxic effects from quaternary ammonium. Initial assurance came from testing SJ-105, a compound that is highly similar to SJ-106C except that the quaternary ammonium is changed to a tertiary amine (Figure 4). Similar to SJ-106C, SJ-105 exhibits a high potency against DLBCL cell lines (Figure 4). To further confirm that the inhibitor effect of SJ-106C was due to sirtuin inhibition, we synthesized a control compound, SJ-155, in which the thiomyristoyl group in SJ-106C was replaced with a myristoyl group (Figure 4). This single-atom change from S to O essentially eliminated SIRT1 and SIRT3 inhibition and dramatically decreased SIRT2 inhibition. Accordingly, SJ-155, which exhibited very little SIRT1/2/3 inhibition, also demonstrated very weak inhibitory effects on DLBCL cell lines, supporting that the inhibition of SIRT1/2/3 is important for SJ-106C’s effect in DLBCL cells and the quaternary ammonium used is not toxic.

### SJ-106C Is a Mitochondria-Targeting Compound That Inhibits SIRT3 in DLBCL Cells

**Figure 5:**
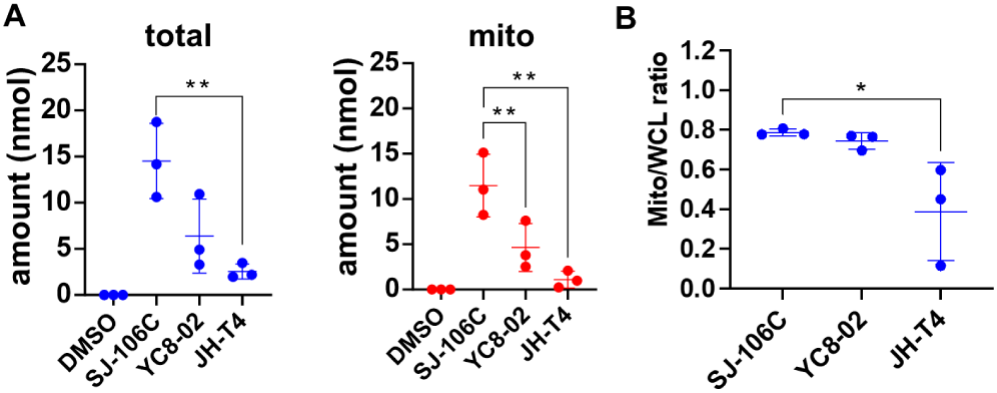
SJ-106C as a mitochondria-targeting SIRT3 inhibitor. (A) 20 million of Karpas 422 cells were treated with SJ-106C, YC8-02, or JH-T4 at 5 μM for 6 h. Quantification of compound amount (in nmol) in whole cell lysate and mitochondrial fractions was detected by LC-MS; (B) the ratio of the inhibitor amount in mitochondria to that in whole cell lysate for each inhibitor was calculated based on the compound amount in each exacted portion normalized to the exact volume of extraction. Data represent three independent experiments and are presented as mean ± s.d. *p < 0.05, **p < 0.01.

The triethylammonium group in SJ-106C was introduced with the intention of increasing the mitochondrial targeting. To determine whether this is the case, we quantified the concentrations of inhibitors in whole cell lysates and purified mitochondria from treated DLBCL cells. We compared SJ-106C to YC8-02 (a SIRT3 inhibitor with a TPP mitochondrial-targeting motif) and JH-T4 (a SIRT3 inhibitor without a mitochondrial-targeting motif). We treated Karpas 422 cells with each inhibitor for 6 h and then extracted the corresponding whole cell lysates and mitochondria fractions. The marker proteins VDAC and α-tubulin were assessed using Western blot to confirm the purity of the mitochondrial fractionation (Figure S4). Among the three inhibitors, SJ-106C achieved the highest levels in both the whole cell lysate and the mitochondria (Figure 5). Although YC8-02 has a low ablility to penetrate cells, the ratio of mitochondrial YC8-02 was similar to SJ106C and better than that of JH-T4 (Figure 5). Therefore, the triethylammonium group seems to be as efficient as the TPP group in mitochondria-targeting of sirtuin inhibitors (Figure 5).

**Figure 6:**
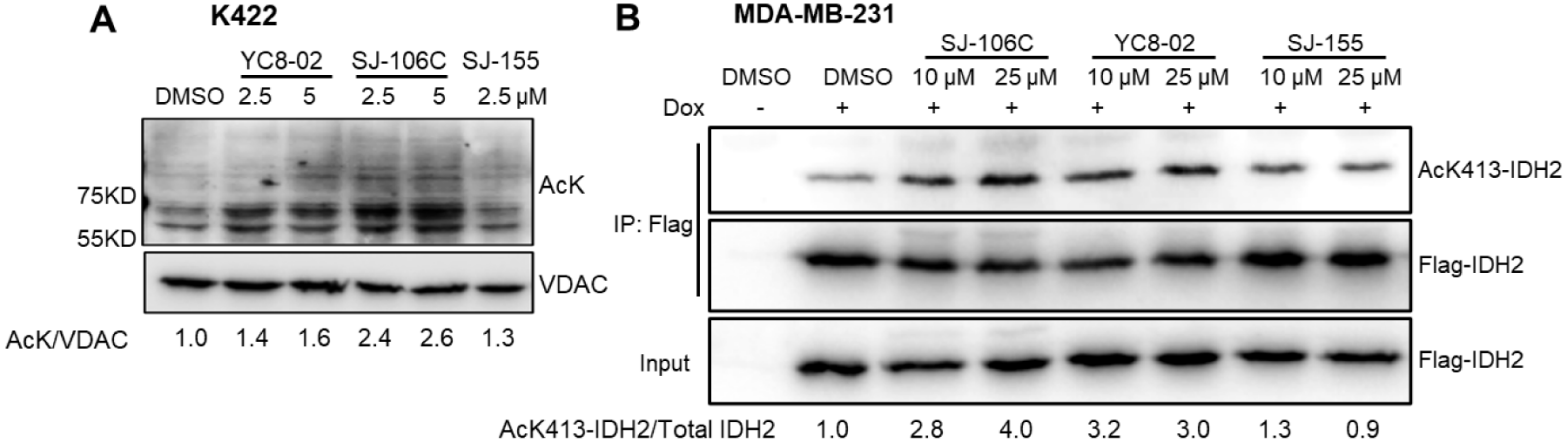
SJ-106C inhibits SIRT3 in the cells. (A) Western blot and densitometry analysis of mitochondrial acetylation from Karpas 422 cells (1 million/mL) were treated with indicated compounds for 12 h. The mitochondria were isolated and blotted for acetyl-lysine. Western blots were quantified by densitometry, normalized to the intensity of the corresponding VDAC, and then further normalized to the DMSO group. (B) Western blot analysis of acetylated IDH2 (K413) after immunoprecipitation of Flag-tagged IDH2 in MDA-MB-231 cells treated with DMSO, SJ-106C, YC8-02, or SJ-155 at the indicated concentrations for 6 h. This cell line expressed Flag-IDH2 in a doxycycline-inducible manner. Western blots were quantified by densitometry, normalized to the intensity of the corresponding IP Flag-IDH2, and then further normalized to the DMSO group.

Next, we validated whether SJ-106C could inhibit SIRT3 in DLBCL cells. As expected, treatment of DLBCL Karpas 422 cells with SJ-106C at 2.5 and 5 μM for 12 h increased mitochondrial protein acetylation (Figure 6A, see full blots in Figure S9A). In these experiments, we used concentrations of SJ-106C higher than cellular IC_50_ values but shorter treatment time (12 h instead of 72 h used in cellular toxicity assay) to increase acetylation but avoid cell death. Treatment of YC8-02 at 5 μM also increased the acetylation level, while SJ-155, which does not inhibit SIRT3 in vitro, did not increase acetylation (Figure 6A, see full blots in Figure S9A). We further validated that SJ-106C could inhibit SIRT3 in cells using the MDA-MB-231 cell line expressing Flag-tagged IDH2 in a doxycycline-inducible manner. We assessed acetylation levels of IDH2, a reported SIRT3 substrate,^16^ in cells treated with SJ-106C, YC8-02, and the control compound SJ-155. Cells were treated with each inhibitor for 6 h, and the Flag-tagged IDH2 was pulled down by immunoprecipitation. Acetylation levels were assessed by using a specific AcK413-IDH2 antibody. SJ-106C and YC8-02 increased the acetylation level of IDH2, whereas SJ-155 did not (Figure 6B, see full blots in Figure S9B).

SJ-106C inhibits SIRT1 and SIRT2 in vitro. Thus, we also assessed the inhibition of SIRT1 and SIRT2 in the cells. We examined acetylation levels of known substrates of SIRT1 and SIRT2, namely, p53 and α-tubulin, respectively. As expected, SJ-106C could inhibit SIRT1 and SIRT2 in cancer cells (Figures S5 and S6). Interestingly, EX-527 (a selective SIRT1 inhibitor) and TM (a selective SIRT2 inhibitor) exhibit much lower inhibition in DLBCL cells compared to that of SJ-106C, indicating that SJ-106C’s robust antiproliferative activity is mainly through SIRT3 inhibition in DLBCL cells (Figure S6B).

### SJ-106C Demonstrates In Vivo Antitumor Efficacy against DLBCL

**Figure 7:**
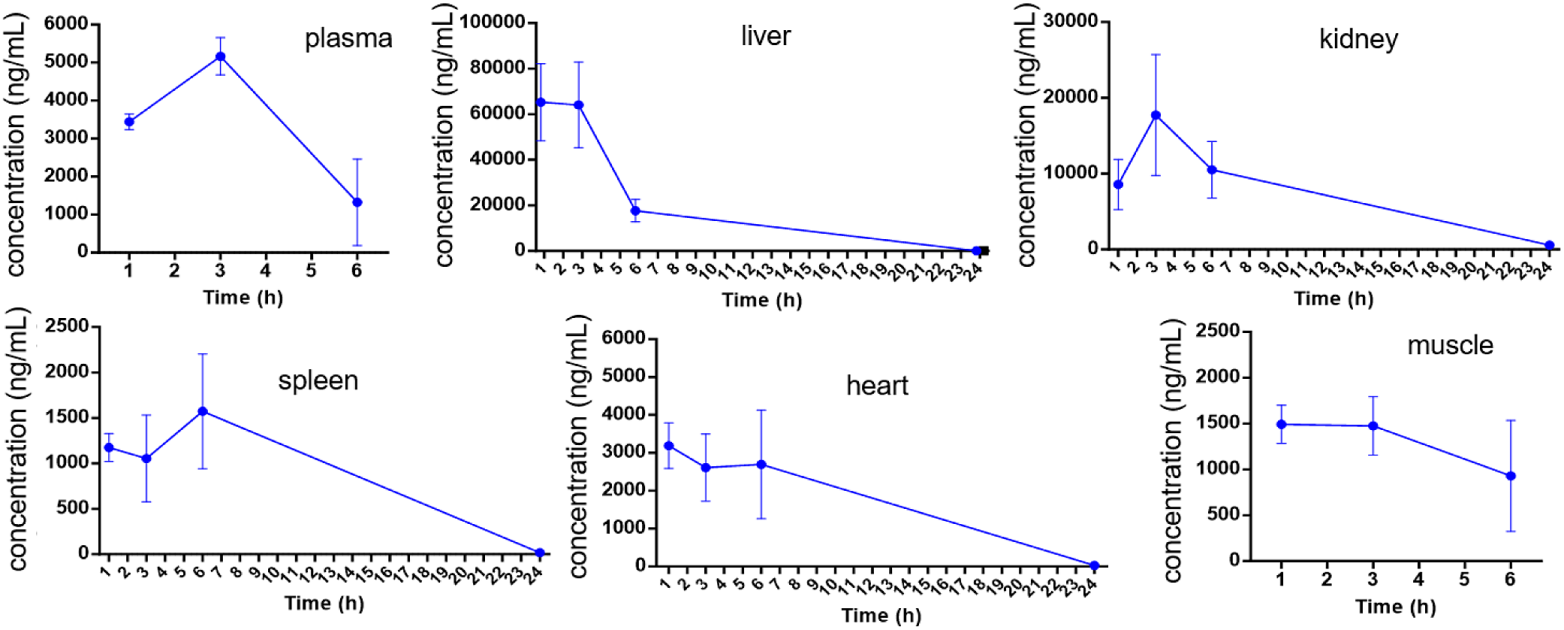
Pharmacokinetics of SJ-106C in NSG mice. SJ-106C was intraperitoneally (IP) injected at 100 mg/kg. Blood and various organs were collected at specified time points of 1, 3, 6, and 24 h after SJ-106C administration. Plasma isolation, tissue lysis, and extraction of SJ-106C with methanol were performed for LC-MS detection (*n* = 3 for each time point).

Our in vitro and cell-based assays revealed the promising inhibitory effect of SJ-106C in DLBCL. To further evaluate its efficacy in vivo, we first conducted pharmacokinetic studies by intraperitoneally (IP) injecting SJ-106C (100 mg/kg) into NSG (NOD scid gamma) immunodeficient mice. Blood and organs including the liver, kidneys, spleen, heart, and muscle were collected at 1, 3, 6, and 24 h, and the compounds were extracted for LC-MS detection. At 3 h, SJ-106C was enriched in the liver and kidneys, followed by the plasma, and was also detectable in the heart, spleen, and muscle, then being eliminated from the body 24 h after treatment (Figure 7). The plasma concentration at 3 h exceeded 4000 ng/mL (∼5 μM), significantly surpassing the IC_50_ value of SJ-106C in DLBCL cells (Figure 3), indicating favorable bioavailability of SJ-106C.

We next assessed the toxicity of SJ-106C at 50 and 100 mg/kg through daily IP injections for five consecutive days in NSG mice before xenograft studies. SJ-106C had a minimal effect on body weight (Figure S8) and no obvious decrease in the physical activity of mice, indicating minimal toxicity of SJ-106C at 50 and 100 mg/kg daily dose.

**Figure 8:**
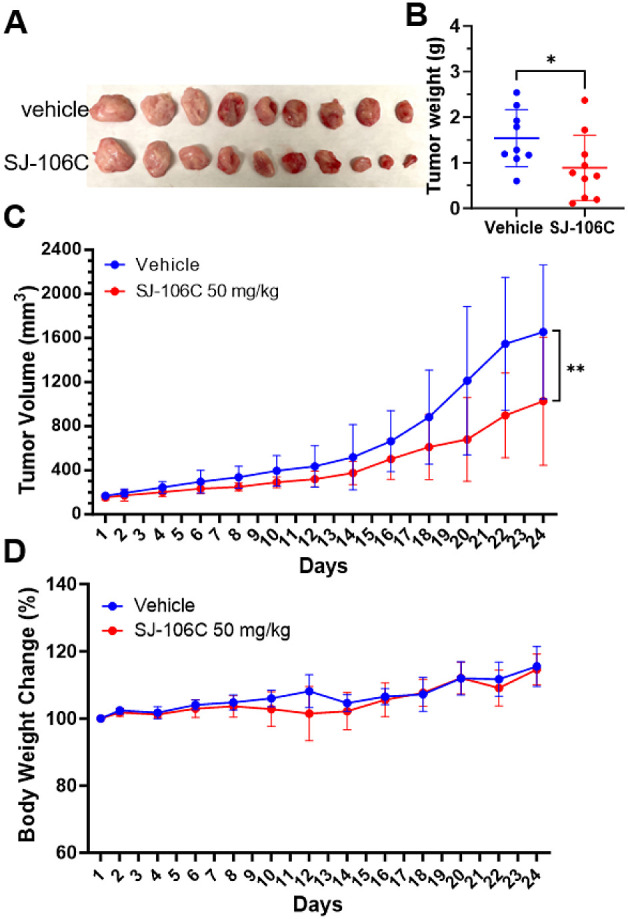
Effects of SIRT3 inhibitors on the OCI-LY7 tumor xenograft model. (A) Image of the OCI-LY7 xenograft tumors dissected from NSG mice treated with SJ-106C or vehicle control. (B) Tumor weights in different groups of mice were measured. (C) Tumor growth curves of the OCI-LY7 tumor xenografts with intraperitoneal administration of vehicle, 50 mg/kg SJ-106C five times per week for 24 days (*n* = 5 mice per group). (D) Average body weight of mice in different groups. Data are shown as mean ± sd of 5 mice per group. *P*-values were determined by Student’s *t* test. *p < 0.05, **p < 0.01.

We then established a DLBCL xenograft mouse model using the OCI-LY7 cells. OCI-LY7 tumor xenografts were treated with intraperitoneal administration of vehicle control or 50 mg/kg SJ-106C five times per week for 24 days. SJ-106C treatment inhibited tumor growth throughout the administration period (Figure 8A–C). No obvious weight loss or discomfort was observed in the SJ-106C-treated mice (Figure 8D). Our in vivo data suggest that SJ-106C exhibits anti-DLBCL efficacy in mice with minimal toxicity.

## Discussion

With the goal of developing SIRT3 small-molecule inhibitors with improved solubility while retaining mitochondrial targeting, we designed and synthesized a series of mechanism-based inhibitors of SIRT3. Through this effort, we identified SJ-106C as the most promising SIRT3 inhibitor in this series. SJ-106C displayed superior aqueous solubility compared to previously reported YC8-02 and reached higher concentrations in cells. Similar to YC8-02, it efficiently localizes to mitochondria in cells. SJ-106C was also slightly more potent than YC8-02 based on in vitro enzymatic and cell culture assays for SIRT3 inhibition. SJ-106C showed superior PK/PD properties in mice and was well tolerated. We thus provide an improved SIRT3 inhibitor more suitable for assessing the impact of these drugs against DLBCLs.

It has been highly challenging to develop inhibitors that are highly selective for SIRT3 because of structural conservation in the enzyme pockets among SIRT1, SIRT2, and SIRT3. We first invented the method of increasing SIRT3 inhibition using the mitochondrial targeting compound, YC8-02,^6a^ which can inhibit SIRT3 in vivo. Recently, another strategy has been reported for the SIRT3 inhibition in cells,^6b^ which is achieved by directing the inhibitors to the mitochondria through the incorporation of mitochondria-targeting peptide sequences, further supporting that mitochondrial targeting is an effective strategy for targeting SIRT3. Our current study further advanced this strategy and revealed important findings for future development of SIRT3 inhibitors or mitochondrial targeting agents in general.^14,15^ YC8-02 used TPP as the mitochondrial targeting motif. While TPP is widely used to target compounds to the mitochondria,^14,15^ it shows high hydrophobicity and impaired YC8-02 solubility in aqueous solutions, leading to practical limitations. There is a debate regarding whether TPP may induce mitochondrial depolarization and cause nonspecific toxicity.^17,18^ Therefore, here, we used triethylammonium as the mitochondrial-targeting motif. To our delight, the modification with triethylammonium worked very well and correspondingly SJ-106C had much improved aqueous solubility and cell permeability. Our results showed that both TPP and triethylammonium showed similar mitochondrial targeting efficiency when conjugated with a sirtuin inhibitor. Moreover, SJ-106C had greater potency in enzymatic inhibition and DLBCL cell killing experiments but lower toxicity in vivo. The effects on DLBCL were dependent on its ability to inhibit sirtuins, as the structurally similar negative control compound SJ-155 had little effect, indicating that the mitochondrial-targeting motif moiety is not toxic to DLBCL cells.

SJ-106C was also slightly more potent than YC8-02 in vitro. To understand the improved potency, we obtained X-ray crystallography structures of human SIRT3 in a complex with an SJ-106C analog (NH6-10). This structure revealed two small molecule–protein interactions that may contribute to its better potency: the Cbz group packs against P297 of SIRT3, and the positively charged triethylammonium may interact with the negatively charged E325 of SIRT3. These features could be further capitalized on in future SIRT3 inhibitor design campaigns to improve potency and SIRT3 selectivity, as these two residues are different in SIRT1 and SIRT2.

While poor membrane permeability could be a concern for quaternary ammonium compounds, SJ-106C has effective intracellular accumulation and consistent biochemical activities. We suspect that its cell permeability could be due to a combination of two things. One is active transport through organic cation transporters, and the other could be that the three ethyl groups are relatively hydrophobic (compared to trimethylammonium or ammonium with three hydrogen atoms), which facilitate passive diffusion.

Both YC8-02 and SJ-106C treatment can inhibit DLBCL tumor growth in vivo. While there is no further improvement in potency compared to that of YC8-02, SJ-106C has several advantages. The solubility is much approved, and the in vivo application does not require a hydrophobic carrier anymore. Furthermore, minimal toxicity was observed in SJ-106C-treated mice at 50 mg/kg, while YC8-02 had to be used at lower doses due to toxicity at 50 mg/kg.

There is a pressing need to develop new therapeutic strategies for DLBCL, which are highly proliferative and heterogeneous. The identification of SIRT3 as a broadly relevant non oncogene addiction among DLBCLs suggests that SIRT3 inhibition could be an effective therapeutic strategy for DLBCL. Importantly, SIRT3 inhibition did not harm normal tissues, thus providing a significant therapeutic window for dosing these compounds. The results presented here further support this notion. In particular, we found that SJ-106C is the most potent in DLBCL cells among all cancer cell lines tested. Even though SJ-106C was distributed to various organs, including the heart and muscle, there was no obvious toxicity in mice even after 3 weeks of treatment. The promising results motivate efforts to further develop SIRT3 inhibitors with improved potency, selectivity, and PK properties.

## Materials and Methods

### Reagents and Antibodies

The syntheses of compounds are described in the Supporting Information. All compounds are >95% pure by HPLC analysis, and the HPLC trace for SJ-106C is included in the Supporting Information. Trichostatin A (TSA) was purchased from Sigma-Aldrich (USA). All compounds were dissolved in dimethyl sulfoxide (DMSO). The antibodies used include the VDAC antibody (#4661), α-tubulin antibody (#2144), acetylated-lysine antibody (#9441), acetyl-p53 antibody (K382) (#2525), p53 antibody (#2527), and HRP-linked antirabbit IgG antibody (#7074), all purchased from Cell Signaling Technology (USA). The GAPDH antibody (sc-47724 HRP) was purchased from Santa Cruz Biotechnologies. The acetyl K413-IDH2 antibody (AC0004) was sourced from GeneTel Laboratories. The Flag M2 antibody (F1804) and acetyl-α-tubulin antibody (MABT868) were obtained from Sigma-Aldrich. The Cy3 Goat-antimouse antibody (A10521) was purchased from Invitrogen (USA).

### Cloning, Expression, and Purification of Human Sirtuins

Human SIRT1, SIRT2, and SIRT3 were expressed and purified as previously described.^20^

### In Vitro Deacetylase Activity Assay for Sirtuins

H3K9Ac and H3K9 peptides were all synthesized as previously described, using standard solid-phase peptide synthesis.^21^ Various concentrations (0.005, 0.027, 0.133, 0.667, 3.333, 16.667, and 83.333 μM) of small molecule inhibitors were added to solutions containing 20 mM Tris-HCl (pH 8.0), 1 mM NAD^+^, 1 mM dithiothreitol (DTT), and 0.05 μM SIRT1, 0.1 μM SIRT2, or 0.2 μM SIRT3, respectively. The reaction mixtures were incubated at 37 °C for 15 min. Then, 10 μM H3K9Ac (KQTARK(Ac)STGGWW) peptide was added to initiate the reactions, and the reactions were then incubated at 37 °C in a total volume of 60 μL (5 min for SIRT1 and SIRT2, 10 min for SIRT3). The conversion of the H3K9Ac substrate in each reaction was less than 20%. To stop the reactions, an equal volume of acetonitrile was added. After quenching, the samples were centrifuged at 17 000 g for 20 min to remove any precipitated proteins. The cleared supernatant was subjected to HPLC using a reverse-phase C18 column (Kinetex XB-C18 100A, 100 mm × 4.60 mm, 2.6 μm, Phenomenex). A gradient of two solvents (A: 0.1% trifluoroacetic acid in water; B: 0.1% trifluoroacetic acid in acetonitrile) was employed. The gradient started with 0% B for 2 min, followed by an increase from 0% to 20% B in 2 min, from 20% to 40% B in 13 min, and finally from 40% to 100% for 2 min, all at a flow rate of 0.5 mL/min. Peak areas of free H3K9 and H3K9Ac were quantified based on HPLC UV absorption traces at 280 nm. The conversion rate was calculated as the fraction of the free H3K9 peptide from the total peptide. All reactions were performed in duplicate, and IC_50_ values were calculated by using Prism 7 software.

### Cell Lines and Culture

MCF7, MDA-MB-231, A2058, and AsPC-1 cell lines were cultured in Dulbecco’s modified Eagle’s medium (DMEM, Invitrogen) supplemented with 10% (v/v) fetal bovine serum (FBS, Gibco). HCT116 cells were maintained in McCoy’s 5A medium (Invitrogen) supplemented with 10% FBS. A549 cells were grown in RPMI-1640 medium (Invitrogen) supplemented with 10% FBS. OCI-LY7 cells were cultured in IMDM (Iscove’s modified Dulbecco’s medium) with 10% FBS. Karpas 422 cells were cultured in the RPMI-1640 medium containing 10% FBS, HEPES (1 M, 1:100, Invitrogen) and Glutamine (200 mM, 1:100, Invitrogen).

### Cytotoxicity Assay

For DLBCL cell lines, Karpas 422 and OCI-LY7 cell lines were seeded at a density of 20 000 cells/well in white 96-well plates (CELLSTAR, VWR, USA), while other cell lines were seeded at a density of 5000 cells/well. Test inhibitors were serially diluted in each culture medium. The mixture was added to the cells, and the plates were incubated at 37 °C for 72 h. Cytotoxicity was measured by quantitating the ATP present, using a CellTiter-Glo luminescence cell viability assay (Promega) according to the manufacturer’s instructions. The IC_50_ values for the concentrations were calculated by using the nonlinear fit variable slope model (GraphPad Software).

### Western Blot Analysis

Proteins were separated on a 12% SDS-PAGE gel and transferred onto PVDF membranes. Following transfer, membranes were incubated overnight with specific primary antibodies at 4 °C. Then, HRP-linked antirabbit IgG was used as the secondary antibody. Protein detection was performed using an ECL reagent (Pierce Biotechnology Inc., USA). The densitometry of Western blot results was measured by using ImageJ software.

### Drug Uptake in Mitochondria

Karpas 422 cells (1 million/mL, total 20 million cells) were treated with SJ-106C, YC8-02, or JH-T4 at 5 μM for 6 h. Approximately 4 million cells were used for whole cell extraction, and approximately 16 million cells were used for mitochondria extraction. Mitochondria were extracted using the Qproteome Mitochondria Isolation Kit (Qiagen, Germany) following the manual instructions. The exact volume of each extracted portion (whole cell lysate or mitochondria) was marked for LC-MS quantification analysis. Each sample with an exact volume was reserved and then lysed for Western blot analysis as quality controls. For LC-MS detection, the whole cells or mitochondria pellets were lysed with 120 μL of acetonitrile and centrifuged at 17 000g for 10 min to remove precipitated proteins. Then, 100 μL of the final supernatant was loaded onto LC-MS for compound detection. The compound concentration was determined by comparing the ion peak area of each inhibitor with the corresponding internal standard curves. As for the quality control of Western blot analysis, the protein levels of VDAC and α-tubulin were quantified using ImageJ. Based on the compound amount in each exacted portion normalized to the exact volume of extraction, the ratio of inhibitor concentration in mitochondria to whole cells was calculated. All samples were performed in triplicates.

### Immunoprecipitation of Flag-Tagged IDH2 in MDA-MB-231 Cells

MDA-MB-231 cells expressing flag-tagged IDH2 in a doxycycline-inducible manner were used to detect the inhibition of SIRT3 deacetylase activity in cells as previously.^19^ To induce the IDH2-Flag overexpression, cells were treated with doxycycline (1 μg/mL) for 48 h. Then, the cells were exposed to indicate concentrations of SJ-106C, YC8-02, or SJ-155 for 6 h. After collection, cell lysis was performed using a buffer containing 25 mM Tris, pH 7.4, 150 mM NaCl, 10% glycerol, 1% Nonidet *p*-40, and 1× protease inhibitor cocktail. Immunoprecipitation of flag-tagged proteins was carried out using the lysates, and the acetylation levels of IDH2 were detected through immunoblotting with an antirabbit AcK413-IDH2 antibody.

### Immunofluorescence Assay

Karpas 422 cells (1 × 10^6^/mL) were treated with either DMSO control or indicated concentrations of SJ-106C, YC8-02, SJ-155, or TM for 6 h. The cells were collected and washed three times with ice-cold PBS and fixed with ice cold methanol for 10 min. To permeabilize the membrane, the cells were treated with 0.1% Triton-X in PBS for 10 min. After washing three times with ice-cold PBS, the cells were blocked with 1% BSA in TBST (25 mM Tris-HCl, pH 7.4, 150 mM NaCl, and 0.1% Tween 20) for 30 min. The cells were then incubated with the Ac-α-tubulin antibody (1:100) in 1% BSA overnight at 4 °C. After washing with TBST buffer three times, the cells were treated with the Cy3 conjugated secondary antibody (1:1000) in 1% BSA in TBST and incubated in the dark at room temperature for an hour. Following three washes with TBST, the cells were collected, and the nuclei was stained with DAPI Fluoromount-G (Southern Biotech, 0100–01, USA), and then mounted with 35 mm glass bottom dishes (MatTek, USA). The cells were imaged using a Cytation 5 Cell Imaging Reader set up with a 20× objective, using the same settings for all samples (*n*= 3 or 6 of random microscopic fields), and the images were processed using ImageJ software.

### Pharmacokinetics of SJ-106C in Mice

8-week-old male NSG (NOD scid gamma) mice from the Jackson Laboratory were used for the pharmacokinetic study. To prepare 100 mg/kg SJ-106C drug solutions for intraperitoneal (IP) injection, SJ-106C is initially mixed with 10% DMSO of the final volume, followed by the addition of 90% PBS of the final volume. The mixture is thoroughly mixed to make the final solutions for intraperitoneal (IP) injection. The mice were divided into groups (0, 1, 3, 6, and 24 h, each group *n* = 3) and IP injected with either vehicle (10% DMSO and 90% PBS) or SJ-106C at 100 mg/kg. For the plasma collection, at each indicated time point, 150–350 μL of terminal blood were collected from the heart of each mouse. Blood samples were centrifuged at 21 000 g for 15 min at 4 °C. The supernatant was transferred to new tubes, and an equal volume of methanol was added to extract the small molecules. The tubes were vortexed thoroughly and centrifuged at 21 000 g for 15 min at 4 °C. The collected supernatant was again centrifuged at 21 000 g for 15 min at 4 °C. Finally, 100 μL of the supernatant was loaded onto the LC-MS for compound detection. For the collection of tissue samples, at each time point, the spleen, liver, kidneys, heart, and muscle were collected, weighed, and homogenized using a Tissue Lyser LT instrument (Qiagen). The homogenates were centrifuged at 21 000 g for 20 min at 4 °C, and the supernatants were dried in a Speedvac vacuum concentrator (C100) at room temperature. The dried samples were dissolved in LC-MS grade methanol in proportion to the mass of each tissue sample. The samples were centrifuged again at 21 000 g for 15 min at 4 °C. Subsequently, 100 μL of the final supernatant was loaded onto the LC-MS for compound detection. The compound concentration was determined by comparing the ion peak area of SJ-106C to the internal standard curve.

### Drug Toxicity Study

The in vivo toxicity study was conducted using 8-week-old male NSG mice. Mice were divided into two groups and injected with either vehicle (10% DMSO and 90% PBS) or SJ-106C at 50 and 100 mg/kg (each group *n* = 5). Each mouse was weighed and IP injected daily for 5 days, and the weights of all mice were recorded before each injection. The toxicity study stopped on day 6 after the last measurement of the mouse body weight.

### OCI-LY7 Xenograft Mouse Model

A DLBCL OCI-LY7 cell-derived xenograft mouse model was established by following federal and institutional guidelines approved by the Institutional Animal Care and Use Committee of Cornell University. 10 million OCI-LY7 cells were suspended in 100 μL of PBS and Matrigel Basement Membrane Matrix (Corning, LDEV-free 354234, USA) with a ratio of 1:1 and then subcutaneously injected into both flanks of eight-week-old male NSG mice. Tumor volumes were measured using an electronic digital caliper and calculated using the formula: tumor volume (mm^3^) = (*L* × *W*^2^)/2, where *L* and *W* refer to the length and width of tumors, respectively. Once the tumor volumes grew to around 100 mm^3^, mice were randomly divided into three groups (each group *n* = 5) and IP treatment was initiated. Mice received daily IP injection of the vehicle (10% DMSO, 10% Kolliphor, 80% PBS), or SJ-106C (50 mg/kg)) five times per week for 24 days. Tumor volumes and body weights were measured every other day. After treatment or if the humane end point criteria were met, the mice were sacrificed by CO_2_ asphyxiation. Tumors were collected, photographed, and weighed for data collection.

### Statistical Analysis

Significance was evaluated with two-tailed Student’s *t* tests unless otherwise indicated in the figure legends unless stated otherwise. *p* values less than 0.05 were considered significant (*, *p* < 0.05; **, *p* < 0.01; ***, *p* < 0.001). The graphs and error bars show the mean ± s.d. of independent biological experiments. All statistical analyses were performed using Microsoft Office Excel or GraphPad Prism V10.

### Crystallization and Structure Determination

SIRT3 was cocrystallized with reaction intermediates derived from NAD^+^ and NH6-10 cosubstrates using the hanging drop vapor diffusion method. Thawed protein samples were centrifuged, concentrated, and then combined with stock solutions of NAD^+^ (80 mM NAD^+^ and 500 mM Tris base) and NH6-10 (100 mM NH6-10 diluted to 5.5 mM with protein storage buffer) to yield 70 μL of the reaction mixture containing 6 mg/mL SIRT3, 3 mM NAD^+^, and 0.5 mM NH6-10. The reaction mixture was incubated on ice for approximately 3 h and then used to prepare crystallization drops in a 1:1 volume ratio with reservoir solutions. The crystal yielding the reported structure grew in 100 mM Tris, pH 7.9, 100 mM MgCl_2_, 20% (w/v) PEG 8000, and 17.5% (w/v) PEG 400.

The co-crystals of SIRT3 and reaction intermediates were looped, cryocooled via plunging in liquid nitrogen, and shipped to the NE-CAT beamline 24-ID-E of the Advanced Photon Source. The crystal corresponding to the reported structure was irradiated with X-rays having a wavelength of 0.98 Å with 0.2 s exposure times and 0.2 deg oscillation widths. 750 X-ray diffraction images were recorded using a Dectris EIGER 16 M detector with a crystal-to-detector distance of 210 mm. Reflections were indexed, integrated, and scaled using XDS^22^ and Aimless.^23^ The crystal belongs to the space group *P*2_1_ and diffracted to a resolution of 1.95 Å (Table S1). Initial phases were determined by molecular replacement using SIRT3 (PDB code 4BN4) as a search model. Manual model building was conducted using COOT,^24,25^ and automated ML refinement was performed using Phenix^26^ with anisotropic *B*-factor refinement for the zinc binding site and TLS refinement^27^ for the remainder of the protein chains. Intermediate chemical structures were drawn in ChemDraw and exported in SMILES format to Phenix eLBOW^28^ for restraint files using eLBOW AM1 geometry optimization. The SIRT3 crystal structure was refined to 1.95 Å resolution with an *R*_work_/*R*_free_^29^ of 19.8/23.9% and was validated using MolProbity^30^ in Phenix and ProCheck in CCP4.^31^ Figure preparation was performed using Chimera.^32^

SIRT3 and NH6-10-derived reaction intermediates cocrystallized in the space group *P*2_1_ with two copies (chains A and B) of SIRT3 in the asymmetric unit (Figure S7A). Structural models were built and refined for residues 122–391 of chain A and residues 122–395 of chain B. Internally disordered regions displaying high *B*-factors include residues 169–176 and 198–203. Electron density maps show that both SIRT3 molecules contain a ligand occupying both NAD^+^ and peptide substrate binding sites and a metal ion assigned as zinc ligated by Cys256, Cys259, Cys280, and Cys283. Domain motion analysis^33^ shows domain closures of approximately 19–24° relative to apo SIRT3 structures^34^ (Figure S7B).

The shape of the active site 2*F*o-*F*c omit electron density and the presence of continuous electron density beyond the NH6-10 lysine *N*_Z_ atom suggest bound reaction intermediates (Figure S7C,D). The electron density near the NMN C1’ atom is inconsistent with intermediate I.^34,35^ Intermediates II and III can be placed into active site *F*o-*F*c electron density of both chains; however, Intermediate III was modeled in chain A (Figure S7C) and intermediate II in chain B (Figure S7D) on the basis of comparisons of residual *F*o-*F*c electron density observed after refinements with both intermediates. Electron density is strong for the modified ADP ribose and peptidyl moieties but very weak beyond the myristoyl group carbon 2 of modeled Intermediate III (Figure S7C) and the myristoyl group carbon 7 of modeled Intermediate II (Figure S7D).

## Data Availability

Data are deposited to the Protein Data Bank (PDB ID 9CBT). Authors will release the atomic coordinates upon article publication.

## Supporting Information Available

Supporting figures and table, general methods for synthesis, compound characterization data, references, NMR spectra, and HPLC purity determination for SJ-106C (PDF)
Molecular formula strings spreadsheet (CSV)

## Abbreviations

Cbz: benzyl carbamate
DLBCL: diffuse large B-cell lymphomas
DOX: doxycycline
DMSO: dimethyl sulfoxide
DTT: dithiothreitol
DMEM: Dulbecco’s modified Eagle’s medium
ECL: enhanced chemiluminescence
FBS: fetal bovine serum
GC: germinal center
GAPDH: glyceraldehyde-3-phosphate dehydrogenase
H3K9: histone H3 lysine 9
H3K9Ac: histone H3 lysine 9 acetylation
HEPES: (4-(2-hydroxyethyl)-1-piperazineethanesulfonic acid
IDH2: isocitrate dehydrogenase 2
IP: intraperitoneally
IMDM: Iscove’s modified Dulbecco’s Medium
LDEV: lipid droplet encapsulated vesicle
NAD^+^: nicotinamide adenine dinucleotide
NHL: non hodgkin’s lymphomas
NSCLC: nonsmall cell lung cancer
NSG: NOD scid gamma
PEG: polyethylene glycol
PVDF: polyvinylidene fluoride
PBS: phosphate-buffered saline
R-CHOP: rituximab + cyclophosphamide + hydroxydaunorubicin + oncovin + prednisone
ROS: reactive oxygen species
SDS-PAGE: sodium dodecyl sulfate polyacrylamide gel electrophoresis
TPP: triphenylphosphonium
TSA: trichostatin A
VDAC: voltage-dependent anion-selective channel
