# Supplementary material for: A Mitochondria-Targeting SIRT3 Inhibitor with Activity against Diffuse Large B Cell Lymphoma
Source: J Med Chem. 2024 Aug 27;67(17):15428–37. doi: 10.1021/acs.jmedchem.4c01053 (PMC11403614; doi:10.1021/acs.jmedchem.4c01053)
Supplement: Supplementary file 1 — jm4c01053_si_001.pdf [file jm4c01053_si_001.pdf]

Supporting Information

A mitochondria-targeting SIRT3 inhibitor with activity against diffuse large B cell lymphoma

Sadhan Jana,[1#] Jialin Shang,[1#] Jun Young Hong, [1] Michael K. Fenwick, [1,4] Rishi Puri,[3] Xuan Lu, [1] Ari M. Melnick,[2,*] Meng Li,[2,*] Hening Lin[1,5,*]

[1]Department of Chemistry and Chemical Biology, Cornell University, Ithaca, NY 14853, USA

[2]Department of Medicine, Division of Hematology & Medical Oncology, Weill Cornell Medicine, New York, NY, USA.

[3]College of Veterinary Medicine, Cornell University, Ithaca, NY 14853, USA.

[4]Current Address: Seattle Children's Research Institute, 307 Westlake Ave N, Seattle, WA, 98109

[5]Howard Hughes Medical Institute; Department of Chemistry and Chemical Biology; Department of Molecular Biology and Genetics, Cornell University, Ithaca, NY 14853, USA

[#] These authors contribute equally to this work

[*]Corresponding authors: hl379@cornell.edu (H.L.); mel2013@med.cornell.edu (M.L.); amm2014@med.cornell.edu (A.M.M.)

Table of Contents

1	Supplementary Figures	S2-S10
2	Supplementary Table	S11
3	General methods for synthesis	S12
4	Compound Characterization Data	S12-S26
5	References	S26
6	NMR Spectra	S28-S55
7	HPLC purity determination for SJ-106C	S56

Supplementary Figures

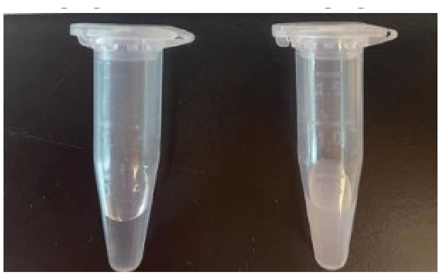

Figure S1. Approximately 15 mg of SJ-106C or YC8-02 was dissolved in ~300 µL of the vehicle (10% DMSO 30 µl + 90% PBS 270 µl), diluted to a final concentration of 50 mg/mL for a dosage of 100 mg/kg in mice studies.

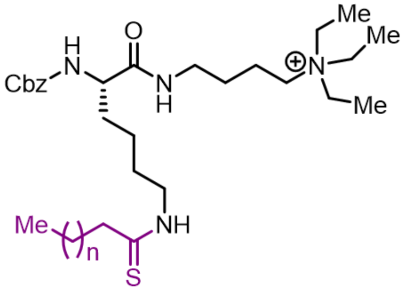

IC50 (µM)	n = 11 SJ-106C	n = 9 SJ-202	n = 7 SJ-204	n = 5 SJ-213	n = 3 SJ-206
**SIRT1**	0.59	5.4	5.81	1.31	17.3
**SIRT2**	0.12	0.73	0.07	0.13	1.7
**SIRT3**	0.49	58.4	>200	11	>100

Figure S2. Structures and activities of SJ-106C analogs with shorter thioacyl chains.

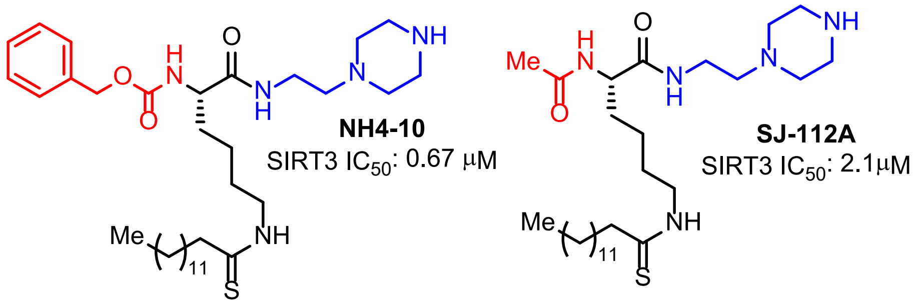

Figure S3. The structures and SIRT3 IC_{50} values of two inhibitors NH4-10 and SJ-112A. The lack of the aromatic ring in SJ-112A decreases the SIRT3 inhibition potency likely because the aromatic ring interacts with Pro297 of SIRT3.

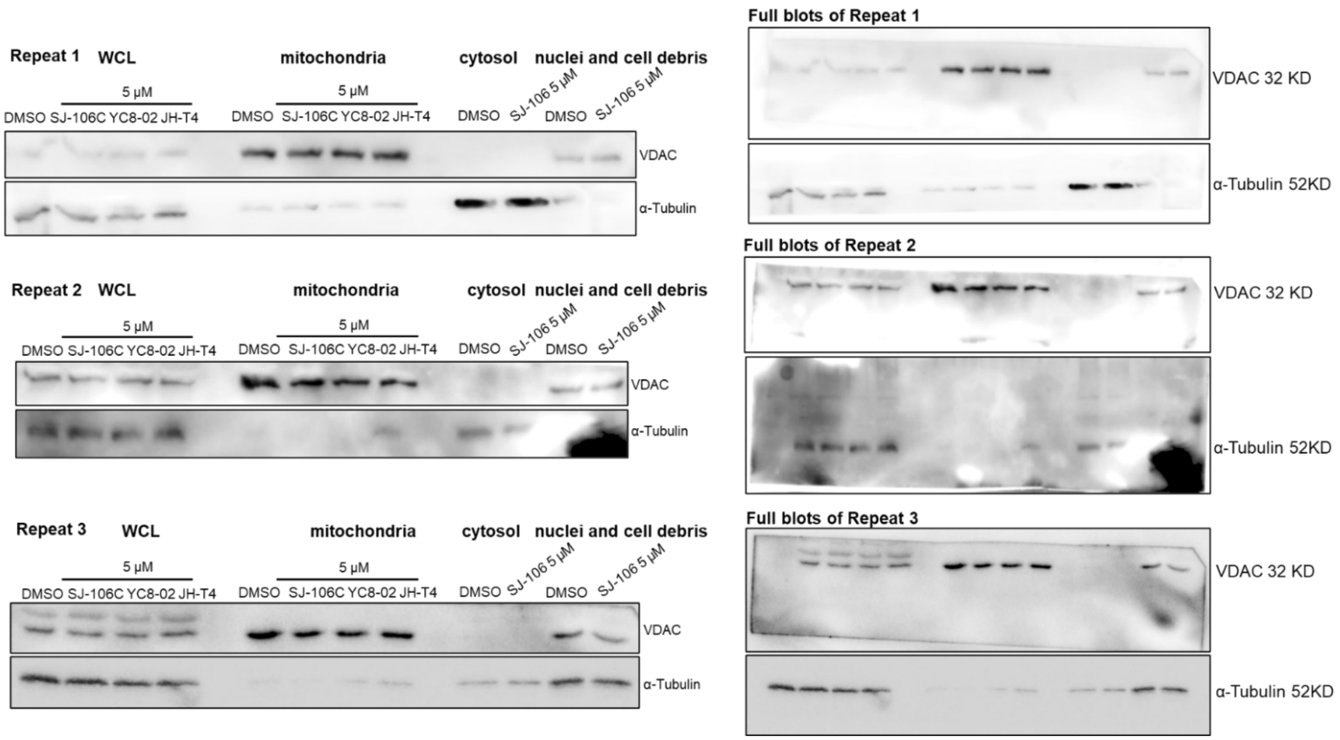

Figure S4. Karpas 422 cells were treated with SJ-106C, YC8-02, or JH-T4 at 5 μM for 6 hours. Whole cells and corresponding mitochondrial fractions were extracted for Western blot analysis using VDAC and α-Tubulin antibodies. The figures represent three independent Western blot experiments and corresponding full blots.

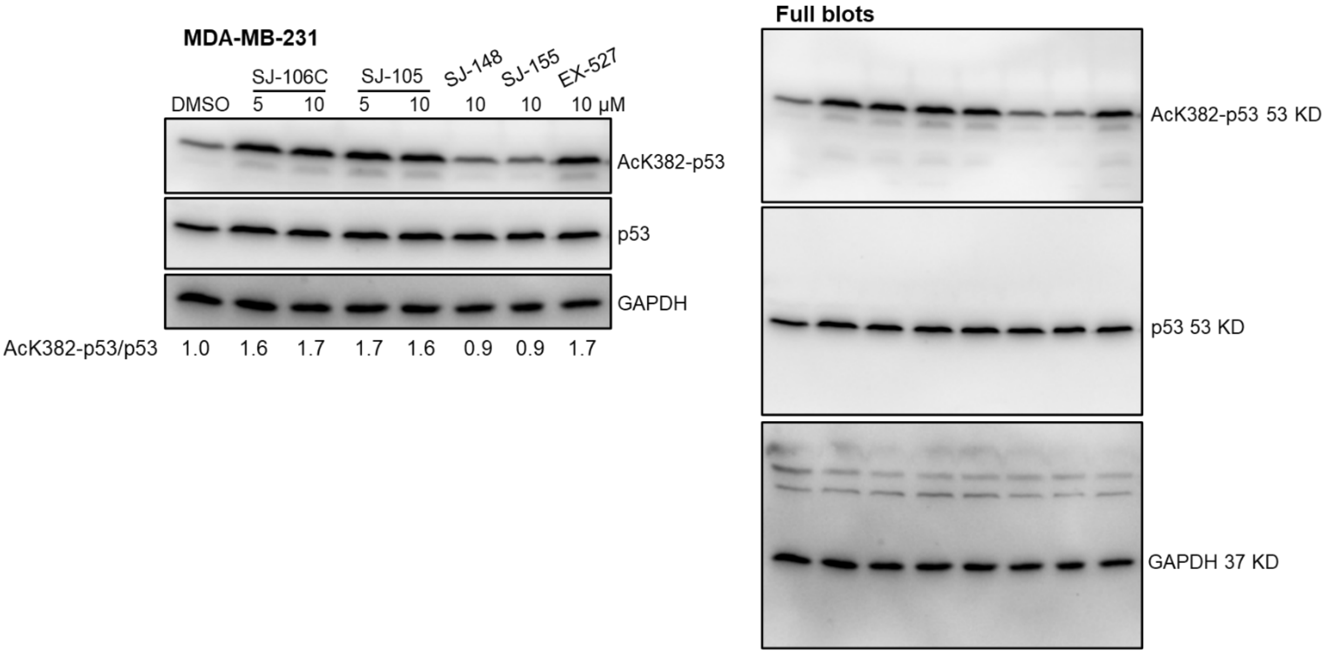

Figure S5. SJ-106C inhibits SIRT1 in MDA-MB-231 cells. Cells were treated with the indicated inhibitors at the indicated concentrations for 6 hours. The SIRT1 inhibitor EX527 was used as a positive control. The figures show representative Western blot and densitometry analysis and corresponding full blots.

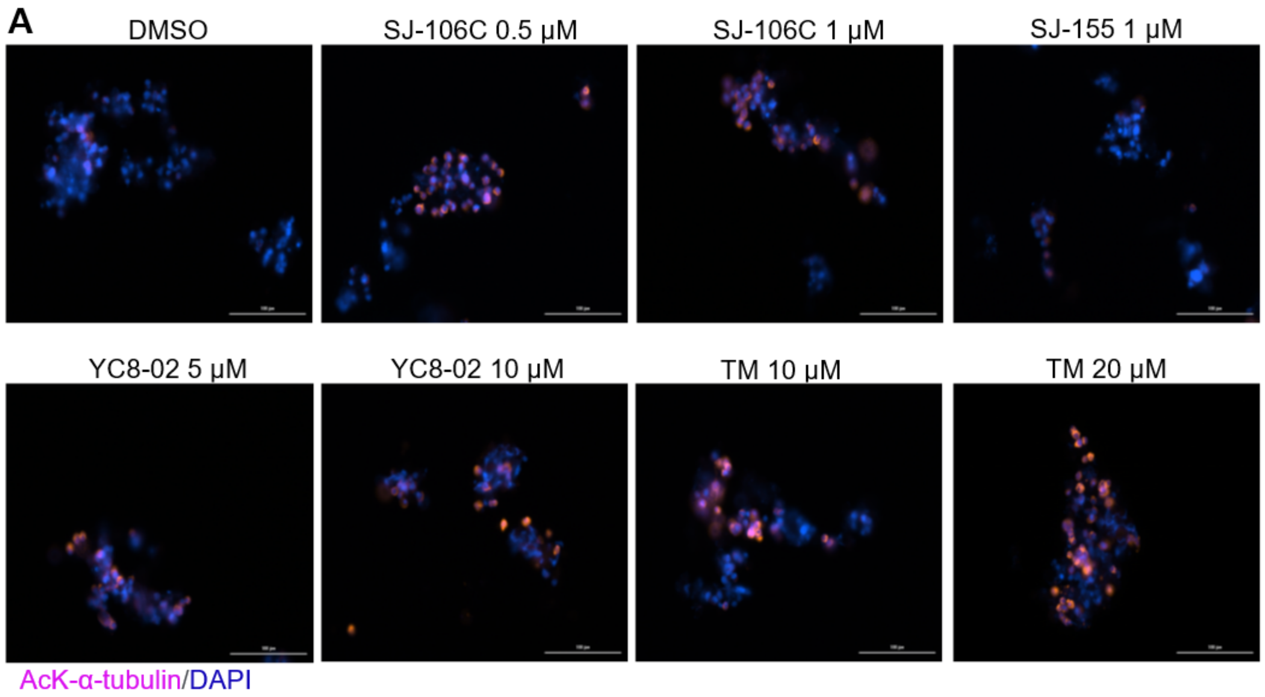

B

IC_{50} (μM)	SJ-106C	EX-527	TM
Karpas 422	0.65	44	41
OCI-LY7	0.45	29	16

Figure S6. SJ-106C inhibits SIRT2 in Karpas 422 cells. (**A**) Karpas 422 cells were treated with DMSO, SJ-106C, YC8-02, TM, or SJ-155 at indicated concentrations for 6 hours. The acetylation level of α-tubulin, a SIRT2 substrate, was detected using immunofluorescence, as previously described [20]. TM was used as a positive control. (**B**) Calculated IC_{50} values (μM) for 72 hours of SJ-106C, EX-527 or TM in DLBCL cells.

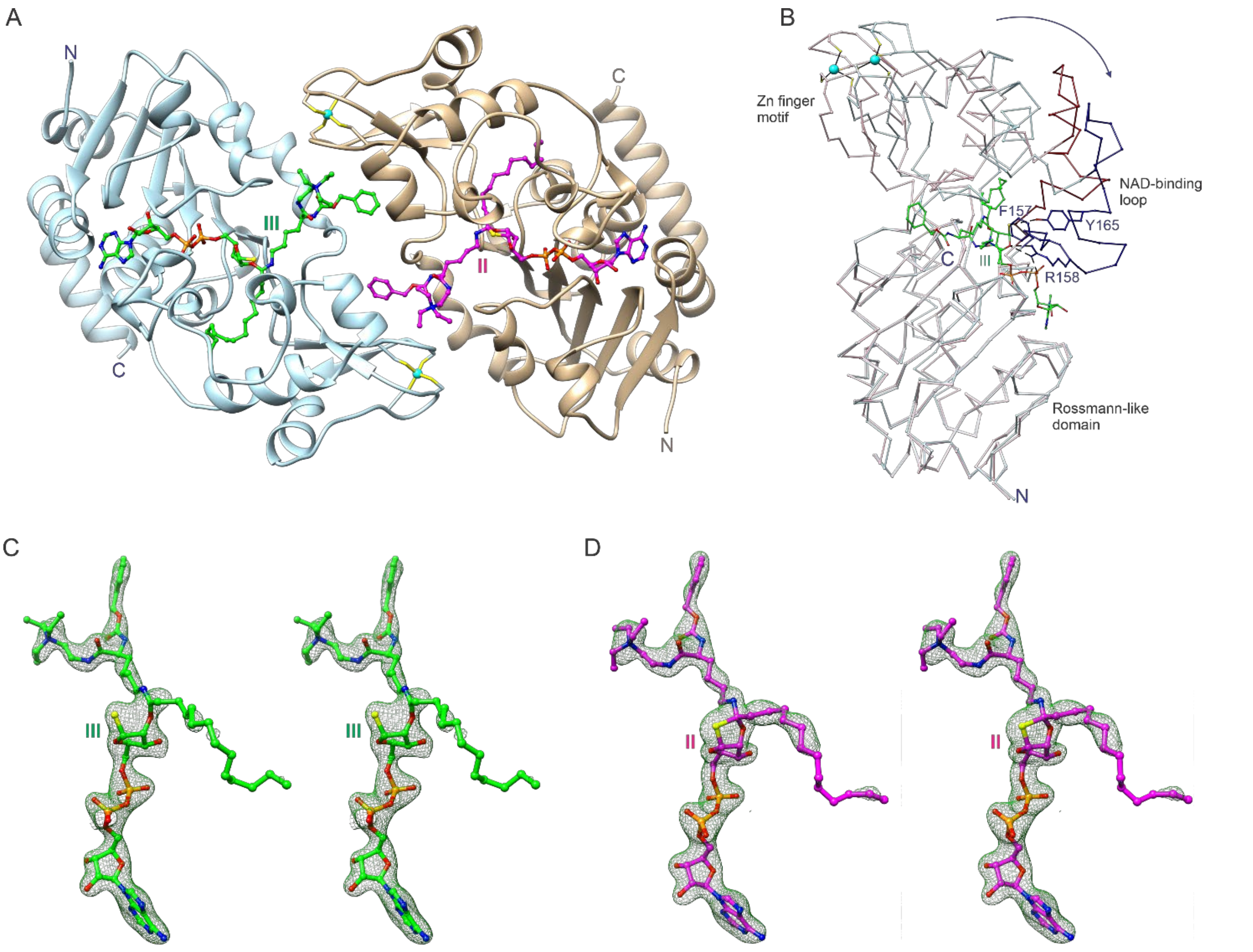

Figure S7. Crystal structure of SIRT3 bound to NH6-10-derived intermediates. (A) Asymmetric unit with SIRT3 shown as ribbons and intermediates II and III as balls-and-sticks. Chain A is colored light blue and chain B tan. (B) Intermediate induced domain closure of SIRT3. α-carbon traces of apo SIRT3 (pink and red; PDB code 3GLS) and intermediate III (green) bound SIRT3 (light and dark blue) are depicted in stick representation. (C) Stereo view of 2*F*o-*F*c composite omit electron density map around intermediate III contoured at RMSD of map. (D) Stereo view of 2*F*o-*F*c composite omit electron density map around intermediate II contoured at RMSD of map.

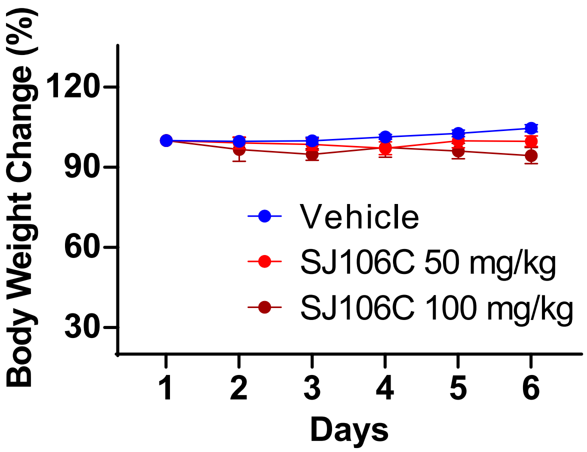

Figure S8. SJ-106C at 50 and 100 mg/kg once daily dosing for five days in NSG mice did not affect mouse body weights. n = 3 mice per group.

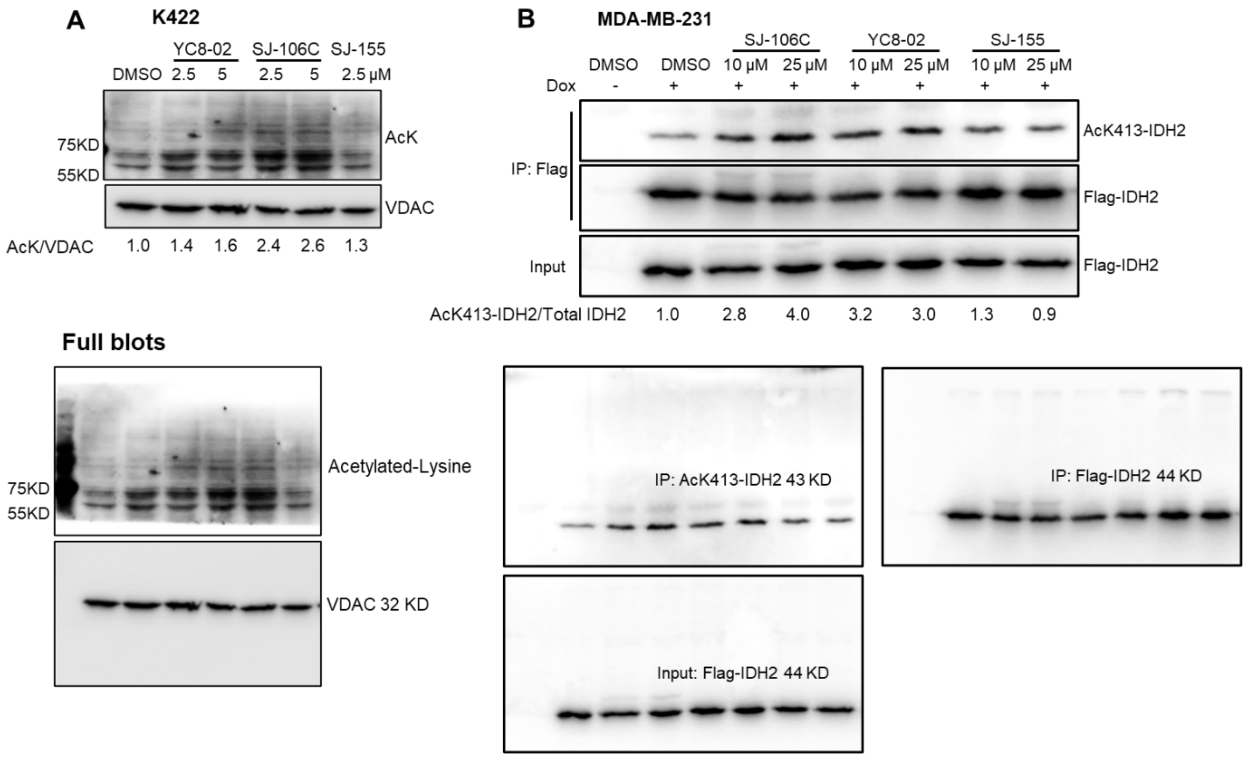

Figure S9. Representative Western blots in Figure 6 and the corresponding full blots. **(A)** Top (from Figure 6A): Western blot and densitometry analysis of mitochondrial acetylation from Karpas 422 cells treated with indicated compounds for 12 hours. Bottom: corresponding full blots. (**B**) Top (From Figure 6B): Western blot analysis of acetylated IDH2 (K413) after immunoprecipitation of Flag-tagged IDH2 in MDA-MB-231 cells treated with DMSO, SJ-106C, YC8-02, or SJ-155 at the indicated concentrations for 6 hours. Bottom: corresponding full blots.

Supplementary Table

Table S1. X-ray diffraction and structural refinement statistics.

**X-ray diffraction**	
Beamline	APS 24-ID-E
λ (Å)	0.97918 Å
Space group	$P2_1$
a, *b*, *c*, *β* (°)	39.43, 63.19, 114.61, 90.79
Resolution range (Å)	39.43-1.95
Resolution of highest shell (Å)	2.00 – 1.95
No. of reflections	119,067
Redundancy	3.0 (3.0)[a]
% Complete	97.4 (99.1)
$\langle I/\sigma_I \rangle$	5.3 (1.0)
CC(1/2)	0.991 (0.663)
R_{merge} (%)	10.0 (88.6)
**Structural refinement**	
No. of reflections	40,071
No. of reflections in working set	38,025
Resolution (Å)	1.95
RMSD bonds (Å)	0.004
RMSD angles (°)	0.701
R_{work}/R_{free} (%)	19.8/23.9
Ramachandran analysis	
Most favored (%)	91.0
Additional allowed (%)	9.0

[a]Values in parentheses are associated with the highest resolution shell.

General methods for synthesis

Unless otherwise stated, all reactions were carried out under air atmosphere. Commercially available reagents and solvents from Sigma Aldrich, TCI, Alfa-aesar, Combi-blocks, Chem-impex international, Ambeed, VWR, and Fisher scientific, USA were used as received. All isolated compounds were characterized by ^{1}H NMR and ^{13}C NMR spectra and recorded on Bruker 400MHz, and 500MHz spectrometers at Department of Chemistry and Chemical Biology, Cornell University. Chemical shifts (δ) are reported in ppm reference to the solvent peaks of CDCl3 (7.24 ppm) for ^{1}H NMR and 77.0 ppm for ^{13}C NMR, respectively. The following abbreviations were used to indicate multiplicity: s (Singlet), bs (broad Singlet), d (doublet), t (triplet), q (quartet), dd (double doublets), td (triplet of doublet), and m (multiplet). The mass spectral data were obtained on a ThermoFisher Scientific Exactive series DART Mass Spectrometer and LCMS. For column chromatography, silica gel (400–200 mesh) from VWR was used. A gradient elution using petroleum-ether/dichloromethane and ethyl acetate/methanol was performed, based on Merck aluminium TLC sheets (silica gel 60F254). All compounds were >95% pure by HPLC analysis.

Compound Characterization Data:

JH-T4[1] and YC8-02[2] were synthesized previously.

Scheme S1: Synthesis of *N*-thiomyristoyl lysine derivatives (NH series)

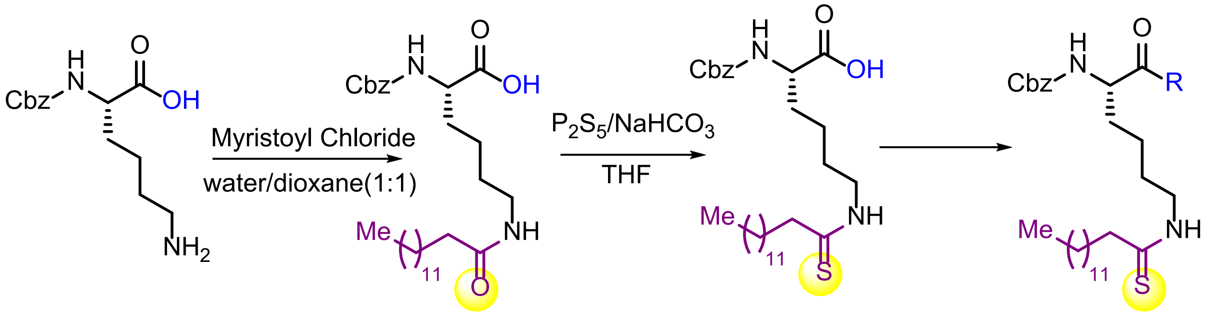

Compounds **S1** was synthesized previously. [3]

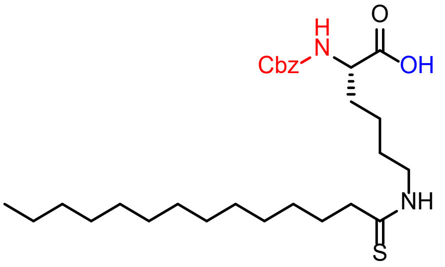

N2-((benzyloxy)carbonyl)-N6-tetradecanethioyl-L-lysine (**NH4-3);** NH4-3 was prepared using previously reported methods.[3] Eluent: Dichloromethane/methanol (9:1), (v/v). Physical State: colorless solid; Yield: 33%; ^{1}H NMR (500 MHz, CD$_3$OD) δ 7.40 – 7.33 (m, 4H), 7.31 (td, J = 6.1, 3.0 Hz, 1H),

5.15 – 5.08 (m, 2H), 4.18 (dd, *J* = 9.3, 4.7 Hz, 1H), 3.59 (t, *J* = 7.1 Hz, 2H), 2.60 (t, *J* = 7.6 Hz, 2H), 1.95 – 1.84 (m, 1H), 1.77 – 1.64 (m, 5H), 1.49 (ddd, *J* = 14.9, 11.0, 6.8 Hz, 2H), 1.31 (d, *J* = 11.2 Hz, 20H), 0.92 (t, *J* = 6.8 Hz, 3H). ^{13}C NMR (126 MHz, CD_3OD) δ 205.08, 174.50, 157.32, 136.82, 128.08, 127.59, 127.37, 66.23, 53.79, 45.69, 45.18, 31.70, 31.06, 29.43, 29.38, 29.34, 29.25, 29.11, 29.08, 28.56, 26.85, 23.02, 22.36, 13.09. HRMS (DART/Orbitrap) m/z: $[M+H]^+$ Calcd for $C_{28}H_{47}N_2O_4S$: 507.3251; Found 507.3253.

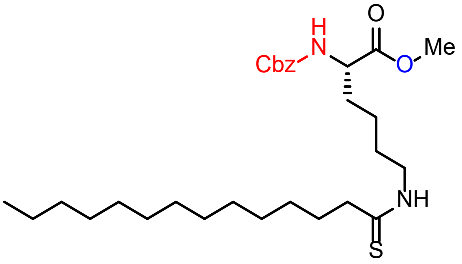

O-methyl N2-((benzyloxy)carbonyl)-N6-tetradecanethioyl-L-lysinate (NH4-1). The Compound NH4-1 was synthesized previously as NH4-8.[4] Eluent: Dichloromethane/methanol (9:1), (v/v). Physical State: colorless solid**;** Yield: 51%**;** ^{1}H NMR (500 MHz, $CDCl_3$) δ 7.74 (s, 1H), 7.35 (td, *J* = 8.8, 4.3 Hz, 5H), 5.50 (d, *J* = 8.3 Hz, 1H), 5.20 – 5.05 (m, 2H), 4.38 (td, *J* = 8.5, 4.5 Hz, 1H), 3.75 (s, 3H), 3.63 (tq, *J* = 13.1, 6.8 Hz, 2H), 2.63 (dd, *J* = 9.7, 5.8 Hz, 2H), 1.90 – 1.82 (m, 1H), 1.80 – 1.61 (m, 6H), 1.44 (p, *J* = 7.9 Hz, 2H), 1.35 – 1.21 (m, 21H), 0.88 (t, *J* = 6.9 Hz, 3H). ^{13}C NMR (126 MHz, $CDCl_3$) δ 205.78, 172.77, 156.29, 136.03, 128.59, 128.30, 128.00, 67.12, 53.28, 52.55, 47.08, 45.73, 32.70, 31.92, 29.68, 29.65, 29.63, 29.59, 29.54, 29.41, 29.36, 29.05, 26.96, 25.24, 24.66, 22.69, 14.14. HRMS (DART/Orbitrap) m/z: $[M+H]^+$ Calcd for $C_{29}H_{49}N_2O_4S$: 521.3408; Found 521.3415.

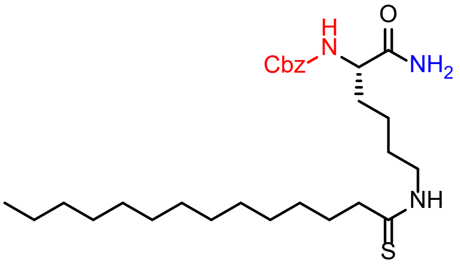

Benzyl (S)-(1-amino-1-oxo-6-tetradecanethioamidohexan-2-yl) carbamate (NH4-2). To a colorless solution of 253.4 mg (0.50 mmol) of compound **NH4-3** in 10 mL of THF were added at 0 ^{0}C 67 µL (0.70 mmol, 1.4 equiv.) of $ClCO_2Et$ and 209 µL (1.5 mmol, 3.0 equiv.) of Et3N. After stirring for 30 min at 0 0C, 0.75 ml of a 1.0 M aqueous solution of NH_4Cl (0.75 mmol,1.5 equiv.) was added at 0 ºC to the colorless suspension. The mixture was stirred for 30 min at 0 ºC, and 5 mL of water was added to the resulting mixture. The colorless clear solution was extracted with 30 mL of ethyl acetate and the aqueous layer was extracted with 20 mL of ethyl acetate. The organic layers were combined, washed with 5 mL of brine, and dried over anhydrous $MgSO_4$. The crude product was purified by silica gel column chromatography with dichloromethane and methanol to afford the compound NH4-2. Eluent: Dichloromethane/methanol (9:1), (v/v).; Physical State: colorless solid**.** Yield: 42%**;** ^{1}H NMR (500 MHz, CD_3OD) δ 7.40 – 7.34 (m, 4H), 7.33 – 7.29 (m, 1H), 5.13 (t, *J* = 11.1 Hz, 2H), 4.11 (dd, *J* = 9.2, 4.9 Hz, 1H), 3.59 (t, *J* = 7.2 Hz, 2H), 2.62 – 2.57 (m, 2H), 1.84 (ddt, *J* = 14.7, 10.6, 5.6 Hz, 1H), 1.77 – 1.64 (m,

5H), 1.52 – 1.40 (m, 2H), 1.31 (d, *J* = 10.6 Hz, 20H), 0.92 (t, *J* = 6.8 Hz, 3H). ^{13}C NMR (126 MHz, CD_3OD) δ 205.08, 176.24, 157.08, 136.77, 128.09, 127.63, 127.45, 66.32, 54.69, 45.68, 45.16, 31.68, 29.43, 29.41, 29.36, 29.32, 29.23, 29.08, 29.06, 28.56, 26.89, 22.98, 22.34, 13.05. HRMS (DART/Orbitrap) m/z: $[M+H]^+$ Calcd for $C_{28}H_{48}N_3O_3S$: 506.3411; Found 506.3420.

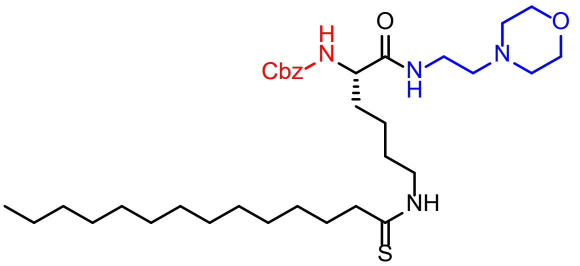

benzyl (S)-(1-((2-morpholinoethyl)amino)-1-oxo-6-tetradecanethioamidohexan-2-yl)carbamate (**NH4-9**). To a solution of N-Cbz thiomyristoyl lysine compound **NH4-3** (1 equiv. 0.5 mmol, 253.4 mg) in dichloromethane (10 mL) at room temperature was added N-methyl morpholine (NMM, 2.0 equiv., 1.0 mmol, 110 µL), followed by the addition of isobutyl chloroformate (1.05 equiv., 0.52 mmol, 68.5 µL) drop wise. The solution was stirred at room temperature for 1 h. Then, 2-morpholinoethan-1-amine (1.2 equiv., 0.6 mmol) was dissolved in dichloromethane and was poured to it. The resulting reaction mixture was stirred at room temperature for 18 h. The solvent was evaporated, and the residue was purified by silica gel column chromatography using CH_2Cl_2/MeOH (9:1) as elute to afford compound **NH4-9** as a colorless solid**.** Yield: 62%; ^{1}H NMR (500 MHz, $CDCl_3$) δ 7.84 (s, 1H), 7.39 – 7.29 (m, 5H), 6.78 (s, 1H), 5.67 (d, J = 8.0 Hz, 1H), 5.11 (s, 2H), 4.31 – 4.10 (m, 1H), 3.77 – 3.59 (m, 6H), 3.39 (dd, J = 11.3, 5.6 Hz, 2H), 2.67 – 2.60 (m, 2H), 2.53 (dd, J = 11.6, 5.8 Hz, 5H), 1.86 (d) J = 13.6, 7.8, 5.3 Hz, 1H), 1.77 – 1.67 (m, 4H), 1.49 – 1.40 (m, 2H), 1.34 – 1.20 (m, 22H), 0.89 (t, J = 6.9 Hz, 3H). ^{13}C NMR (126 MHz, $CDCl_3$) δ 205.80, 171.70, 156.39, 136.11, 128.60, 128.30, 127.97, 67.07, 66.53, 56.85, 54.53, 53.23, 47.13, 45.45, 35.53, 32.40, 31.92, 29.69, 29.66, 29.65, 29.58, 29.55, 29.42, 29.36, 29.08, 27.07, 22.69, 22.68, 14.14. HRMS (DART/Orbitrap) m/z: $[M+H]^+$ Calcd for $C_{34}H_{59}N_4O_4S$: 619.4252; Found 619.4258

Scheme S2: Synthesis of *N*-thiomyristoyl lysine ammonium derivatives.

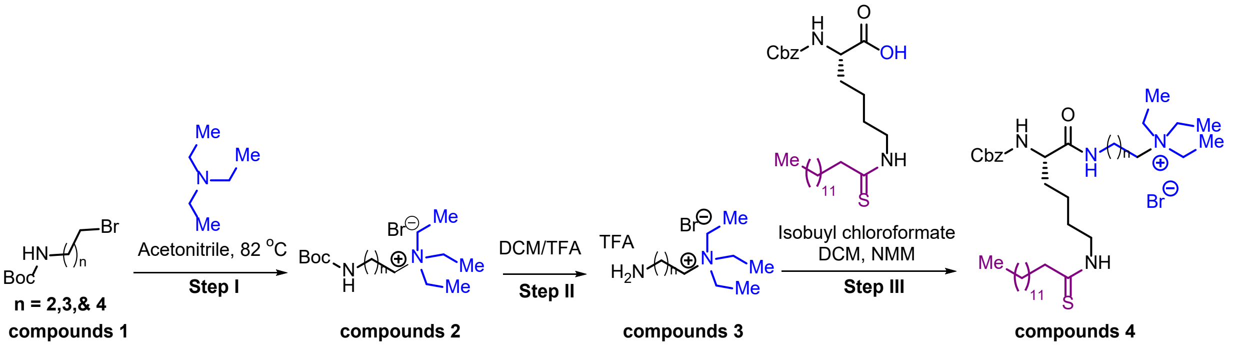

General synthetic procedure for Scheme S2:

Step I: *N*-Boc-bromo alkane (compounds **1**, 1.0 equiv. 1.0 mmol) was dissolved in dry acetonitrile (10 mL) at room temperature in a seal flask and triethylamine (3.0 equiv., 3.0 mmol) was added to this solution. The reaction was stirred at 82 °C for overnight (16 hours). The progress of the reaction was monitored by TLC and LC-MS. After completion of reaction, the solvent was removed using a rotavapor, and the residue was dried in high vacuum. The residue was washed by methyl *t*-butyl ether and separated by filtration. The solid powder was dried overnight at high vacuum and to offered desired product **2.**

Step II: The compounds **2** (1 equiv., 1.0 mmol), without further purification, was dissolved in 10 mL dichloromethane and 5 mL trifluoroacetic acid at room temperature and stirrer for 2 hours. The solvent was removed using a rotavapor, and the residue was re-dissolved in methanol and evaporated. This process was carried out 6-7 times and the residue was finally dried over high vacuum overnight. The compounds **3** obtained was used for next step without further purification.

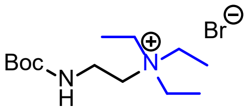

2-((tert-butoxycarbonyl)amino)-N,N,N-triethylethan-1-aminium bromide. (**NH4-P-104**). Physical State: colorless solid; Yield: 61%; ^{1}H NMR (500 MHz, DMSO) δ 7.14 (t, J = 5.9 Hz, 1H), 3.28 (q, J = 7.2 Hz, 8H), 3.15 (t, 3H), 1.40 (s, 9H), 1.19 (t, J = 7.2 Hz, 9H).
^{13}C NMR (126 MHz, DMSO) δ 156.15, 79.08, 54.55, 52.78, 47.20, 33.78, 28.61, 7.52. HRMS (DART/Orbitrap) m/z: [M]$^+$ Calcd for $C_{13}H_{29}N_2O_2$: 245.2224; Found 245.2231.

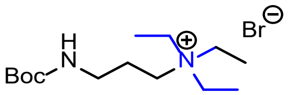

3-((tert-butoxycarbonyl)amino)-N,N,N-triethylpropan-1-aminium bromide (**SJ-161**) Physical State: colorless solid; Yield: 59%; ^{1}H NMR (500 MHz, CDCl3) δ 5.93 (s, 1H), 3.38 (p, J = 8.9 Hz, 7H), 3.29 – 3.17 (m, 2H), 3.15 – 3.10 (m, 1H), 1.36 (d, J = 15.5 Hz, 9H), 1.32 (d, J = 6.8 Hz, 9H). ^{13}C NMR (126 MHz, $CDCl_3$) δ 156.43, 79.20, 55.90, 53.50, 46.25, 37.28, 28.38, 22.65, 8.80, 7.99. HRMS (DART/Orbitrap) m/z: [M]$^+$ Calcd for $C_{14}H_{31}N_2O_2$: 259.2385; Found 259.2386.

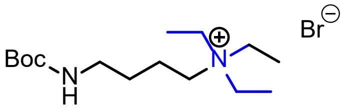

4-((tert-butoxycarbonyl)amino)-N,N,N-triethylbutan-1-aminium bromide (SJ-152). Physical State: colorless solid; Yield: 62%; ^{1}H NMR (500 MHz, CD_3OD) δ 3.38 – 3.34 (m, 4H), 3.27 – 3.22 (m, 4H), 3.13 (t, *J* = 6.7 Hz, 2H), 1.77 – 1.68 (m, 2H), 1.57 (p, *J* = 7.1 Hz, 2H), 1.46 (s, 9H), 1.33 (t, 9H). ^{13}C NMR (126 MHz, CD_3OD) δ 157.25, 78.69, 56.38, 52.52, 46.55, 38.92, 27.36, 26.45, 18.49, 7.86, 6.33. HRMS (DART/Orbitrap) m/z: [M]$^+$ Calcd for $C_{15}H_{33}N_2O_2$: 273.2536; Found 273.2537.

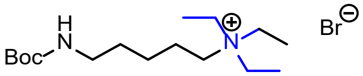

5-((tert-butoxycarbonyl)amino)-N,N,N-triethylpentan-1-aminium bromide (SJ-167). Physical State: colorless solid; Yield: 58%; ^{1}H NMR (500 MHz, $CDCl_3$) δ 4.94 (s, 1H), 3.43 (q, J = 7.3 Hz, 6H), 3.27 (t, J = 8.6 Hz, 2H), 3.06 (t, J = 6.7 Hz, 2H), 1.72 (dq, J = 16.1, 7.3 Hz, 2H), 1.52 (d, J = 7.3 Hz, 2H), 1.35 (dq, J = 14.6, 7.2 Hz, 20H). ^{13}C NMR (126 MHz, CDCl3) δ 156.20, 78.92, 57.49, 53.48, 39.51, 28.40, 23.29, 21.44, 8.08. HRMS (DART/Orbitrap) m/z: $[M]^+$ Calcd for $C_{16}H_{35}N_2O_2$: 287.2708; Found 287.2699.

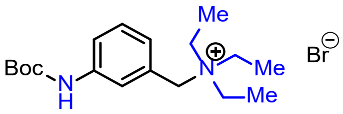

***N*-(3-((tert-butoxycarbonyl)amino)benzyl)-*N,N*-diethylethanaminium bromide** (**SJ-214**). Physical State: colorless solid; Yield: 52%; ^{1}H NMR (500 MHz, CDCl3) δ 7.85 (s, 1H), 7.77 (s, 1H), 7.71 (dd, J = 8.2, 2.1 Hz, 1H), 7.30 (t, 1H), 7.12 (d, J = 7.6 Hz, 1H), 4.62 (s, 2H), 3.38 (q, J = 7.2 Hz, 6H), 1.51 (s, 9H), 1.45 (t, J = 7.2 Hz, 9H). ^{13}C NMR (126 MHz, CDCl3) δ 153.12, 140.11, 129.82, 127.27, 126.06, 122.70, 120.85, 80.61, 61.34, 53.15, 28.34, 8.55. HRMS (DART/Orbitrap) m/z: $[M]^+$ Calcd for C18H31N2O2: 307.2386; Found 307.2387.

Step III: *N*-Cbz thiomyristoyl lysine was prepared previously.[3] To a solution of *N*-Cbz thiomyristoyl lysine (1 equiv. 0.5 mmol, 253.4 mg) in dichloromethane (10 mL) at room temperature was added *N*-methyl morpholine (NMM, 2.0 equiv., 1.0 mmol, 110 µL), followed by the addition of isobutyl chloroformate (1.05 equiv., 0.52 mmol, 68.5 µL) drop wise. The solution was stirred at room temperature for 1 h. Then, compounds **3** (1.2 equiv., 0.6 mmol) was dissolved in dichloromethane and was poured to it. The resulting reaction mixture was stirred at room temperature for 18 h. The solvent was evaporated, and the residue was purified by silica gel column chromatography using CH_2Cl_2/MeOH (9:1) as elute to afford compounds **4**.

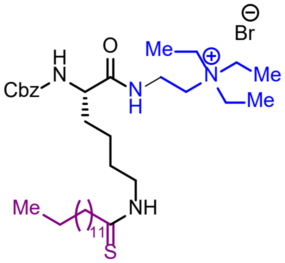

(S)-2-(2-(((benzyloxy)carbonyl)amino)-6-butanethioamidohexanamido)-N,N,N-triethylethan-1-aminium bromide (**NH6-10**). Eluent: Dichloromethane/methanol (9:1), (v/v). Physical State: colorless semi-solid Yield: 59%; ^{1}H NMR (500 MHz, CD_3OD) δ 7.40 – 7.31 (m, 5H), 5.15 – 5.08 (m, 2H), 4.03 (dd, J = 9.2, 5.2 Hz, 1H), 3.64 – 3.53 (m, 4H), 3.38 (t, J = 7.2 Hz, 6H), 3.30 (t, J = 7.1 Hz, 2H), 2.62 – 2.57 (m, 2H), 1.90 – 1.76 (m, 2H), 1.76 – 1.65 (m, 5H), 1.53 – 1.39 (m, 3H), 1.37 – 1.25 (m, 27H), 0.92 (t, J = 6.9 Hz, 3H). ^{13}C NMR (126 MHz, CD_3OD) δ 205.03, 173.88, 157.03, 136.87, 128.18, 127.66, 127.29, 66.23, 55.47, 54.52, 51.42, 46.64, 45.74, 45.09, 37.79, 31.71, 31.46, 29.49, 29.45, 29.41, 29.37, 29.28, 29.12, 28.60, 26.95, 26.20, 23.09, 22.38, 20.74, 13.16. HRMS (DART/Orbitrap) m/z: $[M]^+$ Calcd for $C_{36}H_{65}N_4O_3S^+$: 633.4772; Found 6334771. TLC: R_f = 0.45 dichloromethane/methanol (9/1, v/v).

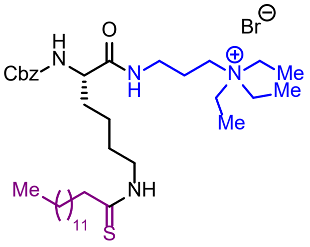

(S)-3-(2-(((benzyloxy)carbonyl)amino)-6-tetradecanethioamidohexanamido)-N,N,N-triethylpropan-1-aminiumbromide (**SJ-163**). Eluent: Dichloromethane/methanol (9:1, v/v). Physical State: colorless semi-solid Yield: 49% 1H NMR (500 MHz, CD_3OD) δ 7.41 – 7.29 (m, 5H), 5.12 (s, 2H), 4.00 (dd, J = 8.9, 5.3 Hz, 1H), 3.61 (t, J = 7.2 Hz, 2H), 3.47 – 3.37 (m, 1H), 3.33 (p, J = 1.6 Hz, 1H), 3.28 (t, J = 7.3 Hz, 6H), 3.24 – 3.11 (m, 2H), 2.61 (t, J = 7.6 Hz, 2H), 1.92 (dq, J = 15.7, 7.6 Hz, 2H), 1.86 – 1.78 (m, 1H), 1.78 – 1.65 (m, 5H), 1.56 – 1.39 (m, 2H), 1.29 (d, J = 9.1 Hz, 29H), 0.92 (t, J = 6.8 Hz, 3H).^{13}C NMR (126 MHz, CD_3OD) δ 205.06, 174.30, 157.08, 136.97, 128.17, 127.60, 127.13, 66.11, 55.66, 54.76, 52.71, 45.72, 45.06, 35.71, 31.70, 31.15, 29.47, 29.43, 29.39, 29.36, 29.27, 29.11, 29.10, 28.60, 26.95, 23.10, 22.37, 21.77, 13.12, 6.41. HRMS (DART/Orbitrap) m/z: $[M]^+$ Calcd for $C_{37}H_{67}N_4O_3S^+$: 648.5006; Found 648.4991. TLC: R_f = 0.45 dichloromethane/methanol (9/1, v/v).

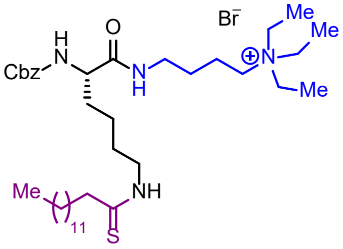

(S)-4-(2-(((benzyloxy)carbonyl)amino)-6-tetradecanethioamidohexanamido)-*N,N,N*-triethylbutan-1-aminiumbromide (**SJ-106C**) Eluent: Dichloromethane/methanol (9:1, v/v). Physical State: colorless semi-solid; Yield: 54%. ^{1}H NMR (500 MHz, CD_3OD) δ 7.41 – 7.34 (m, 5H), 7.32 (s, 1H), 5.12 (s, 2H), 4.06 – 4.00 (m, 1H), 3.61 (t, *J* = 7.2 Hz, 2H), 3.23 – 3.19 (m, 2H), 2.61 (t, *J* = 7.6 Hz, 2H), 1.77 – 1.65 (m, 8H), 1.64 – 1.60 (m, 2H), 1.31 (d, *J* = 7.2 Hz, 39H), 0.92 (t, *J* = 7.0 Hz, 3H). ^{13}C NMR (126 MHz, CD_3OD) δ 205.09, 173.95, 157.03, 136.98, 128.17, 127.61, 127.16, 66.12, 56.46, 55.53, 52.61, 51.97, 45.72, 45.05, 37.73, 31.69, 31.34, 29.45, 29.41, 29.37, 29.34, 29.24, 29.09, 28.57, 26.94, 26.03, 23.06, 22.35, 18.50, 13.08. HRMS (DART/Orbitrap) m/z: $[M]^+$ Calcd for $C_{38}H_{70}N_4O_3S$: 662.5163; Found 662.5148. TLC: R_f = 0.45 dichloromethane/methanol (9/1, v/v).

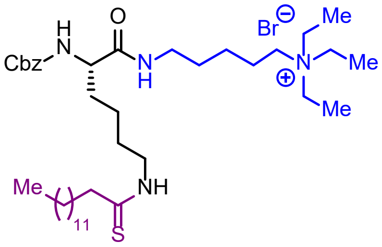

(S)-5-(2-(((benzyloxy)carbonyl)amino)-6-tetradecanethioamidohexanamido)-*N,N,N*-triethylpentan-1-aminiumbromide (**SJ-172**) Eluent: Dichloromethane/methanol (9:1, v/v). Physical

State: colorless semi-solid. Yield: 52%. ^{1}H NMR (500 MHz, $CDCl_3$) δ 9.75 (s, 1H), 8.39 (s, 1H), 7.38 – 7.28 (m, 5H), 6.44 (d, J = 8.3 Hz, 1H), 5.06 (d, J = 2.3 Hz, 2H), 4.30 (td, J = 8.3, 5.1 Hz, 1H), 3.71 – 3.58 (m, J = 6.5 Hz, 2H), 3.30 (m, J = 7.7 Hz, 10H), 2.73 (t, J = 7.7 Hz, 2H), 1.91 (dq, J = 13.9, 6.7 Hz, 1H), 1.76 (tq, J = 14.7, 7.3 Hz, 7H), 1.63 (q, J = 6.6 Hz, 2H), 1.46 (dq, J = 16.3, 8.1 Hz, 4H), 1.36 – 1.19 (m, 29H), 0.88 (t, J = 6.9 Hz, 3H). ^{13}C NMR (126 MHz, $CDCl_3$) δ 205.12, 172.80, 156.34, 136.86, 128.48, 127.91, 127.56, 66.36, 57.75, 55.05, 53.21, 46.39, 45.14, 38.06, 32.49, 31.92, 29.93, 29.72, 29.66, 29.64, 29.59, 29.36, 29.17, 27.86, 26.29, 23.26, 22.74, 22.69, 21.15, 14.14, 7.83. HRMS (DART/Orbitrap) m/z: $[M]^+$ Calcd for $C_{39}H_{71}N_4O_3S$: 676.5319; Found 676.5304. TLC: Rf = 0.45 dichloromethane/methanol (9/1, v/v).

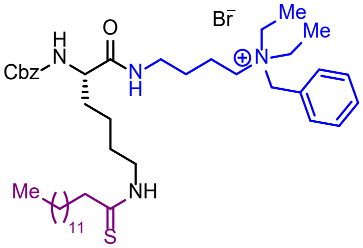

(S)-N-benzyl-4-(2-(((benzyloxy)carbonyl)amino)-6-tetradecanethioamidohexanamido)-N,N-diethylbutan-1-aminiumbromide (SJ-174). Eluent: Dichloromethane/methanol (9:1, v/v). Physical State: colorless semi-solid. Yield: 47%. ^{1}H NMR (500 MHz, CDCl3) 1H NMR (500 MHz, CD_3OD) δ 8.21 (t, J = 6.0 Hz, 1H), 7.55 (s, 5H), 7.37 – 7.28 (m, 5H), 5.06 (d, J = 4.8 Hz, 2H), 4.51 (d, J = 5.5 Hz, 1H), 3.61 (t, J = 7.2 Hz, 2H), 3.37 (s, 4H), 3.26 (d, J = 7.3 Hz, 6H), 3.15 (d, J = 8.4 Hz, 3H), 2.61 (t, J = 7.6 Hz, 2H), 1.92 – 1.79 (m, 3H), 1.72 (dt, J = 14.9, 6.8 Hz, 4H), 1.60 (s, 2H), 1.52 – 1.47 (m, 1H), 1.42 (td, J = 7.3, 3.3 Hz, 6H), 1.37 – 1.26 (m, 21H), 0.92 (t, J = 6.8 Hz, 3H). 13C NMR (126 MHz, CD_3OD) δ 205.04, 174.15, 157.04, 136.94, 132.32, 130.47, 129.16, 128.14, 127.58, 127.39, 127.11, 66.07, 60.54, 56.68, 55.62, 52.80, 48.48, 45.69, 45.04, 37.97, 37.84, 31.68, 31.32, 29.45, 29.40, 29.36, 29.33, 29.24, 29.08, 28.56, 26.93, 25.91, 23.07, 22.34, 18.86, 13.08, 6.85. HRMS (DART/Orbitrap) m/z: $[M]^+$ Calcd for $C_{43}H_{71}N_4O_3S$: 724.5319; Found 724.5310. TLC: Rf = 0.45 dichloromethane/methanol (9/1, v/v).

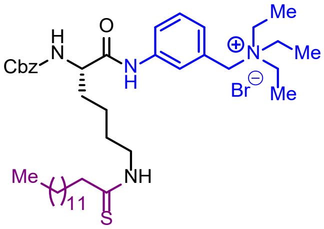

(S)-N-(3-(2-(((benzyloxy)carbonyl)amino)-6-tetradecanethioamidohexanamido)benzyl)-N,N-diethylethanaminium (SJ-218)

Eluent: Dichloromethane/methanol (9:1, v/v). Physical State: colorless semi-solid

Yield: 72%. ^{1}H NMR (500 MHz, CD_3OD) δ 7.96 (s, 1H), 7.65 (d, *J* = 8.2 Hz, 1H), 7.48 (t, *J* = 7.9 Hz, 1H), 7.40 – 7.32 (m, 4H), 7.29 (d, *J* = 8.0 Hz, 2H), 5.17 – 5.06 (m, 2H), 4.46 (s, 2H), 4.28 (dd, *J* = 8.9, 5.4 Hz, 1H), 3.61 (t, *J* = 7.2 Hz, 2H), 3.28 (q, *J* = 7.2 Hz, 6H), 2.59 (t, *J* = 7.6 Hz, 2H), 1.91 (ddt, *J* = 15.4, 11.2, 5.9 Hz, 1H), 1.80 (dq, *J* = 9.3, 4.4 Hz, 1H), 1.71 (t, *J* = 7.8 Hz, 4H), 1.55 (dq, *J* = 11.9, 7.2 Hz, 1H), 1.44 (t, *J* = 7.2 Hz, 9H), 1.30 (s, 21H), 0.91 (t, *J* = 6.8 Hz, 3H). ^{13}C NMR (126 MHz, CD_3OD) δ 205.05, 172.36, 157.14, 139.18, 136.76, 129.55, 128.12, 127.82, 127.72, 127.66, 127.43, 123.74, 121.84, 66.34,

63.73, 59.75, 55.83, 53.22, 52.26, 45.71, 45.06, 31.70, 29.45, 29.43, 29.39, 29.35, 29.26, 29.10, 28.58, 27.00, 23.02, 22.36, 13.12, 6.73. HRMS (DART/Orbitrap) m/z: $[M]^+$ Calcd for $C_{41}H_{67}N_4O_3S$: 696.5006; Found 696.4998. TLC: Rf = 0.5 dichloromethane/methanol (9/1, v/v).

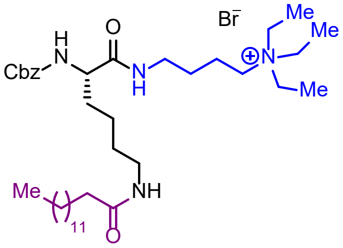

(S)-4-(2-(((benzyloxy)carbonyl)amino)-6-tetradecanamidohexanamido)-N,N,N-triethylbutan-1-aminium SJ-155. To a solution of N^2-((benzyloxy)carbonyl)-N^6-tetradecanoyl-L-lysine **compound S1**[3] (1 equiv. 0.5 mmol, 245.4 mg) in dichloromethane (10 mL) at room temperature was added *N*-methyl morpholine (NMM, 2.0 equiv., 1.0 mmol, 110 µL), followed by the addition of isobutyl chloroformate (1.05 equiv., 0.52 mmol, 68.5 µL) drop wise. The solution was stirred at room temperature for 1 h. Then, compounds 3.TFA salt (SJ-152) (1.2 equiv., 0.6 mmol) was dissolved in dichloromethane and was poured to it. The resulting reaction mixture was stirred at room temperature for 18 h. The solvent was evaporated, and the residue was purified by silica gel column chromatography using CH_2Cl_2/MeOH (9:1) as elute to afford compounds **SJ-155**. Eluent: Dichloromethane/methanol (9:1), (v/v). Physical State: colorless solid; Yield: 52%; ^{1}H NMR (500 MHz, $CDCl_3$) δ 8.48 (s, 1H), 7.40 – 7.20 (m, 5H), 6.92 (s, 1H), 6.50 (d, J = 8.1 Hz, 1H), 5.06 (s, 2H), 4.24 (td, J = 8.5, 5.1 Hz, 1H), 3.51 (s, 2H), 3.38 (dt, J = 12.1, 6.1 Hz, 1H), 3.28 (p, J = 6.6 Hz, 8H), 3.19 (q, J = 6.5 Hz, 2H), 2.28 – 2.12 (m, 2H), 1.90 – 1.69 (m, 4H), 1.64 (t, J = 6.5 Hz, 2H), 1.61 – 1.49 (m, 4H), 1.42 (dt, J = 13.6, 6.5 Hz, 2H), 1.34 – 1.20 (m, 27H), 0.87 (t, J = 6.9 Hz, 3H). ^{13}C NMR (126 MHz, $CDCl_3$) δ 173.95, 173.30, 156.41, 136.89, 128.48, 127.89, 127.70, 127.53, 66.34, 57.40, 55.45, 53.10, 38.58, 37.91, 36.62, 32.19, 31.91, 29.70, 29.68, 29.65, 29.58, 29.49, 29.42, 29.35, 28.49, 25.98, 25.96, 22.68, 19.23, 14.13, 7.74, 7.71. HRMS (DART/Orbitrap) m/z: $[M]^+$ Calcd for $C_{38}H_{69}N_4O_4$: 645.5316; Found 645.5313.; TLC: Rf = 0.5 dichloromethane/methanol (9/1, v/v).

Scheme S3: Synthesis of *N*-thiomyristoyl lysine amine derivatives

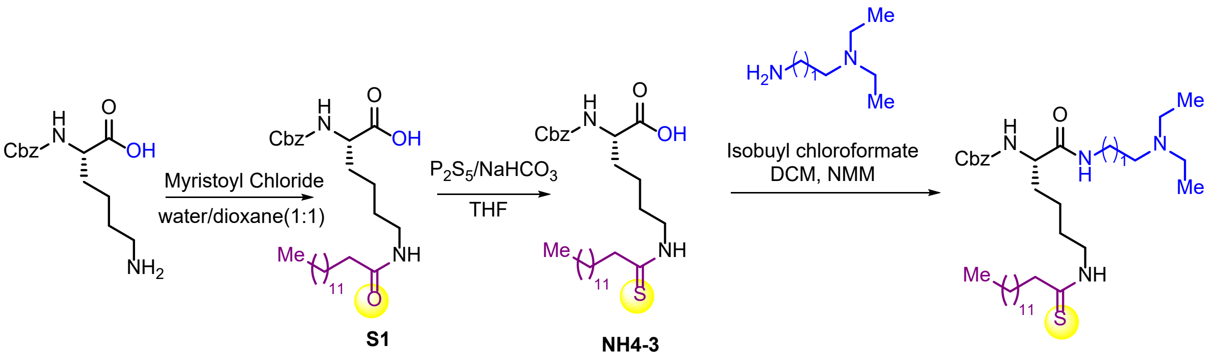

General procedure for Scheme S3: To a solution of *N*-Cbz thiomyristoyl lysine (NH4-3)[3] (1 equiv. 0.5 mmol, 253.4 mg) in dichloromethane (10 mL) at room temperature was added *N*-methyl morpholine

(NMM, 2.0 equiv., 1.0 mmol, 110 µL), followed by the addition of isobutyl chloroformate (1.05 equiv., 0.52 mmol, 68.5 µL) drop wise. The solution was stirred at room temperature for 1 h. Then, corresponding amine (1.2 equiv., 0.6 mmol) was dissolved in dichloromethane and was poured to it. The resulting reaction mixture was stirred at room temperature for 18 h. The solvent was evaporated, and the residue was purified by silica gel column chromatography using CH_2Cl_2/MeOH (9:1) as elute to afford corresponding amides derivatives.

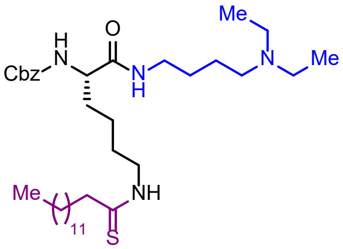

Benzyl (S)-(1-((4-(diethylamino)butyl)amino)-1-oxo-6-tetradecanethioamidohexan-2-yl)carbamate (SJ-105). Eluent: dichloromethane/methanol (9:1, v/v). Physical State: colorless solid; Yield: 75%. ^{1}H NMR (500 MHz, CD_3OD) δ 7.41 – 7.35 (m, 5H), 7.32 (td, J = 6.1, 2.7 Hz, 1H), 5.12 (s, 2H), 4.05 (dd, J = 9.1, 5.2 Hz, 1H), 3.60 (t, J = 7.2 Hz, 2H), 3.22 – 3.21 (m, 2H), 3.14 (dd, J = 10.5, 6.2 Hz, 2H), 2.64 – 2.60 (m, 2H), 1.77 – 1.68 (m, 8H), 1.62 (t, J = 7.1 Hz, 2H), 1.41 (dd, J = 7.0, 2.0 Hz, 2H), 1.35 – 1.30 (m, 30H), 0.91 (t, J = 7.1 Hz, 3H). ^{13}C NMR (126 MHz, CD_3OD) δ 205.03, 173.88, 157.03, 136.87, 128.18, 127.66, 127.29, 66.23, 55.47, 54.52, 51.42, 46.64, 45.74, 45.09, 37.79, 31.46, 29.49, 29.45, 29.41, 29.37, 29.28, 29.12, 28.60, 26.95, 26.20, 23.09, 22.38, 20.74, 13.16. HRMS (DART/Orbitrap) m/z: $[M+H]^+$ Calcd for $C_{36}H_{65}N_4O_3S$: 633.4772; Found 633.4777. TLC: R_f = 0.45 dichloromethane/methanol (9/1, v/v).

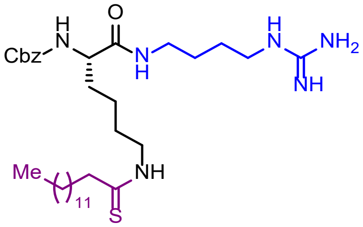

Benzyl (S)-(1-((4-guanidinobutyl)amino)-1-oxo-6-tetradecanethioamidohexan-2-yl)carbamate (SJ-184). Eluent: Dichloromethane/methanol (9:1), (v/v); Physical State: colorless semi-solid; Yield: 53%; ^{1}H NMR (500 MHz, CD_3OD) δ 7.40 – 7.29 (m, 5H), 5.15 – 5.08 (m, 2H), 4.64 (s, 2H), 4.05 (dd, J = 9.0, 5.3 Hz, 1H), 3.91 (s, 2H), 3.60 (t, J = 7.1 Hz, 2H), 3.26 – 3.17 (m, 6H), 2.86 (s, 2H), 2.60 (t, J = 7.6 Hz, 2H), 1.79 (d, J = 8.5 Hz, 1H), 1.73 (t, J = 7.3 Hz, 2H), 1.68 (dt, J = 9.0, 4.5 Hz, 2H), 1.62 – 1.57 (m, 3H), 1.50 – 1.40 (m, 2H), 1.31 (d, J = 10.1 Hz, 20H), 0.92 (t, J = 6.8 Hz, 3H). ^{13}C NMR (126 MHz, CD_3OD) δ 205.09, 173.78, 157.17, 157.09, 136.76, 128.12, 127.67, 127.40, 66.32, 63.99, 55.31, 53.42, 45.69, 45.09, 42.83, 40.71, 38.19, 31.68, 29.44, 29.40, 29.36, 29.32, 29.23, 29.07, 28.55, 26.90, 26.20, 25.63, 23.01, 22.34, 13.06. HRMS (DART/Orbitrap) m/z: $[M+H]^+$ Calcd for $C_{33}H_{59}N_6O_3S$: 619.4364; Found 619.4362. TLC: Rf = 0.45 dichloromethane/methanol, (8/2).

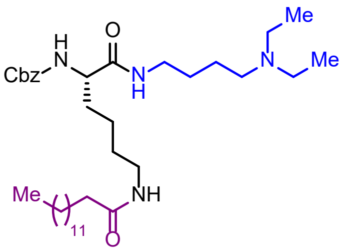

benzyl (S)-(1-((4-(diethylamino)butyl)amino)-1-oxo-6-tetradecanethioamidohexan-2-yl)carbamate SJ-148; N^2-((benzyloxy)carbonyl)-N^6-tetradecanoyl-L-lysine **compound S1**[3] (1 equiv. 0.5 mmol, 245.4 mg) in dichloromethane (10 mL) at room temperature was added *N*-methyl morpholine (NMM, 2.0 equiv., 1.0 mmol, 110 µL), followed by the addition of isobutyl chloroformate (1.05 equiv., 0.52 mmol, 68.5 uL) drop wise. The solution was stirred at room temperature for 1 h. Then, *N,N*-diethylbutane-1,4-diamine (1.2 equiv., 0.6 mmol) was dissolved in dichloromethane and was poured into it. The resulting reaction mixture was stirred at room temperature for 18 h. The solvent was evaporated, and the residue was purified by silica gel column chromatography using CH_2Cl_2/MeOH (9:1) as elute to afford corresponding amides derivative SJ-148. Eluent: dichloromethane/methanol (9:1), (v/v). Physical State: colorless solid**;** Yield: 72%; ^{1}H NMR (500 MHz, CD_3OD) δ 7.40 – 7.30 (m, 5H), 5.15 – 5.06 (m, 2H), 4.02 (dd, J = 9.1, 5.1 Hz, 1H), 3.51 (q, J = 7.3 Hz, 1H), 3.30 – 3.21 (m, 2H), 3.17 (q, J = 6.5 Hz, 2H), 3.10-3.06 (m, 8H), 3.02 (q, J = 7.3 Hz, 3H), 2.97 – 2.91 (m, 2H), 2.18 (t, J = 7.5 Hz, 2H), 1.78 (s, 1H), 1.69 – 1.64 (m, 2H), 1.60 (q, J = 7.5 Hz, 3H), 1.53 (dd, J = 5.6, 2.1 Hz, 2H), 1.38 (t, J = 7.3 Hz, 2H), 1.31 – 1.27 (m, 22H), 0.92 (t, J = 6.8 Hz, 3H). ^{13}C NMR (126 MHz, CD_3OD) δ 174.88, 173.83, 157.02, 136.88, 128.13, 127.64, 127.48, 127.31, 66.17, 55.45, 52.25, 51.48, 46.80, 46.27, 38.54, 38.00, 35.81, 31.69, 29.41, 29.38, 29.35, 29.25, 29.09, 29.06, 28.95, 28.66, 26.45, 25.72, 22.95, 22.35, 21.25, 13.08, 8.47, 8.28, 6.30. HRMS (DART/Orbitrap) m/z: $[M+H]^+$ Calcd for $C_{36}H_{65}N_4O_4$: 617.5000; Found 617.5011. TLC: Rf = 0.5 dichloromethane/methanol (9/1, v/v).

Scheme S4: Synthesis of *N*-thiomyristoyl lysine amide linker with free amine.

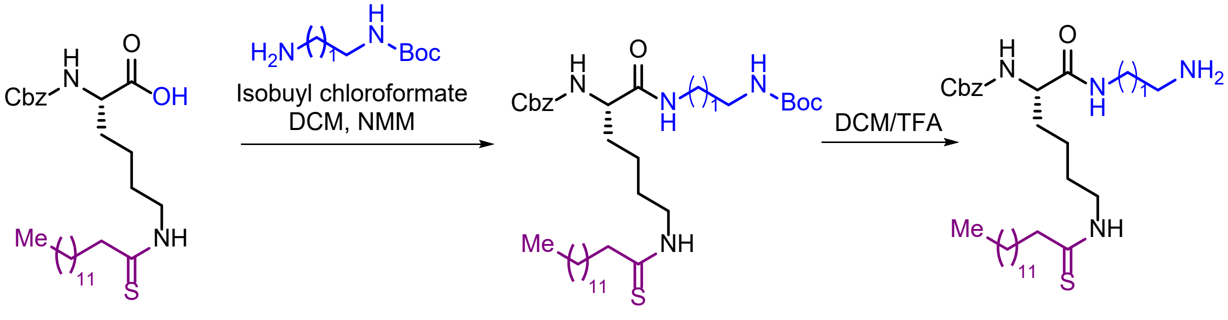

General procedure for Scheme S4: To a solution of *N*-Cbz thiomyristoyl lysine (NH4-3)[3] (1 equiv. 0.5 mmol, 253.4 mg) in dichloromethane (10 mL) at room temperature was added *N*-methyl morpholine (NMM, 2.0 equiv., 1.0 mmol, 110 µL), followed by the addition of isobutyl chloroformate (1.05 equiv., 0.52 mmol, 68.5 uL) drop wise. The solution was stirred at room temperature for 1 h. Then, corresponding amine (1.2 equiv., 0.6 mmol) was dissolved in dichloromethane and was poured to it. The resulting

reaction mixture was stirred at room temperature for 18 h. The solvent was evaporated, and the residue was purified by silica gel column chromatography using CH_2Cl_2/MeOH (9:1) as elute to afford corresponding amides derivatives. Then, the corresponding product was dissolved with 25% of TFA in DCM and the reaction mixture was stirred at room temperature for 2 hours. The reaction mixture was checked in LCMS to confirm the desired product and then solvent was removed by rotavapor. The residue was dissolved in methanol or toluene and evaporated by rotavapor. The process was repeated 6-7 times. The reside was dried over high vacuum overnight and proceeded for purification with DCM/methanol to afford desire product.

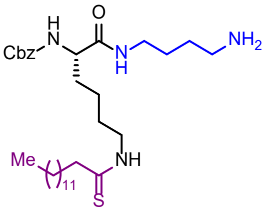

benzyl (S)-(1-((4-aminobutyl)amino)-1-oxo-6-tetradecanethioamidohexan-2-yl)carbamate (SJ-183). Eluent: Dichloromethane/methanol (9:1, v/v). Physical State: colorless semi-solid; Yield: 65%; ^{1}H NMR (500 MHz, $CDCl_3$) δ 8.28 (t, J = 5.4 Hz, 1H), 8.02 (s, 2H), 7.44 (s, 1H), 7.34 – 7.29 (m, 5H), 6.25 (d, J = 7.8 Hz, 1H), 5.09 – 5.00 (m, 2H), 4.14 (s, 1H), 3.58 (tt, J = 13.3, 7.2 Hz, 2H), 3.21 (s, 1H), 3.12 (s, 1H), 2.89 (s, 2H), 2.61 (d, J = 8.0 Hz, 2H), 1.74 (q, J = 7.1 Hz, 3H), 1.50 (s, 2H), 1.42 – 1.34 (m, 2H), 1.31 – 1.24 (m, 25H), 0.89 (t, J = 6.9 Hz, 3H). ^{13}C NMR (126 MHz, $CDCl_3$) δ 205.67, 173.04, 156.82, 136.02, 128.59, 128.30, 127.78, 67.10, 54.92, 46.89, 45.56, 39.41, 38.54, 32.19, 31.94, 29.73, 29.70, 29.68, 29.60, 29.48, 29.10, 27.07, 25.93, 24.43, 22.88, 22.70, 14.14. HRMS (DART/Orbitrap) m/z: $[M+H]^+$ Calcd for $C_{32}H_{57}N_4O_3S$: 577.4146; Found 577.4168. TLC: Rf = 0.45 dichloromethane/methanol (9/1, v/v).

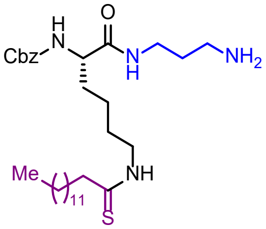

Benzyl (S)-(1-((3-aminopropyl)amino)-1-oxo-6-tetradecanethioamidohexan-2-yl)carbamate (SJ-200). Eluent: dichloromethane/methanol (9:1, v/v); Physical State: colorless solid; Yield: 62%; ^{1}H NMR (500 MHz, MeOD) δ 7.40 – 7.29 (m, 5H), 5.17 – 5.04 (m, 2H), 4.04 (dd, J = 9.1, 5.2 Hz, 1H), 3.59 (t, J = 7.2 Hz, 2H), 2.96 (t, J = 7.4 Hz, 2H), 2.60 (t, J = 7.6 Hz, 2H), 1.84 (h, J = 8.9 Hz, 3H), 1.76 – 1.62 (m, 5H), 1.50 – 1.39 (m, 2H), 1.31 (d, J = 10.0 Hz, 22H), 0.91 (t, J = 6.8 Hz, 3H). ^{13}C NMR (126 MHz, MeOD) δ 205.06, 174.65, 157.15, 136.70, 128.12, 127.69, 127.44, 66.37, 55.42, 45.72, 45.09, 36.70, 35.44, 31.70, 31.32, 29.46, 29.43, 29.39, 29.36, 29.27, 29.11, 28.60, 27.25, 26.92, 23.05, 22.37, 17.97, 13.13. HRMS (DART/Orbitrap) m/z: $[M+H]^+$ Calcd for $C_{31}H_{55}N_4O_3S$: 563.3989; Found 563.4016. TLC: Rf = 0.45 dichloromethane/methanol (9/1, v/v).

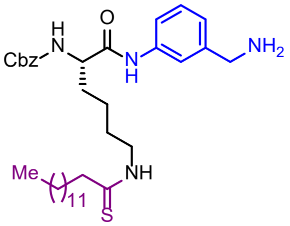

Benzyl (S)-(1-((3-(aminomethyl)phenyl)amino)-1-oxo-6-tetradecanethioamidohexan-2-yl)carbamate (**SJ-207RR**). Eluent: dichloromethane/methanol (9:1, v/v). Physical State: colorless semi-solid. Yield: 69%; ^{1}H NMR (500 MHz, CD_3OD) δ 7.73 (s, 1H), 7.47 (d, *J* = 8.2 Hz, 1H), 7.35 (qt, *J* = 13.6, 5.9 Hz, 6H), 7.18 (d, *J* = 7.5 Hz, 1H), 5.19 – 5.07 (m, 2H), 4.26 (dd, *J* = 9.0, 5.4 Hz, 1H), 3.98 (s, 2H), 3.61 (t, *J* = 7.2 Hz, 2H), 2.58 (t, *J* = 7.6 Hz, 2H), 2.17 (s, 1H), 1.89 (t, *J* = 12.4 Hz, 1H), 1.79 (tt, *J* = 9.5, 4.7 Hz, 1H), 1.71 (dt, *J* = 11.2, 5.8 Hz, 3H), 1.58 – 1.45 (m, 2H), 1.30 (d, *J* = 4.6 Hz, 20H), 0.91 (t, *J* = 6.9 Hz, 3H). ^{13}C NMR (126 MHz, CD_3OD) δ 205.11, 172.12, 157.17, 138.66, 136.75, 129.17, 128.58, 127.66, 127.45, 123.97, 120.24, 120.14, 66.35, 55.69, 45.69, 45.08, 29.41, 29.37, 29.33, 29.30, 29.23, 29.09, 29.06, 28.56, 26.99, 22.99, 22.35, 13.07. HRMS (DART/Orbitrap) m/z: $[M+H]^+$ Calcd for $C_{35}H_{55}N_4O_3S$: 612.4067; Found 612.4057. TLC: Rf = 0.45 dichloromethane/methanol (9/1, v/v).

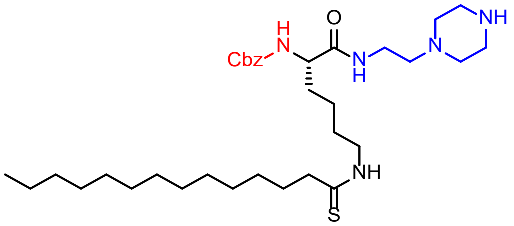

Benzyl (S)-(1-oxo-1-((2-(piperazin-1-yl)ethyl)amino)-6-tetradecanethioamidohexan-2-yl)carbamate (**NH4-10**): Eluent: dichloromethane/methanol (9/1, v/v). Physical State: colorless semi-solid. Yield: 54%; ^{1}H NMR (500 MHz, $CDCl_3$) δ 7.83 (s, 1H), 7.39 – 7.30 (m, 5H), 5.66 (d, J = 7.9 Hz, 1H), 5.11 (s, 2H), 4.18 (q, J = 7.6 Hz, 1H), 3.66 (tt, J = 12.8, 6.7 Hz, 2H), 3.57 – 3.30 (m, 6H), 2.70 – 2.46 (m, 7H), 1.92 – 1.82 (m, 1H), 1.82 – 1.64 (m, 5H), 1.47 (s, 9H), 1.44 (d, J = 7.4 Hz, 1H), 1.35 – 1.20 (m, 21H), 0.89 (t, J = 6.9 Hz, 3H). ^{13}C NMR (126 MHz, $CDCl_3$) δ 205.81, 171.80, 156.41, 154.49, 136.12, 128.59, 128.29, 127.98, 80.11, 67.07, 56.55, 54.54, 52.67, , 45.45, 32.35, 31.92, 29.69, 29.66, 29.65, 29.58, 29.55, 29.42, 29.36, 29.07, 28.40, 27.03, 22.69, 14.14. HRMS (DART/Orbitrap) m/z: $[M+H]^+$ Calcd for $C_{34}H_{69}N_5O_5S$: 718.4936; Found 718.4937. TLC: Rf = 0.45 dichloromethane/methanol (9/1, v/v).

Scheme S5: Synthesis of **SJ-106C analogs with shorter thioacyl chains**

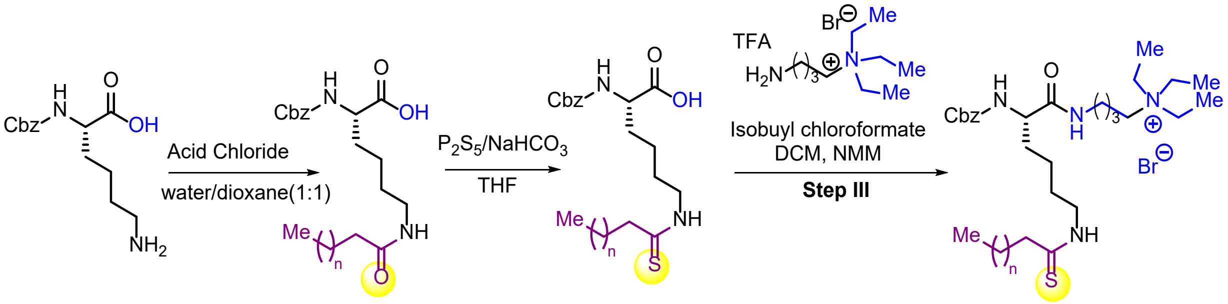

General procedure for Scheme S5: The several *N*-Cbz thioamide lysine derivatives with shorter acyl chains were synthesized from our previously reported method as synthesis of NH4-3.[3] To a solution of *N*-Cbz thioacyl lysine (1 equiv. 0.5 mmol, 253.4 mg) in dichloromethane (10 mL) at room temperature was added *N*-methyl morpholine (NMM, 2.0 equiv., 1.0 mmol, 110 µL), followed by the addition of isobutyl chloroformate (1.05 equiv., 0.52 mmol, 68.5 uL) drop wise. The solution was stirred at room temperature for 1 h. Then, compounds 3 (SJ-152) (1.2 equiv., 0.6 mmol) was dissolved in dichloromethane and was poured to it. The resulting reaction mixture was stirred at room temperature for 18 h. The solvent was evaporated, and the residue was purified by silica gel column chromatography using CH_2Cl_2/MeOH (9:1, v:v) as elute to afford desire product.

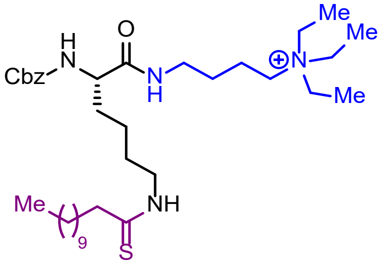

(S)-4-(2-(((benzyloxy)carbonyl)amino)-6-dodecanethioamidohexanamido)-N,N,N-triethylbutan-1-aminium bromide (SJ-202). Eluent: dichloromethane/methanol (9/1, v/v). Physical State: colorless semi-solid; Yield: 36%; 1H NMR (500 MHz, CD_3OD) δ 8.15 (s, 1H), 7.42 – 7.28 (m, 5H), 5.11 (s, 2H), 4.05 (t, J = 9.1 Hz, 1H), 3.61 (t, J = 7.2 Hz, 2H), 3.34 (s, 1H), 3.29 (q, J = 7.4 Hz, 7H), 3.20 (t, J = 8.4 Hz, 2H), 2.92 (s, 1H), 2.62 (t, J = 7.6 Hz, 2H), 1.81 (ddt, J = 15.4, 10.7, 5.3 Hz, 1H), 1.76 – 1.66 (m, 6H), 1.61 (q, J = 6.6 Hz, 2H), 1.54 – 1.47 (m, 1H), 1.45 – 1.38 (m, 1H), 1.36 – 1.24 (m, 25H), 0.91 (t, J = 6.8 Hz, 3H). ^{13}C NMR (126 MHz, CD_3OD) δ 205.00, 174.04, 157.00, 137.01, 128.19, 127.62, 127.16, 66.09, 63.74, 56.40, 55.61, 53.22, 52.54, 45.71, 45.09, 42.58, 37.88, 37.76, 31.70, 31.39, 29.49, 29.37, 29.28, 29.13, 29.11, 28.60, 26.95, 26.02, 23.10, 22.38, 18.43, 13.15, 6.44. HRMS (DART/Orbitrap) m/z: $[M]^+$ Calcd for $C_{35}H_{54}N_4O_3S$: 634.4850; Found 634.4835. TLC: Rf = 0.45 Dichloromethane/methanol (9/1, v/v).

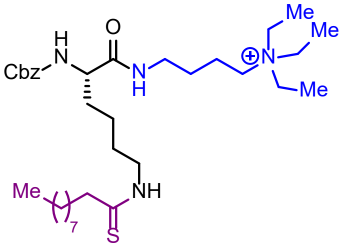

(S)-4-(2-(((benzyloxy)carbonyl)amino)-6-decanethioamidohexanamido)-N,N,N-triethylbutan-1-aminium bromide (SJ-204); Eluent: dichloromethane/methanol (9/1, v/v); Physical State: colorless semi-solid**;** Yield: 34%; 1H NMR (500 MHz, CD_3OD) δ 8.16 (s, 1H), 7.45 – 7.25 (m, 5H), 5.11 (s, 2H), 4.04 (dd, J = 9.0, 5.3 Hz, 1H), 3.85 (d, J = 12.5 Hz, 1H), 3.60 (t, J = 7.2 Hz, 2H), 3.45 (d, J = 12.6 Hz, 1H), 3.34 (d, J = 13.5 Hz, 1H), 3.29 (q, J = 7.3 Hz, 6H), 3.20 (t, J = 8.5 Hz, 2H), 2.92 (s, 1H), 2.61 (t, J = 7.6 Hz, 2H), 1.81 (ddd, J = 15.3, 10.2, 5.8 Hz, 1H), 1.75 – 1.66 (m, 6H), 1.60 (p, J = 6.7 Hz, 2H), 1.54 – 1.46 (m, 1H), 1.46 – 1.38 (m, 1H), 1.30 (td, J = 10.4, 5.5 Hz, 20H), 0.91 (t, J = 6.8 Hz, 3H). 13C NMR (126 MHz, CD_3OD) δ 204.99, 174.04, 156.99, 137.01, 128.20, 127.63, 127.17, 66.08, 63.73, 56.41, 55.61, 55.58, 53.21, 52.57, 52.55, 52.53, , 45.71, 45.09, 42.60, 37.89, 37.77, 31.66, 31.40, 29.49, 29.24, 29.13,

29.03, 28.59, 26.94, 26.00, 23.11, 22.37, 18.45, 13.15, 6.46. HRMS (DART/Orbitrap) m/z: $[M]^+$ Calcd for $C_{34}H_{61}N_4O_3S$: 606.4537; Found 606.4521**.** TLC: Rf = 0.45 dichloromethane/methanol (9/1, v/v).

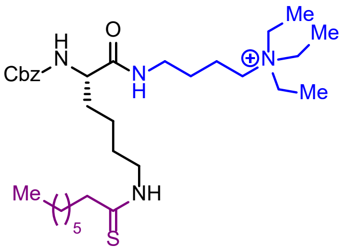

(S)-4-(2-(((benzyloxy)carbonyl)amino)-6-octanethioamidohexanamido)-N,N,N-triethylbutan-1-aminium bromide (SJ-213). Eluent: Dichloromethane/methanol (9/1, v/v); Physical State: colorless semi-solid; Yield: 38%; ^{1}H NMR (500 MHz, CD_3OD) δ 8.15 (s, 1H), 7.41 – 7.28 (m, 5H), 5.11 (s, 2H), 4.02 (dd, J = 8.9, 5.3 Hz, 1H), 3.61 (t, J = 7.2 Hz, 2H), 3.36 (d, J = 5.1 Hz, 2H), 3.28 (q, J = 7.3 Hz, 6H), 3.20 (t, J = 8.4 Hz, 2H), 2.61 (t, J = 7.6 Hz, 2H), 1.81 (dq, J = 13.6, 5.3 Hz, 1H), 1.75 – 1.66 (m, 6H), 1.61 (q, J = 6.6 Hz, 2H), 1.53 – 1.46 (m, 1H), 1.46 – 1.38 (m, 1H), 1.37 – 1.25 (m, 18H), 0.91 (t, J = 6.8 Hz, 3H). ^{13}C NMR (126 MHz, CD_3OD) δ 205.05, 173.98, 157.03, 137.00, 128.17, 127.61, 127.15, 66.08, 63.80, 56.37, 55.57, 53.27, 52.53, 52.51, , 45.70, 45.06, 42.64, 37.72, 31.51, 31.34, 29.47, 28.78, 28.54, 26.93, 26.01, 23.07, 22.29, 18.39, 13.06, 6.38. HRMS (DART/Orbitrap) m/z: $[M]^+$ Calcd for $C_{32}H_{57}N_4O_3S$: 577.4149; Found 577.4146. TLC: Rf = 0.45 dichloromethane/methanol (9/1, v/v).

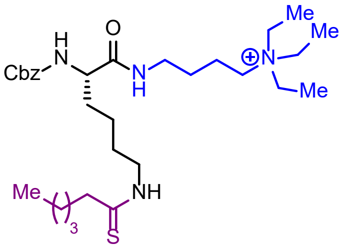

(S)-4-(2-(((benzyloxy)carbonyl)amino)-6-hexanethioamidohexanamido)-N,N,N-triethylbutan-1-aminium bromide (SJ-206). Eluent: dichloromethane/methanol (9/1, v/v); Physical State: colorless semi-solid; Yield: 35%; ^{1}H NMR (500 MHz, MeOD) δ 8.14 (s, 1H), 7.42 – 7.28 (m, 5H), 5.11 (s, 2H), 4.02 (dd, J = 8.9, 5.4 Hz, 1H), 3.61 (t, J = 7.2 Hz, 2H), 3.37 – 3.32 (m, 3H), 3.28 (q, J = 7.3 Hz, 6H), 3.20 (t, J = 8.4 Hz, 2H), 2.61 (t, J = 7.6 Hz, 2H), 1.77 – 1.67 (m, 6H), 1.60 (q, J = 6.7 Hz, 2H), 1.53 – 1.46 (m, 1H), 1.46 – 1.39 (m, 1H), 1.39 – 1.25 (m, 14H), 0.92 (t, J = 7.0 Hz, 3H). ^{13}C NMR (126 MHz, MeOD) δ 205.08, 174.00, 157.04, 136.99, 128.16, 127.61, 127.15, 66.08, 56.36, 55.56, 52.49, 45.65, 45.05, 37.81, 37.69, 31.31, 30.79, 29.15, 26.02, 23.06, 22.08, 18.36, 12.94, 6.34. HRMS (DART/Orbitrap) m/z: $[M]^+$ Calcd for $C_{30}H_{53}N_4O_3S$: 649.3838; Found 649.3833. TLC: Rf = 0.45 dichloromethane/methanol (9/1, v/v).

Scheme S6: Synthesis of SJ-112A.

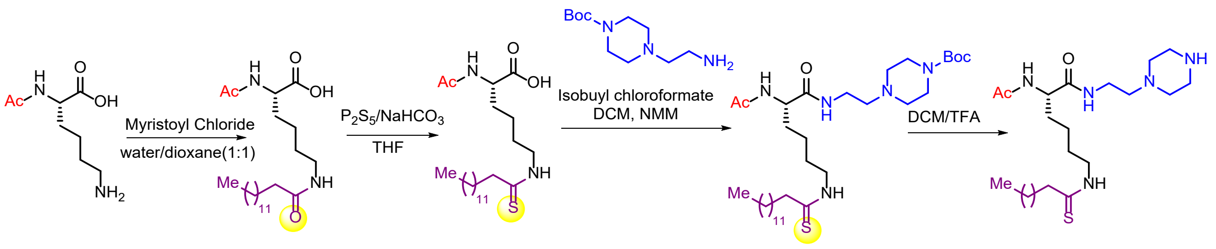

Procedure for Scheme S6: *N*-Acetyl thiomyristoyl lysine (**11c**) was synthesized from our previously reported method.[3, 5] To a solution of *N*-Acetyl thiomyristoyl lysine (**11c**) (1 equiv. 0.5 mmol, 207.2 mg) in dichloromethane (10 mL) at room temperature was added *N*-methyl morpholine (NMM, 2.0 equiv., 1.0 mmol, 110 µL), followed by the addition of isobutyl chloroformate (1.05 equiv., 0.52 mmol, 68.5 uL) drop wise. The solution was stirred at room temperature for 1 h. Then, 2-(piperazin-1-yl)ethan-1-amine (1.2 equiv., 0.6 mmol) was dissolved in dichloromethane and was poured to it. The resulting reaction mixture was stirred at room temperature for 18 h. The solvent was evaporated, and the residue was purified by silica gel column chromatography using CH_2Cl_2/MeOH (9/1) as elute to afford corresponding amide (**11d**). Then, the compound **11d** was dissolved with 25% of TFA in DCM and the reaction mixture was stirred at room temperature for 2 hours. The reaction mixture was checked in LCMS to confirm the desired product and then solvent was removed by rotavapor. The residue was dissolved in methanol or toluene and evaporated by rotavapor. The process was repeated 6-7 times. The reside was dried over high vacuum overnight and proceeded for purification with CH_2Cl_2/MeOH (9/1) to afford desire product **11e**.

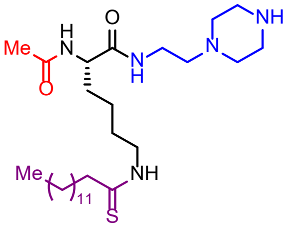

(S)-2-acetamido-N-(2-(piperazin-1-yl)ethyl)-6 tetradecanethioamidohexanamide (**SJ-112A**). Eluent: dichloromethane/methanol (9:1), (v/v); Physical State: colorless solid; Yield: 57%; . ^{1}H NMR (500 MHz, MeOD) δ 4.14 (dd, *J* = 8.4, 5.9 Hz, 1H), 3.60 (dt, *J* = 15.2, 6.7 Hz, 3H), 3.46 (dq, *J* = 10.2, 5.1 Hz, 5H), 3.26 (s, 4H), 3.04 (p, *J* = 6.3 Hz, 2H), 2.60 (t, *J* = 7.6 Hz, 2H), 2.03 (s, 3H), 1.88 – 1.77 (m, 1H), 1.78 – 1.63 (m, 5H), 1.53 – 1.38 (m, 2H), 1.38 – 1.25 (m, 20H), 0.92 (t, *J* = 6.9 Hz, 3H). ^{13}C NMR (126 MHz, $CDCl_3$) δ 206.00, 172.43, 170.57, 61.77, 56.25, 53.44, 51.59, 50.16, 47.05, 45.52, 43.91, 32.43, 31.93, 29.71, 29.67, 29.65, 29.55, 29.42, 29.37, 29.33, 29.07, 27.23, 26.91, 23.20, 22.70, 22.52, 14.14. HRMS (DART/Orbitrap) m/z: $[M]^+$ Calcd for $C_{28}H_{55}N_5O_2S$: 526.4149; Found 526.4170.

References:

1 (a)Jing, H.; Hu, J.; He, B.; Negrón Abril, Y. L.; Stupinski, J.; Weiser, K.; Carbonaro, M.; Chiang, Y.-L.; Southard, T.; Giannakakou, P.; Weiss, R. S.; Lin, H., A SIRT2-Selective Inhibitor Promotes c-Myc Oncoprotein Degradation and Exhibits Broad Anticancer Activity. *Cancer Cell* **2016,** *29* (3), 297-310. (B) Spiegelman, N. A.; Price, I. R.; Jing, H.; Wang, M.; Yang, M.; Cao, J.; Hong, J. Y.; Zhang, X.; Aramsangtienchai, P.; Sadhukhan, S.; Lin, H., Direct Comparison of SIRT2

Inhibitors: Potency, Specificity, Activity-Dependent Inhibition, and On-Target Anticancer Activities. ChemMedChem 2018, 13 (18), 1890-1894.

2 Li, M.; Chiang, Y.-L.; Lyssiotis, C. A.; Teater, M. R.; Hong, J. Y.; Shen, H.; Wang, L.; Hu, J.; Jing, H.; Chen, Z.; Jain, N.; Duy, C.; Mistry, S. J.; Cerchietti, L.; Cross, J. R.; Cantley, L. C.; Green, M. R.; Lin, H.; Melnick, A. M., Non-oncogene Addiction to SIRT3 Plays a Critical Role in Lymphomagenesis. Cancer Cell 2019, 35 (6), 916-931.e9.

3 Hong, J. Y.; Price, I. R.; Bai, J. J.; Lin, H., A Glycoconjugated SIRT2 Inhibitor with Aqueous Solubility Allows Structure-Based Design of SIRT2 Inhibitors. *ACS Chemical Biology* **2019,** *14* (8), 1802-1810.)

4 Hong, J. Y.; Fernandez, I.; Anmangandla, A.; Lu, X.; Bai, J. J.; Lin, H., Pharmacological Advantage of SIRT2-Selective versus pan-SIRT1–3 Inhibitors. ACS Chemical Biology 2021, 16 (7), 1266-1275.

5 Hong, J. Y.; Jing, H.; Price, I. R.; Cao, J.; Bai, J. J.; Lin, H., Simultaneous Inhibition of SIRT2 Deacetylase and Defatty-Acylase Activities via a PROTAC Strategy. ACS Medicinal Chemistry Letters 2020, 11 (11), 2305-2311.

NMR Spectra:

NH4-1 ^{1}H NMR

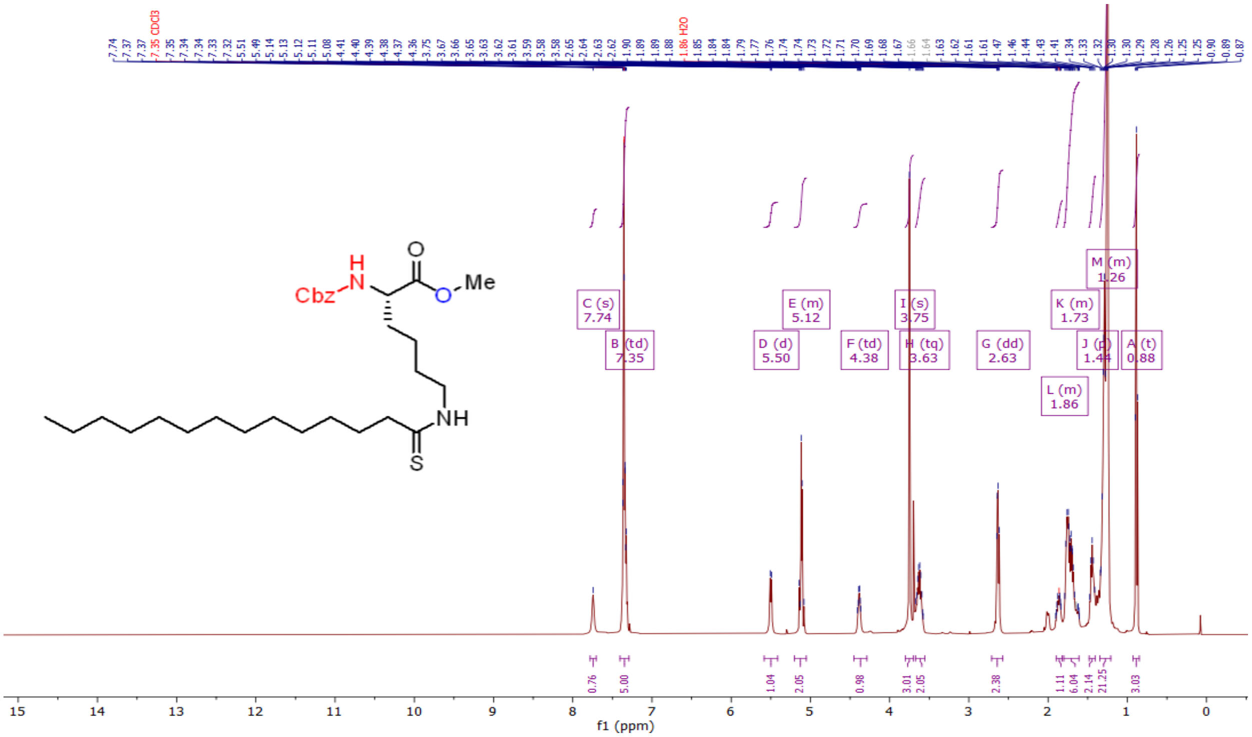

NH4-1 ^{13}C NMR

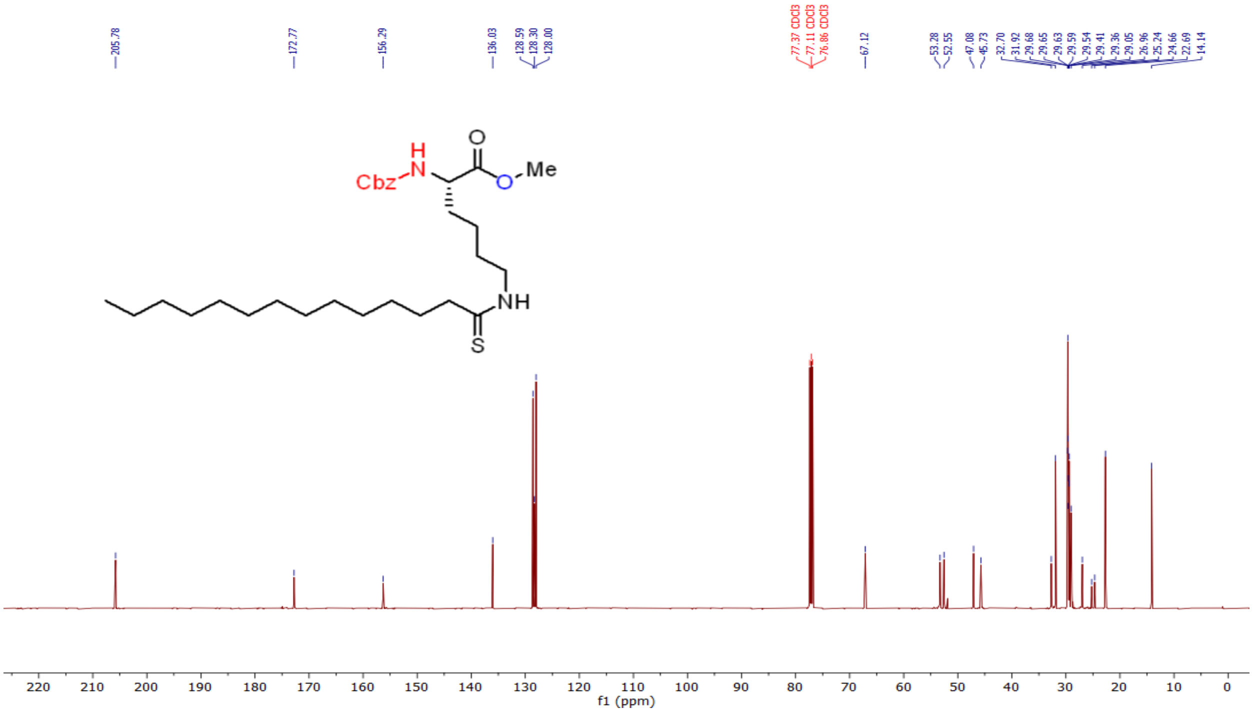

NH4-2 ^{1}H NMR

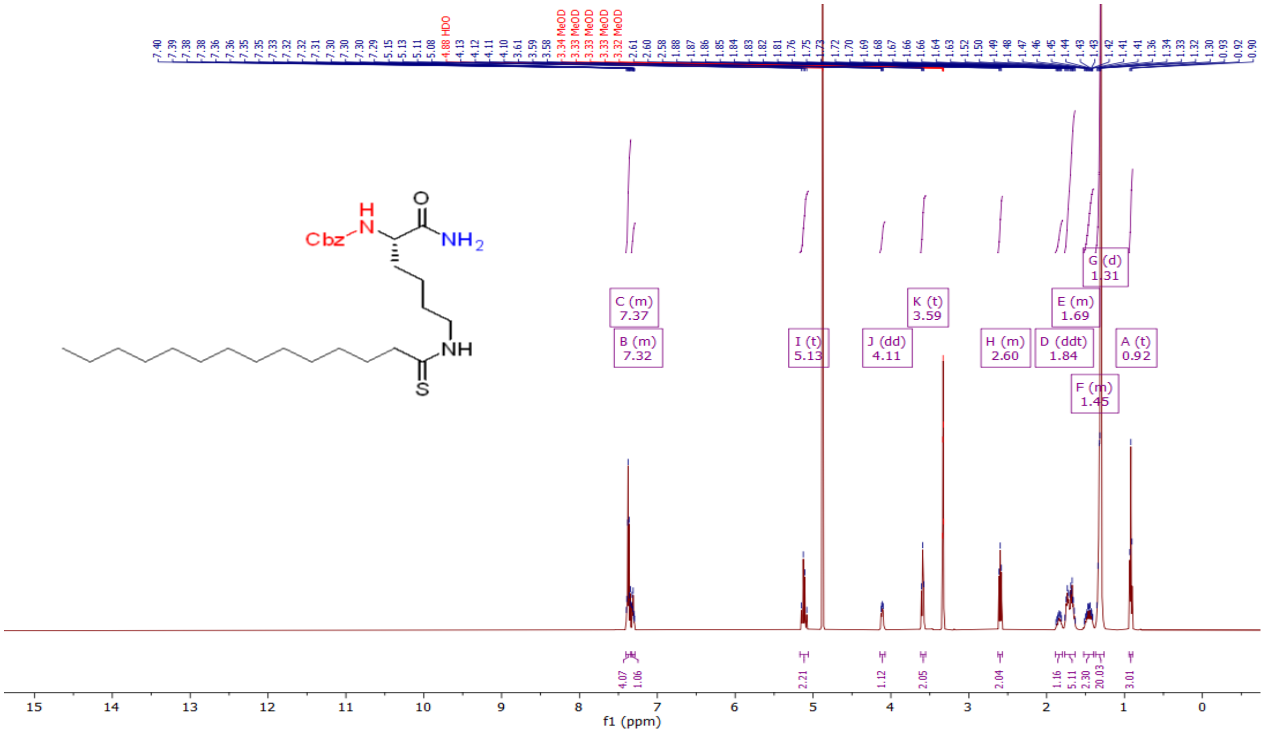

NH4-2 ^{13}C NMR

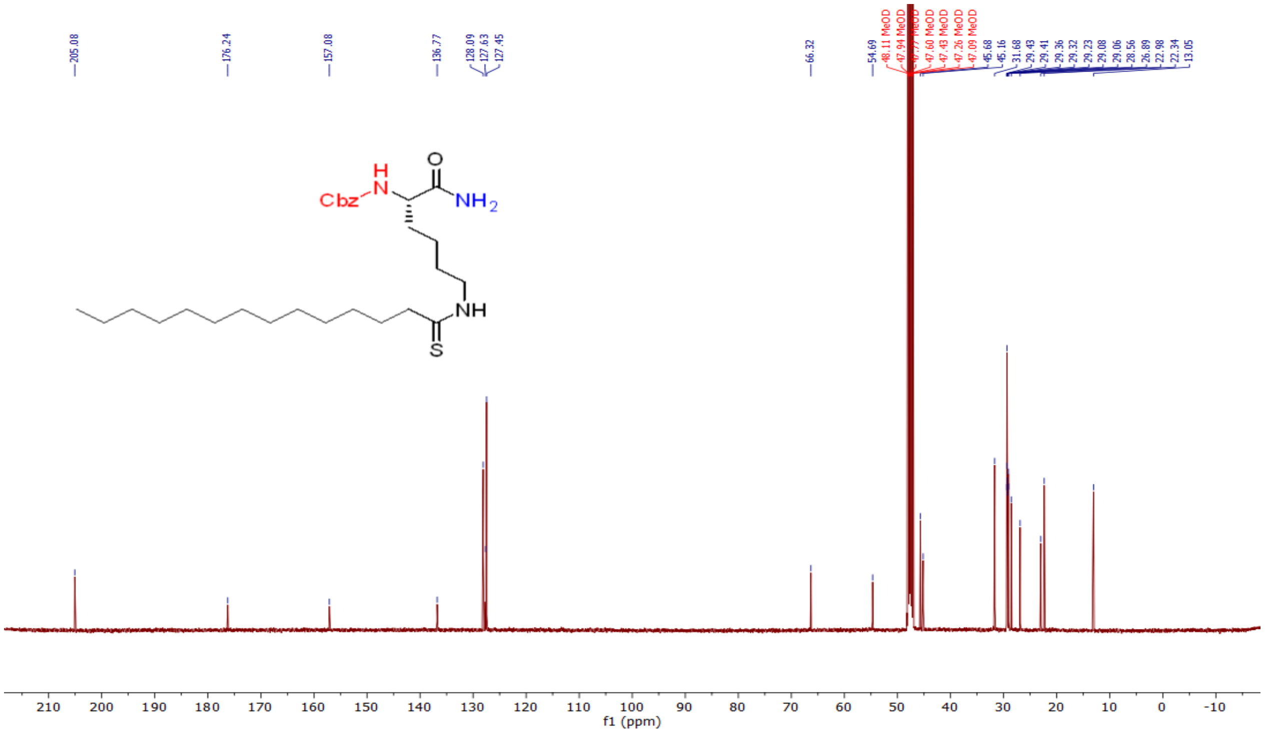

NH4-3 ^{1}H NMR

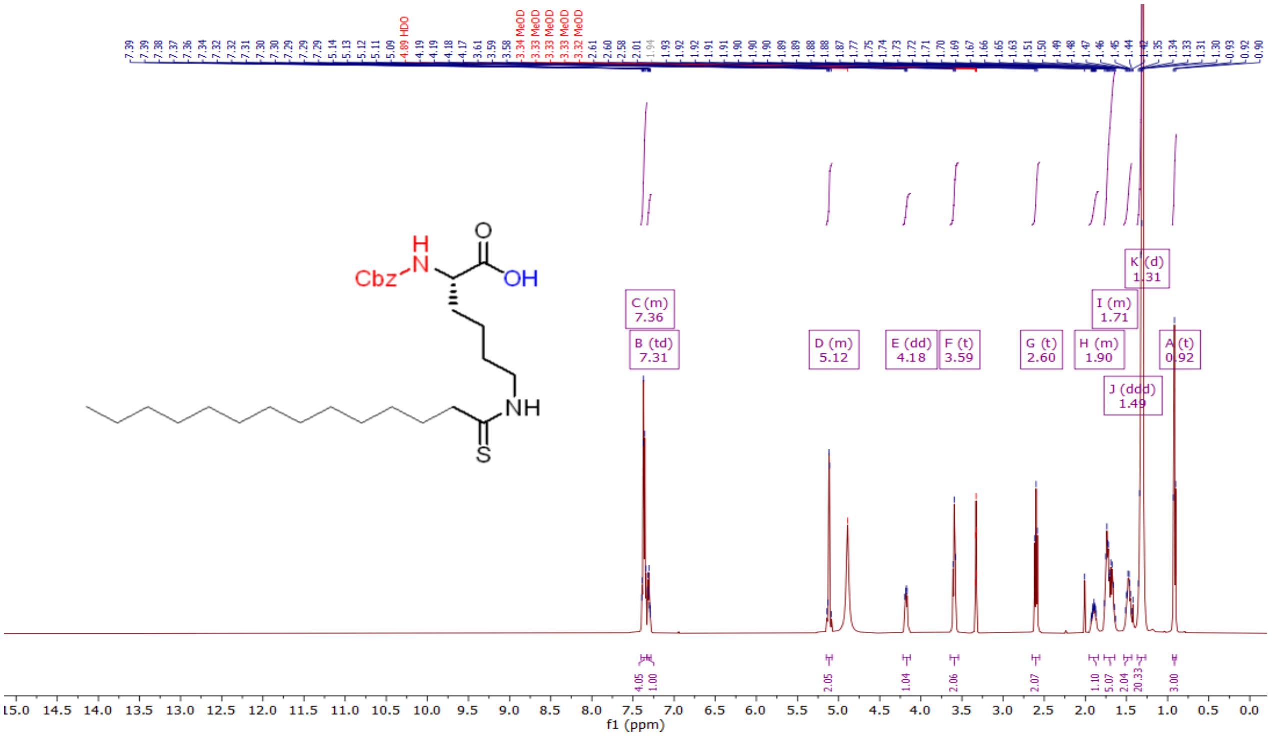

NH4-3 ^{13}C NMR

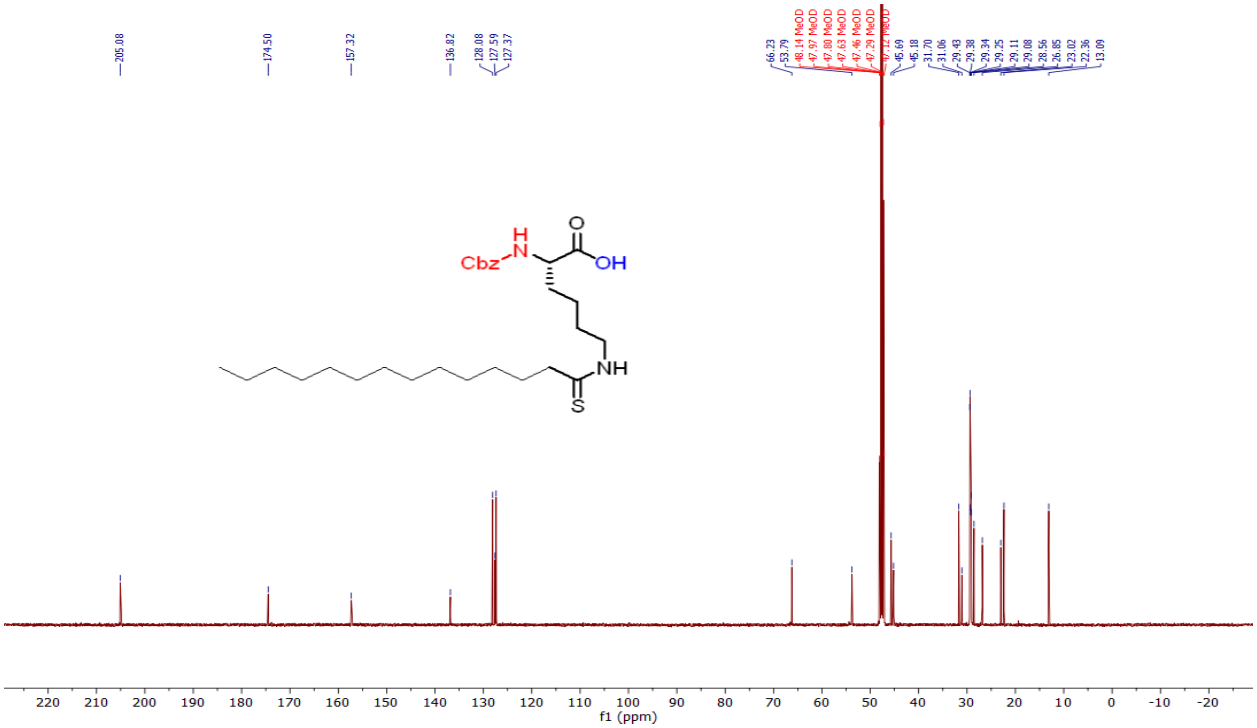

NH4-9 ^{1}H NMR

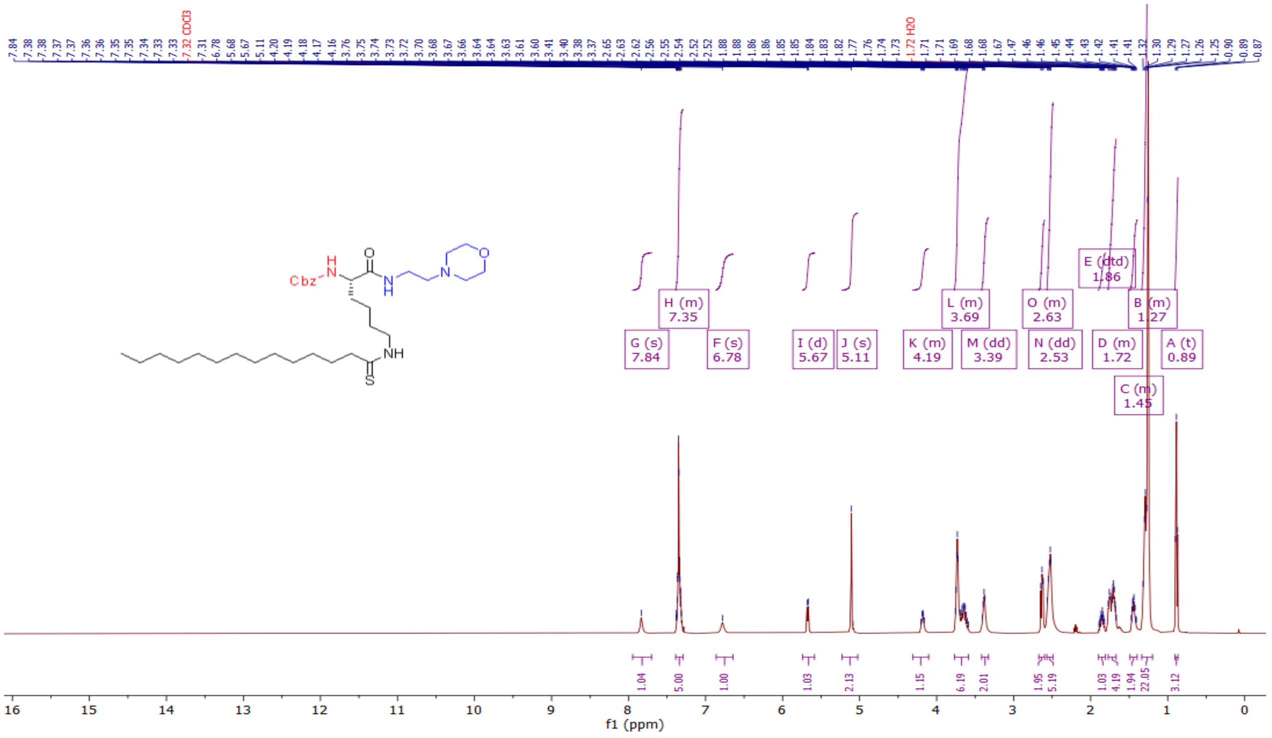

NH4-9 ^{13}C NMR

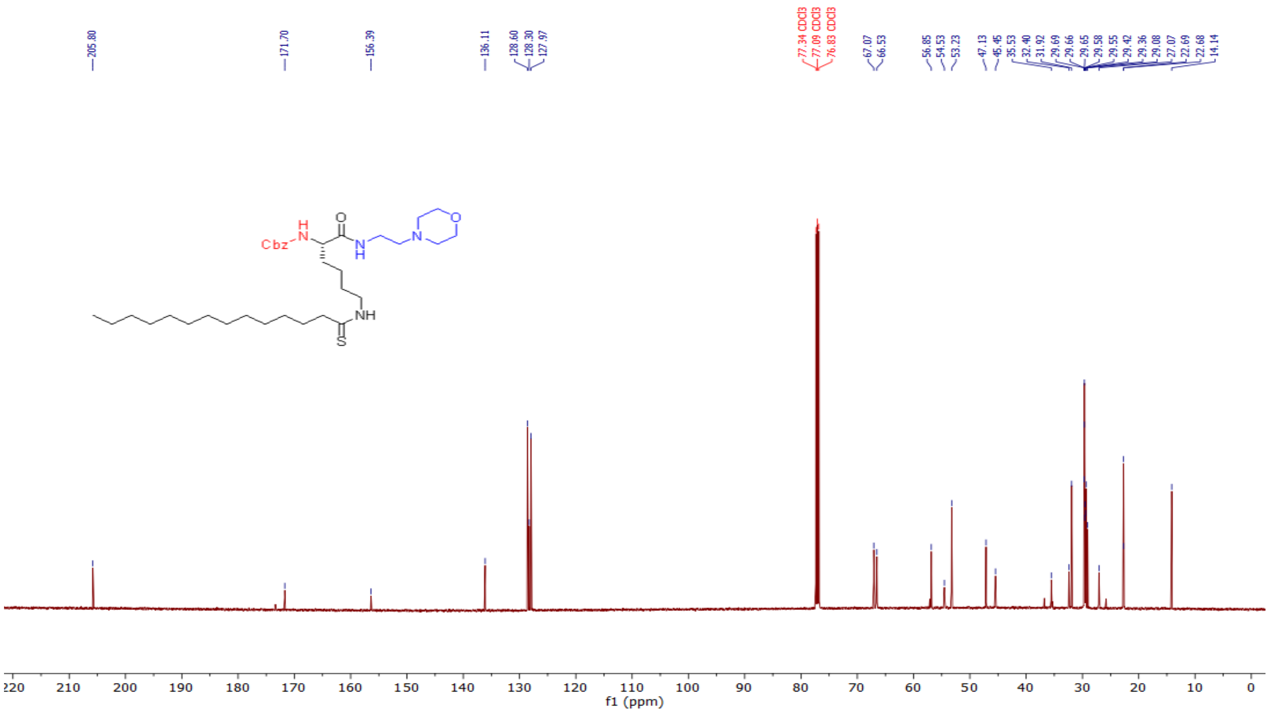

NH4-10 ^{1}H NMR

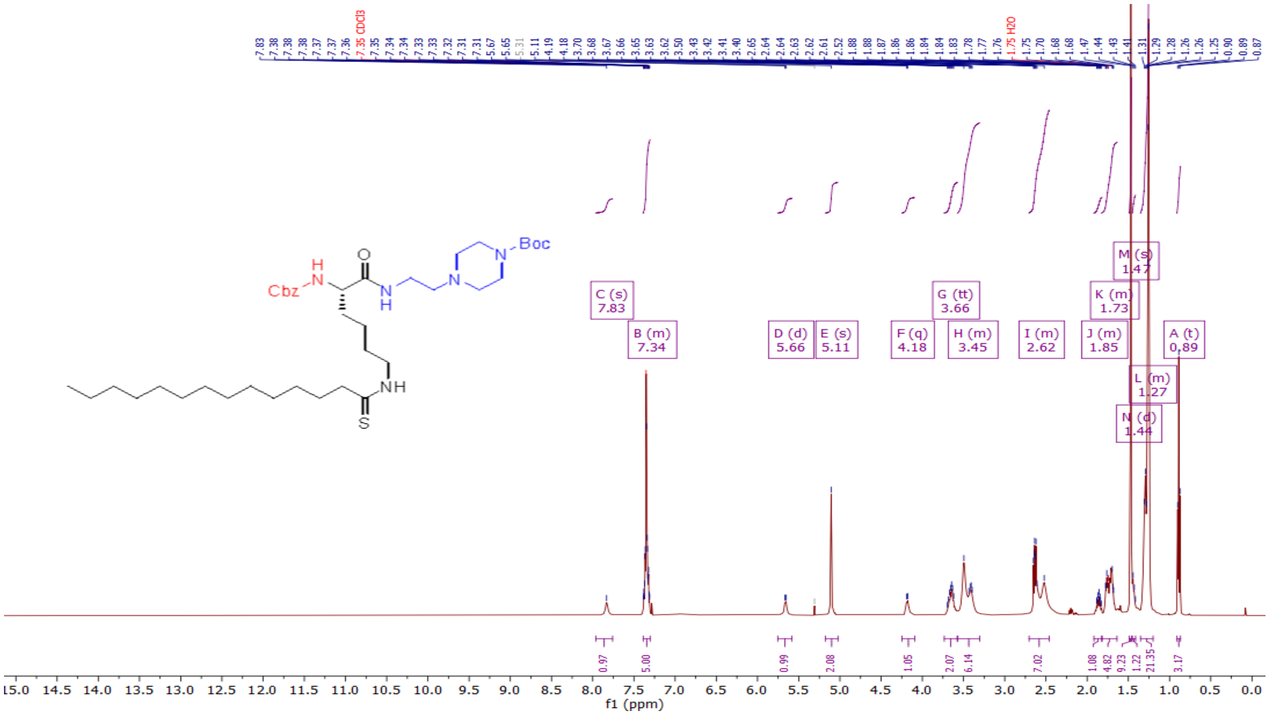

NH4-10 ^{13}C NMR

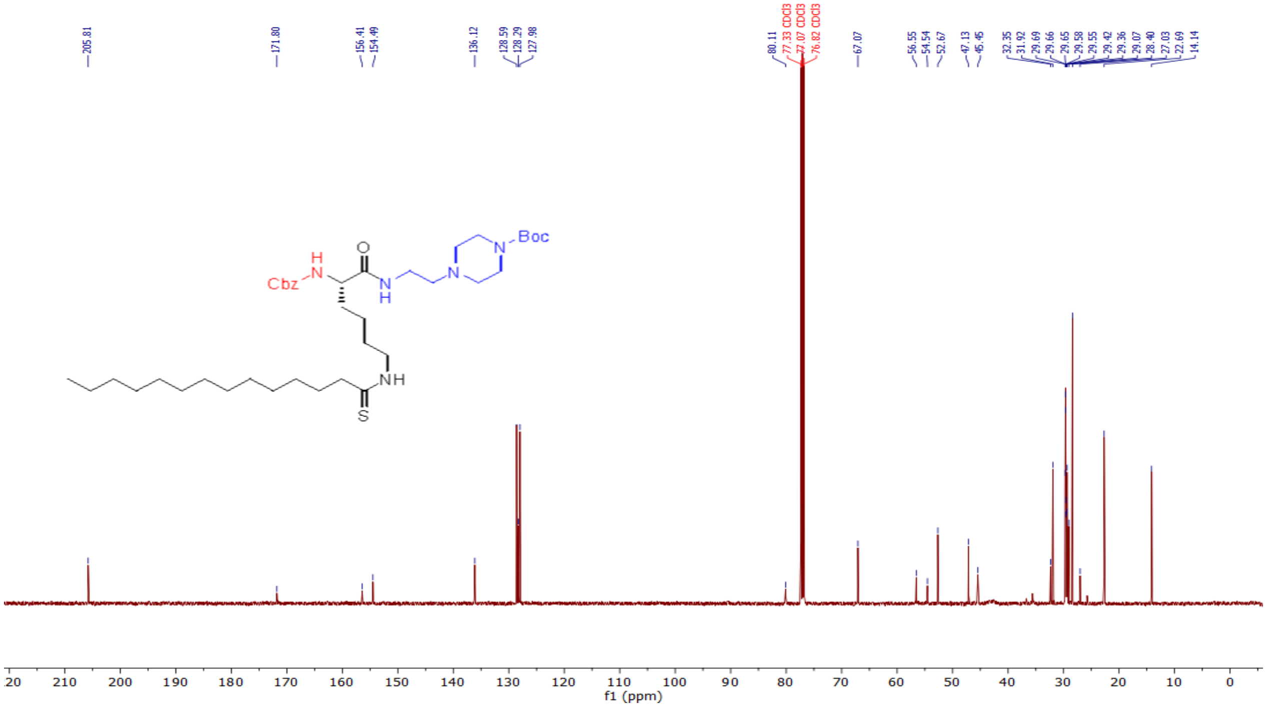

NH4-P-104 ^{1}H NMR

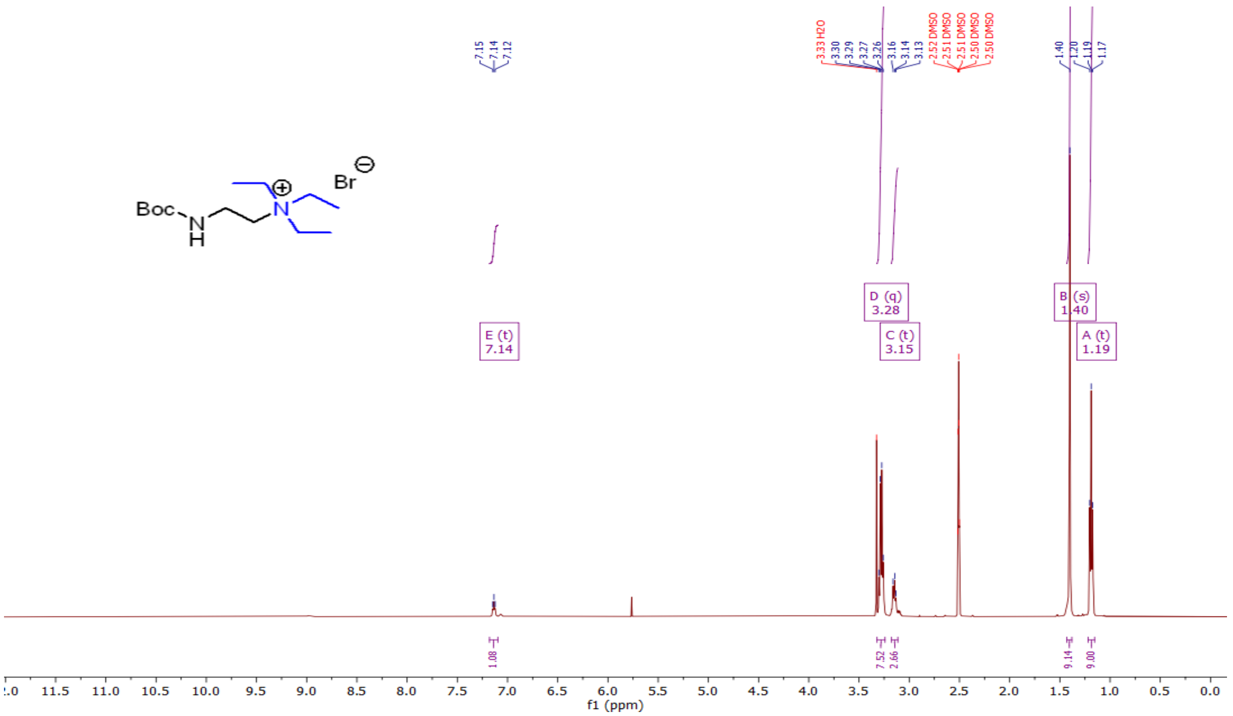

NH4-P-104 ^{13}C NMR

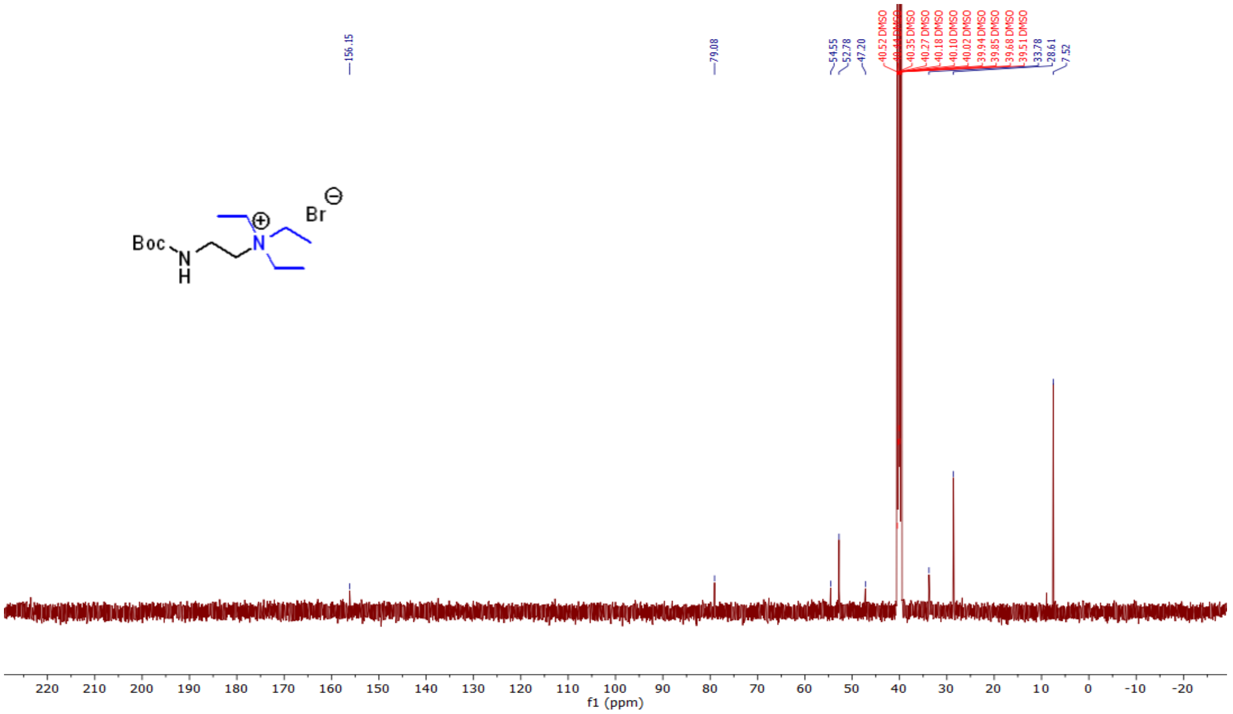

SJ-161 ^{1}H NMR

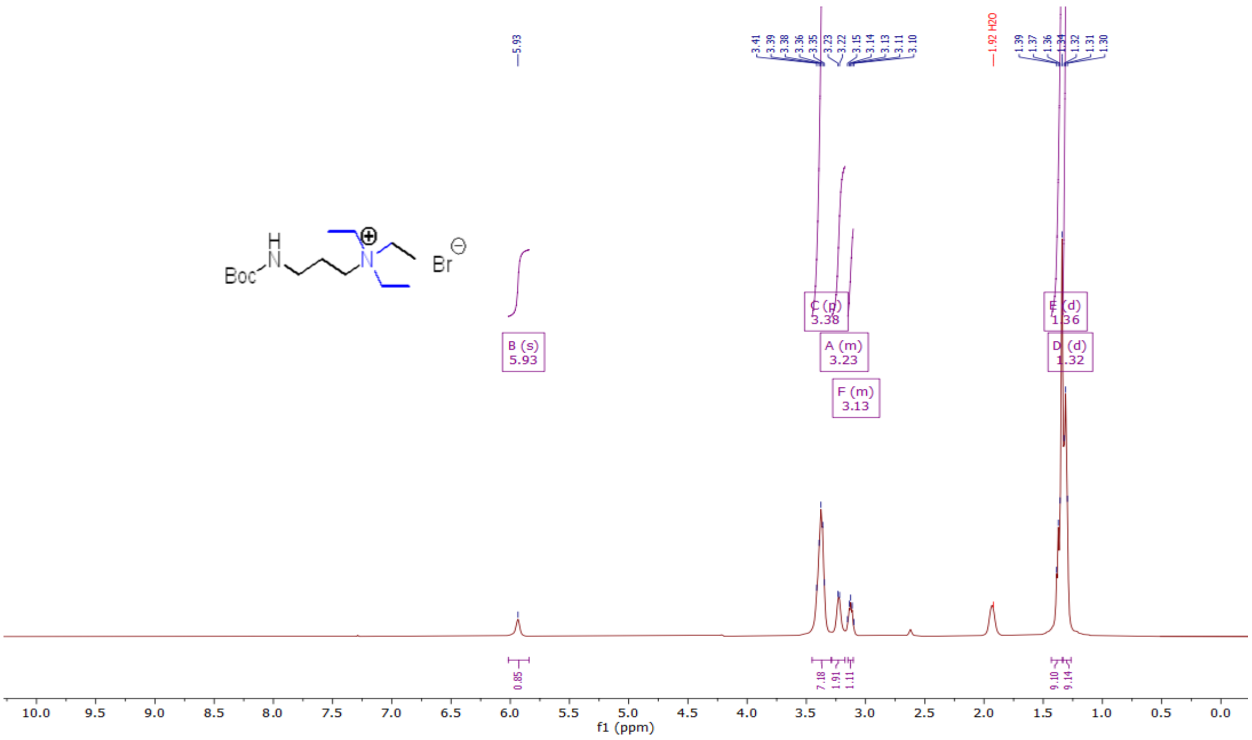

SJ-161 ^{13}C NMR

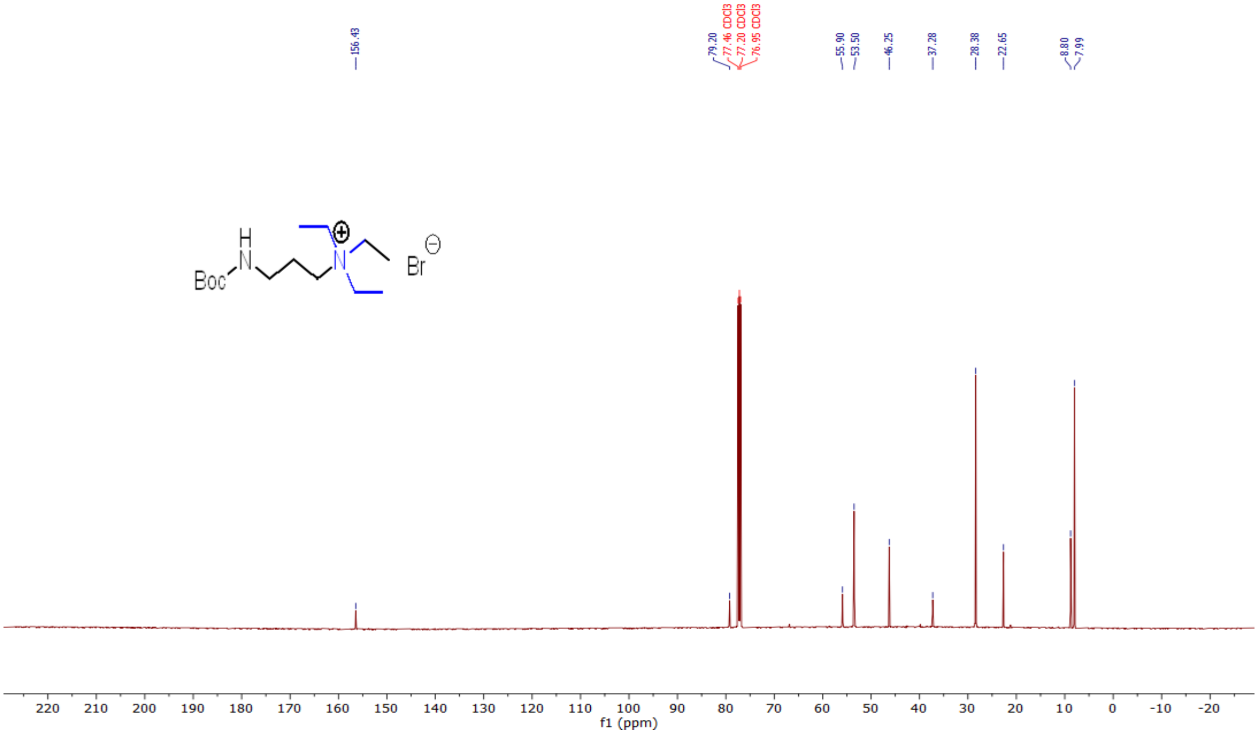

SJ-152 ^{1}H NMR

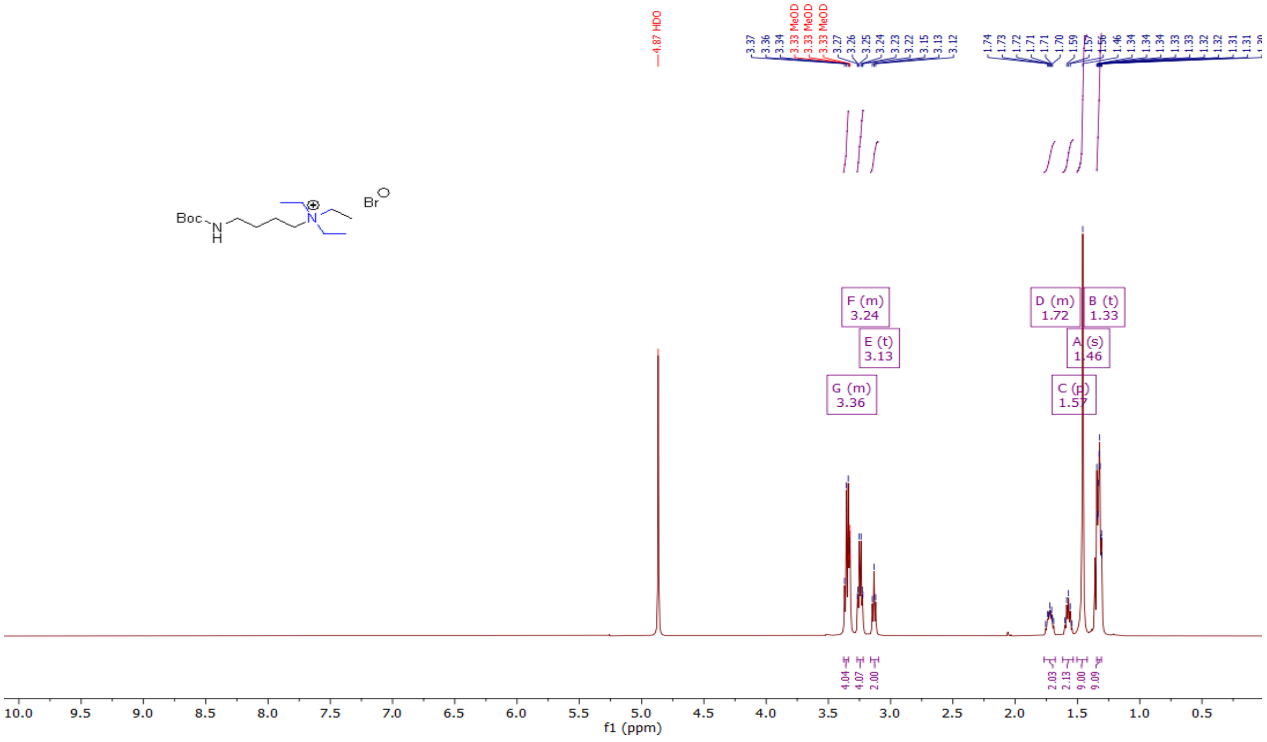

SJ-152 ^{13}C NMR

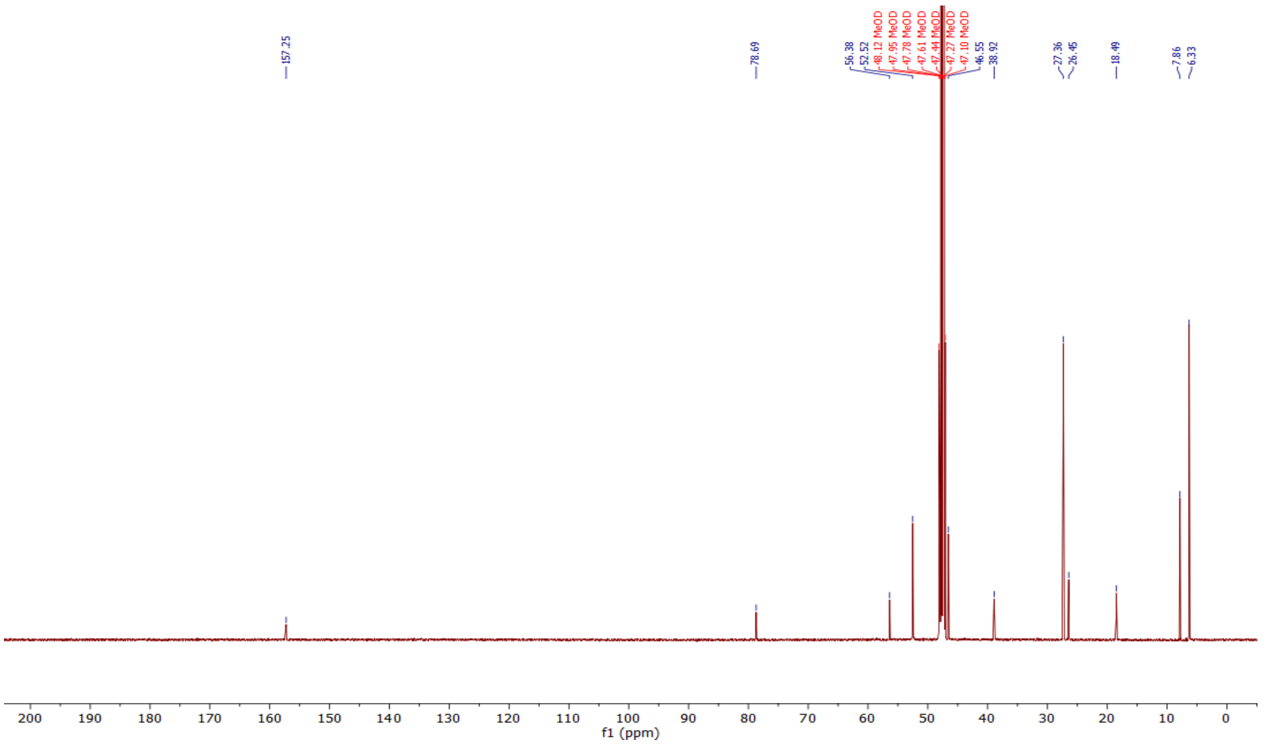

SJ-167 ^{1}H NMR

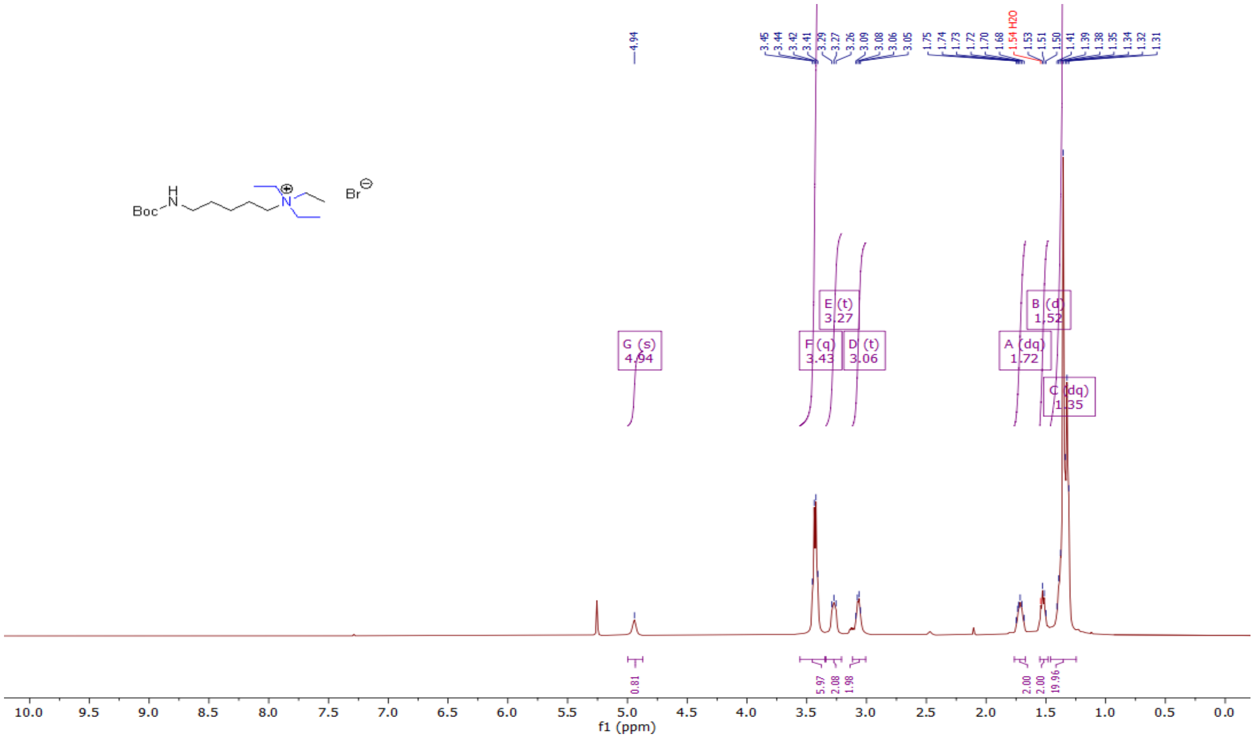

SJ-167 ^{13}C NMR

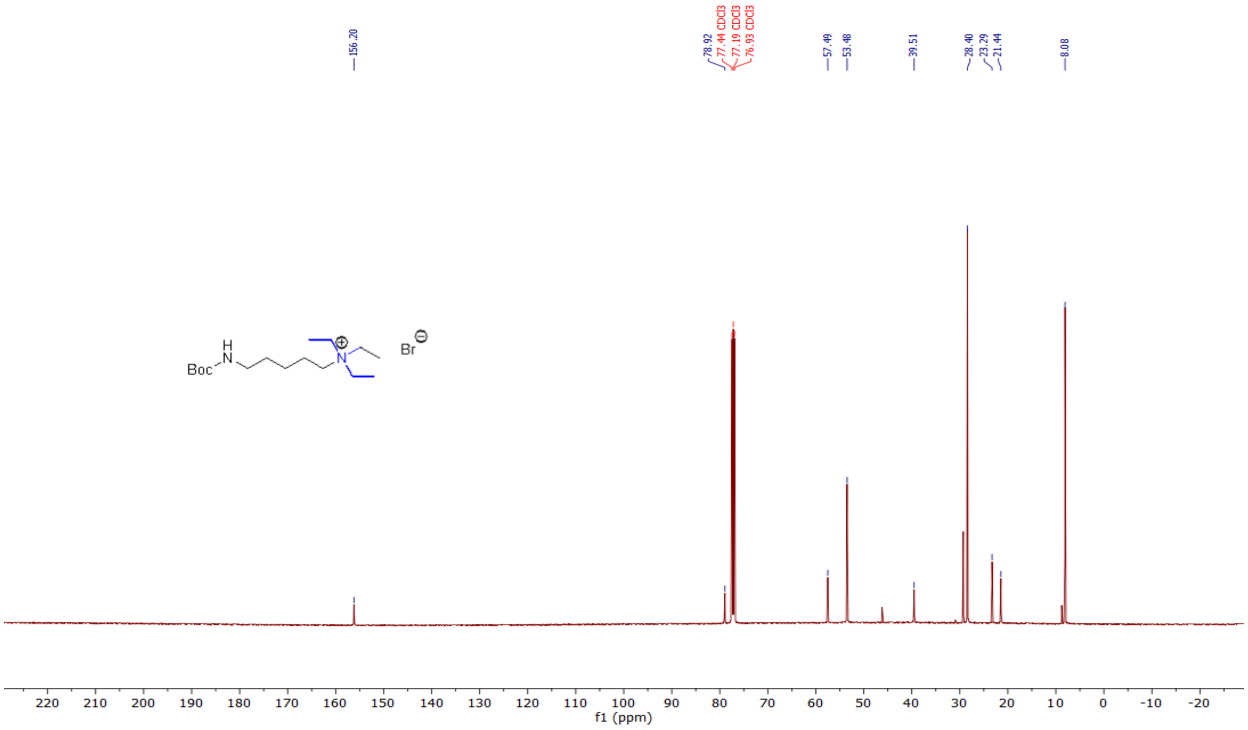

SJ-214 ^{1}H NMR

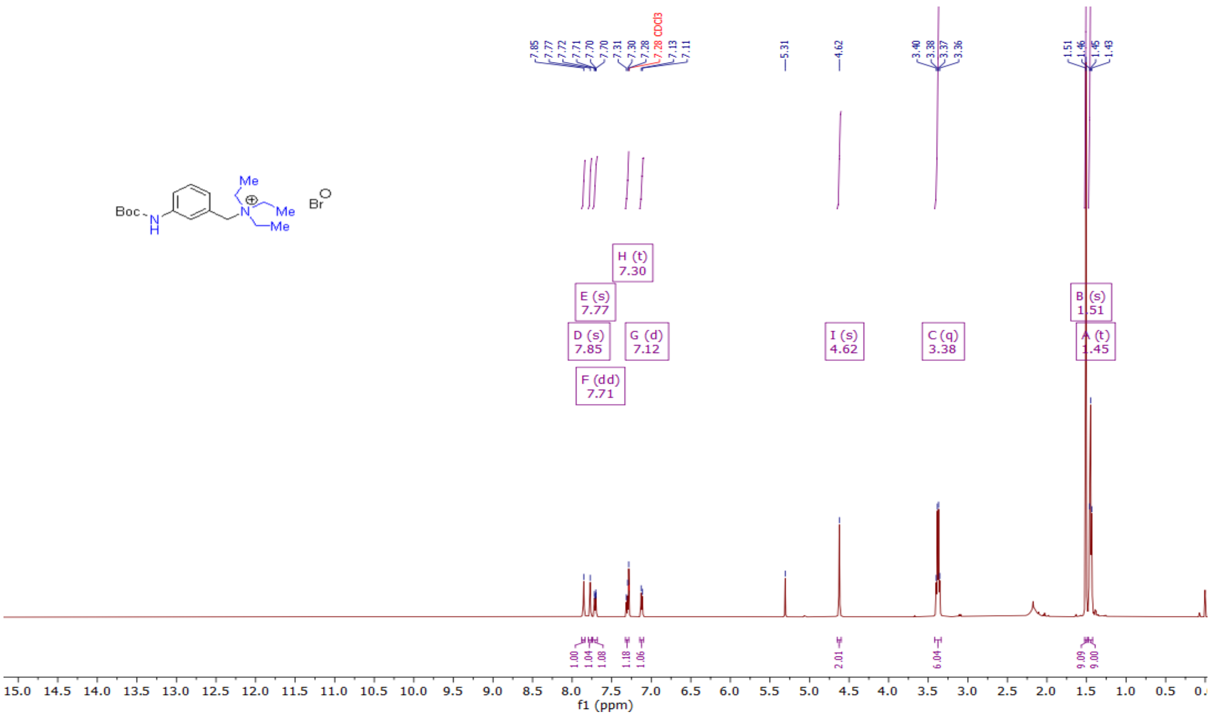

SJ-214 ^{13}C NMR

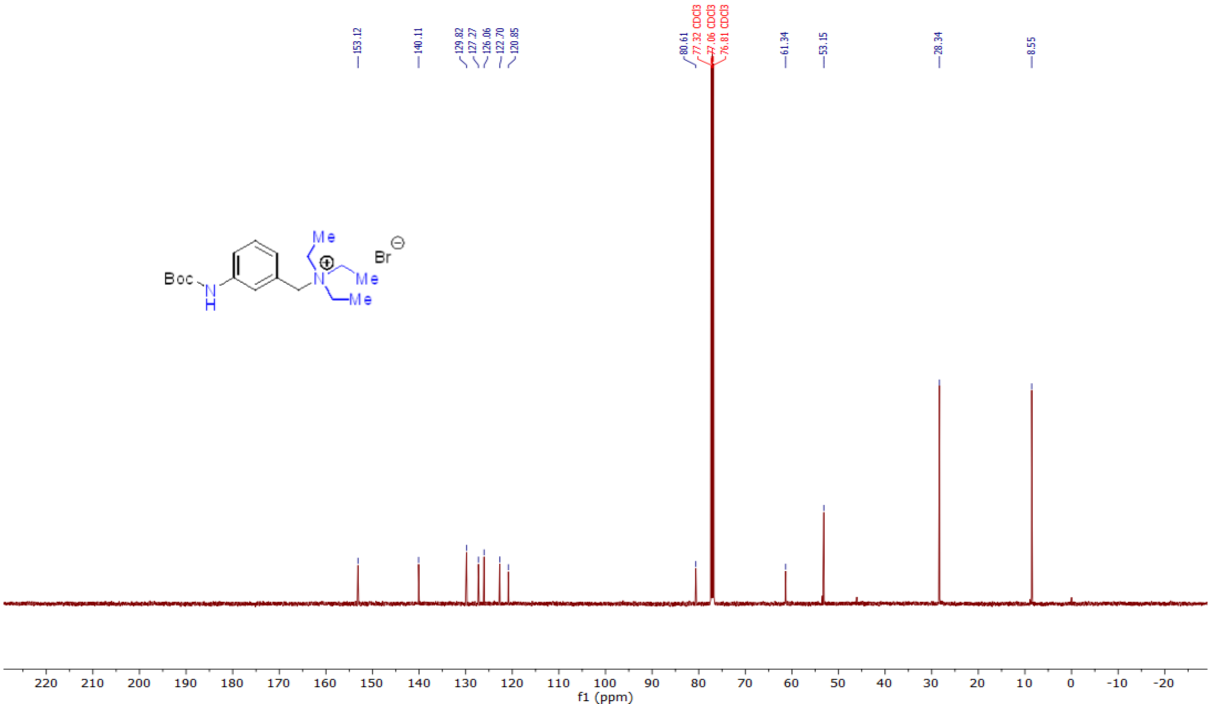

NH6-10 ^{1}H NMR

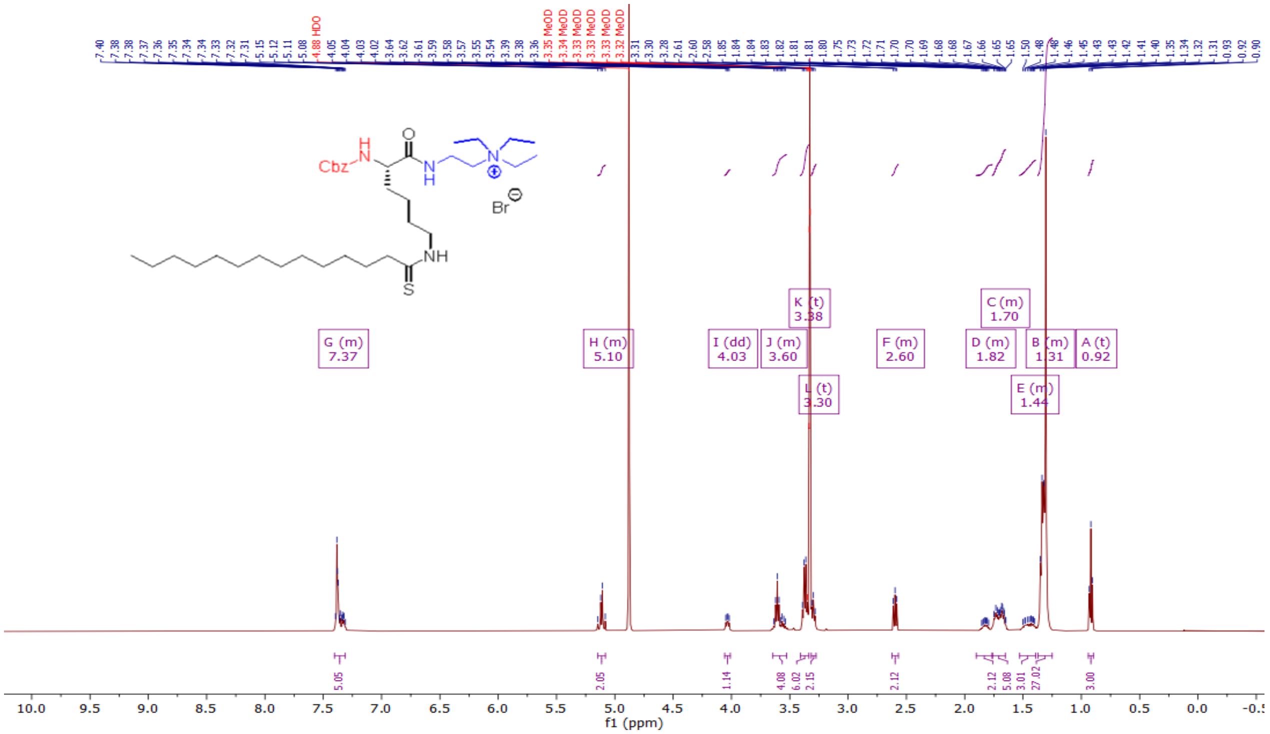

NH6-10 ^{13}C NMR

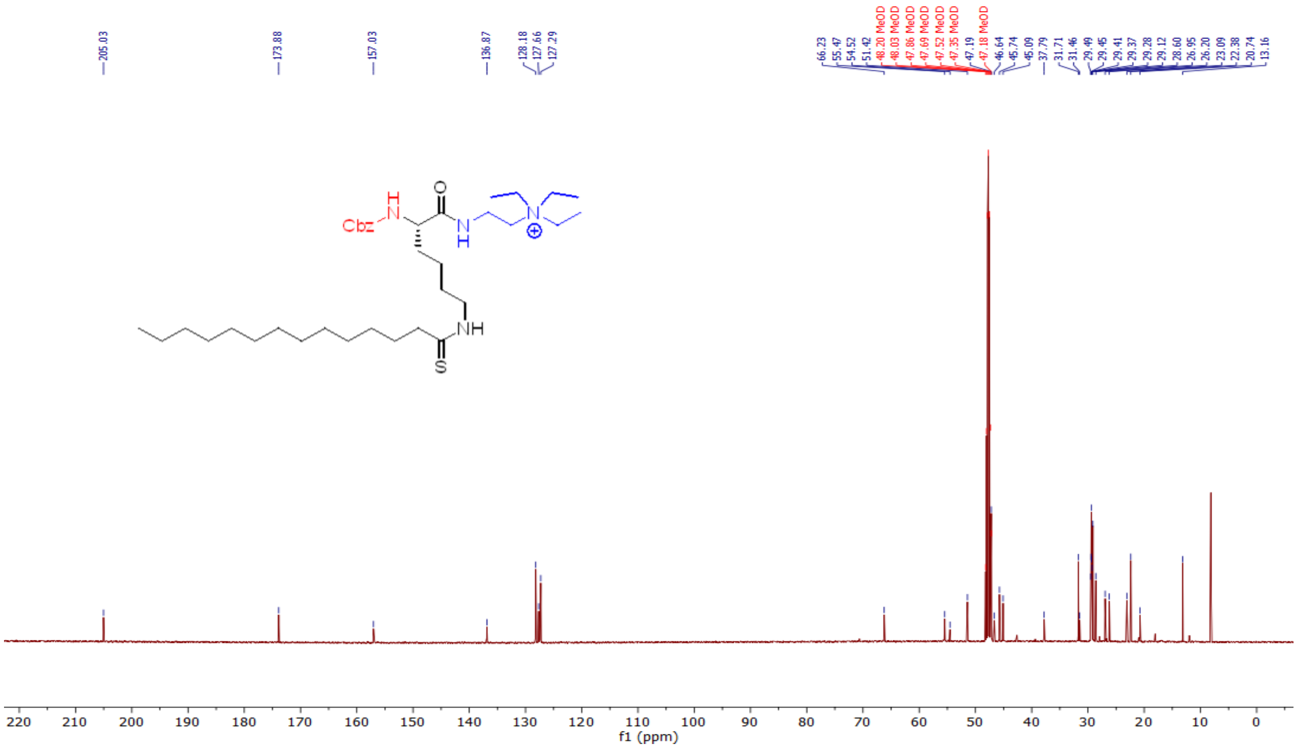

SJ-163 ^{1}H NMR

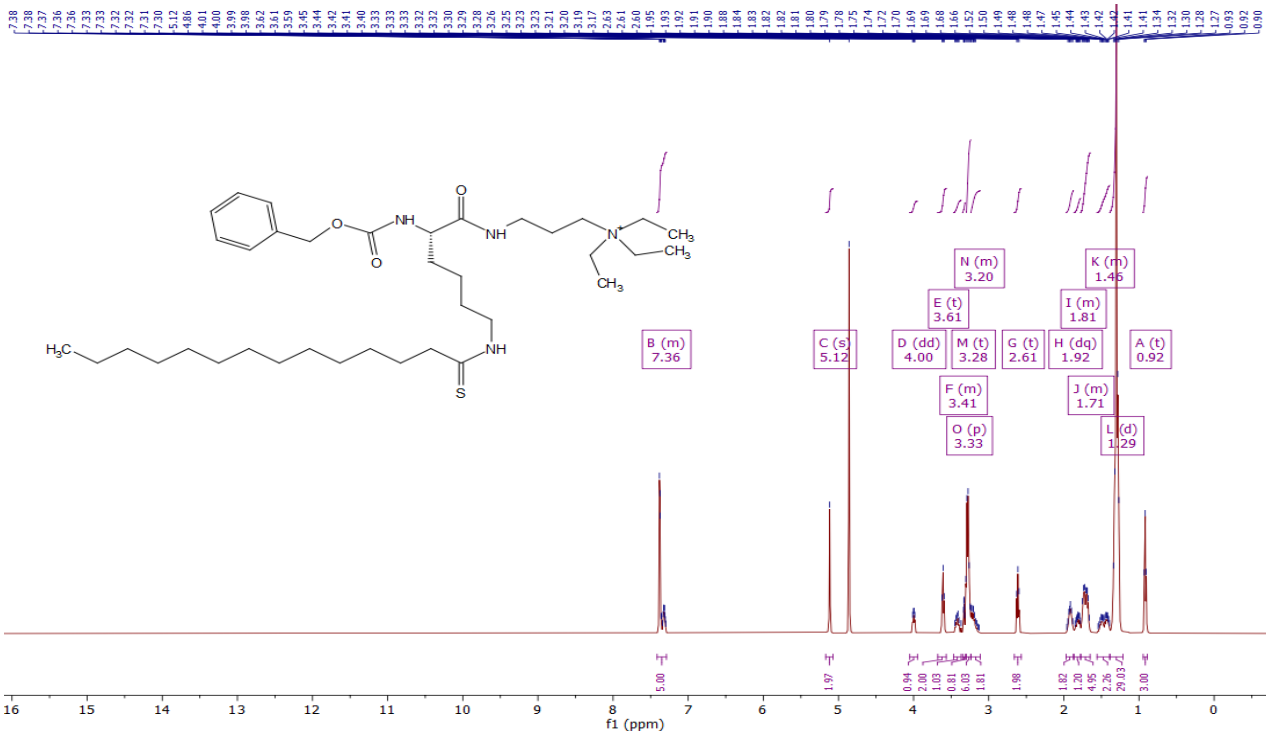

SJ-163 ^{13}C NMR

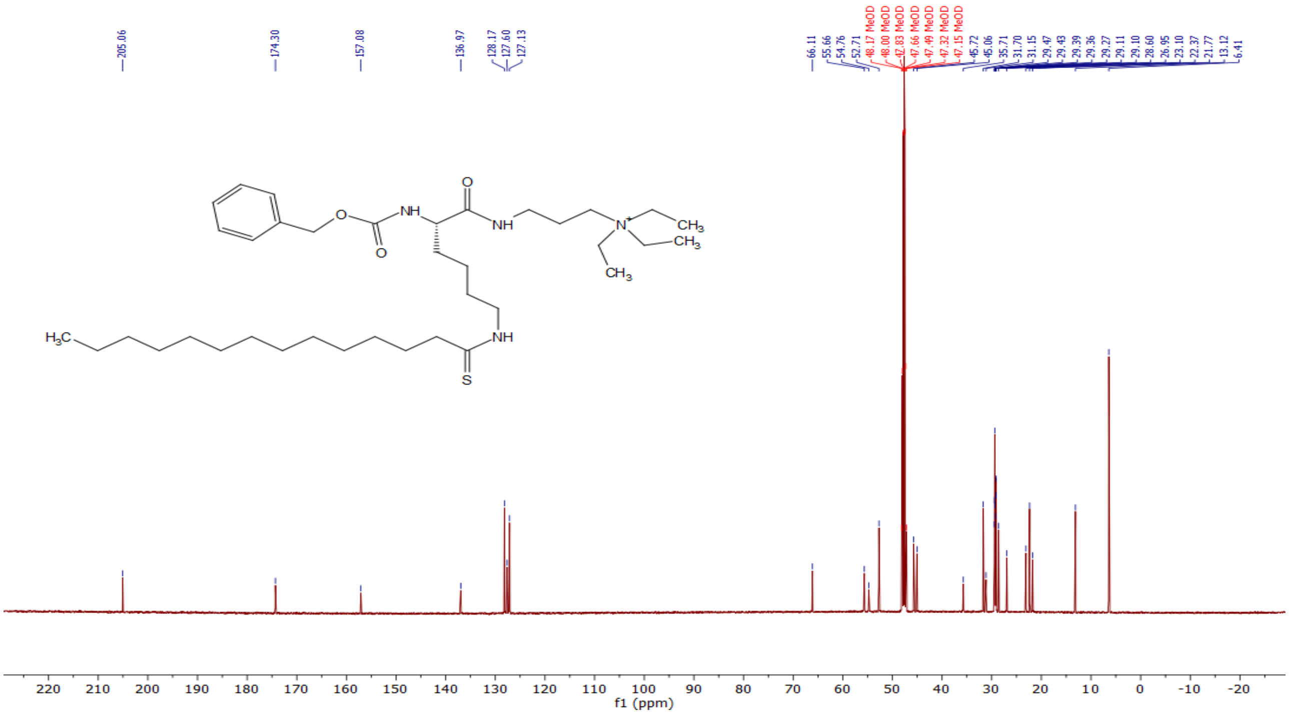

SJ-106C ^{1}H NMR

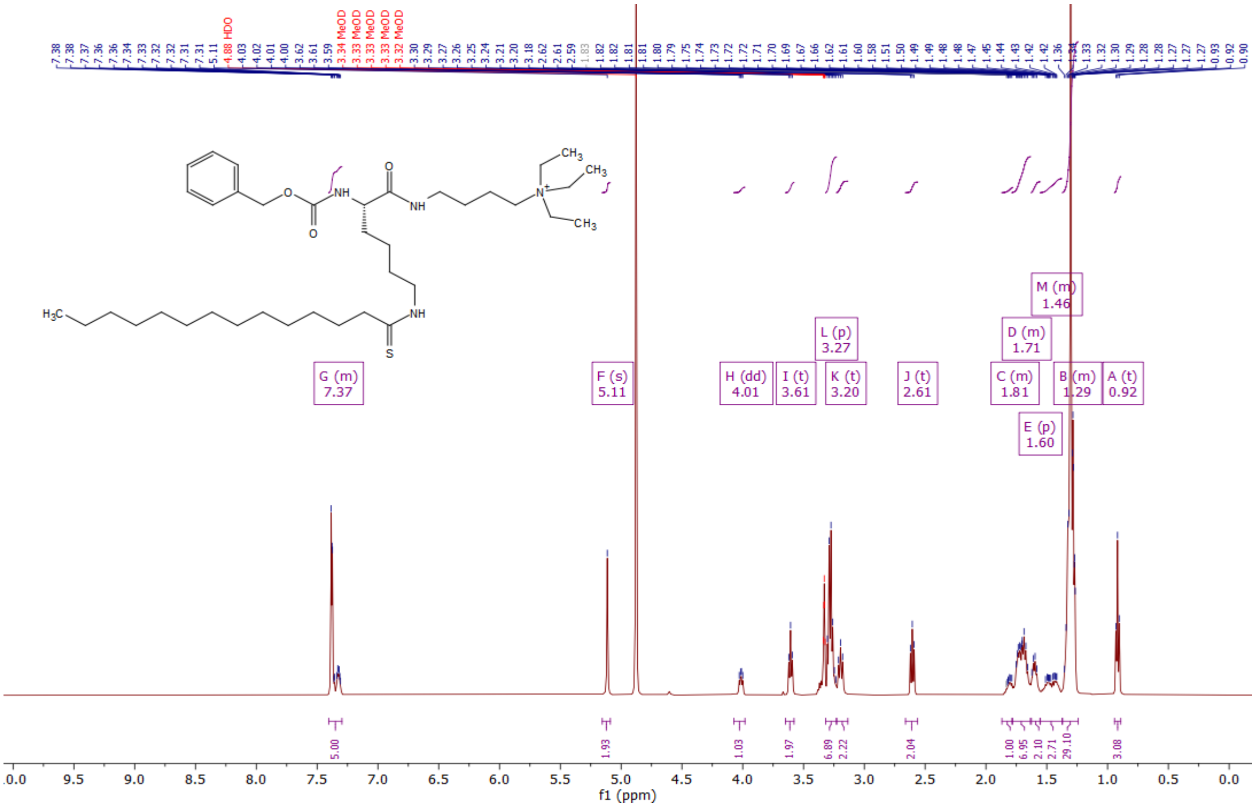

SJ-106C ^{13}C NMR

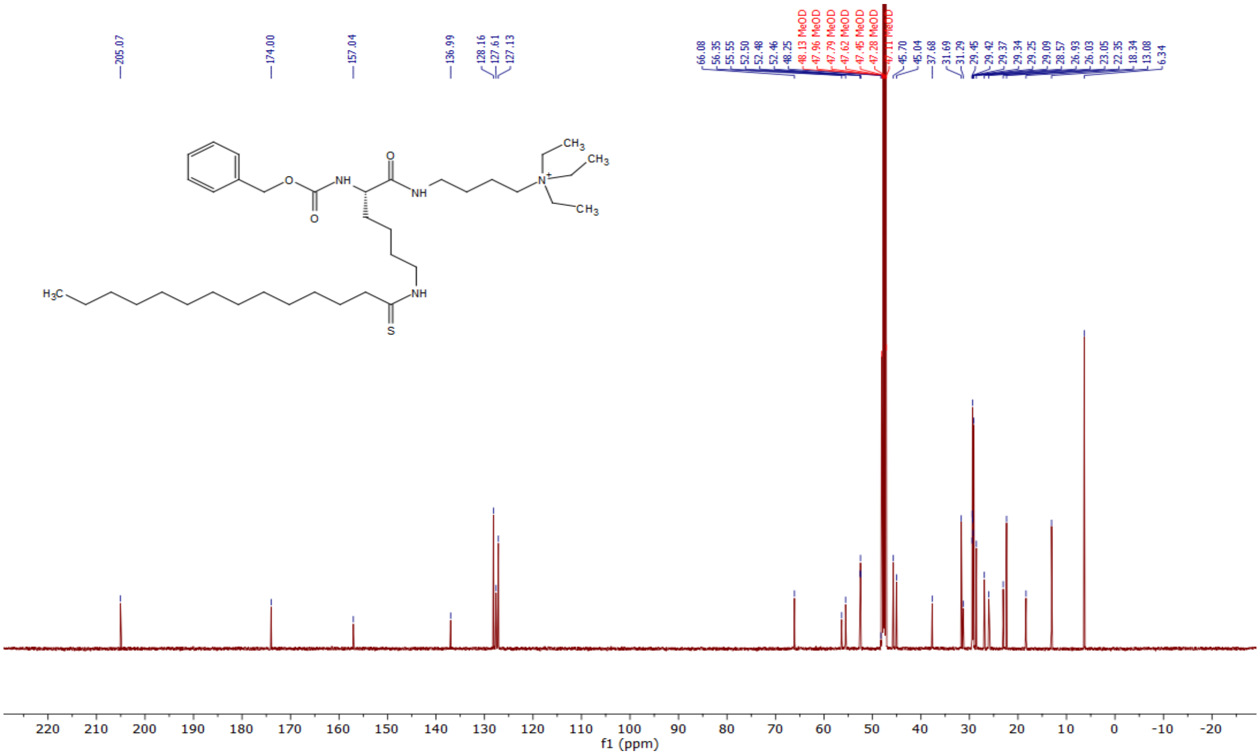

SJ-172 ^{1}H NMR

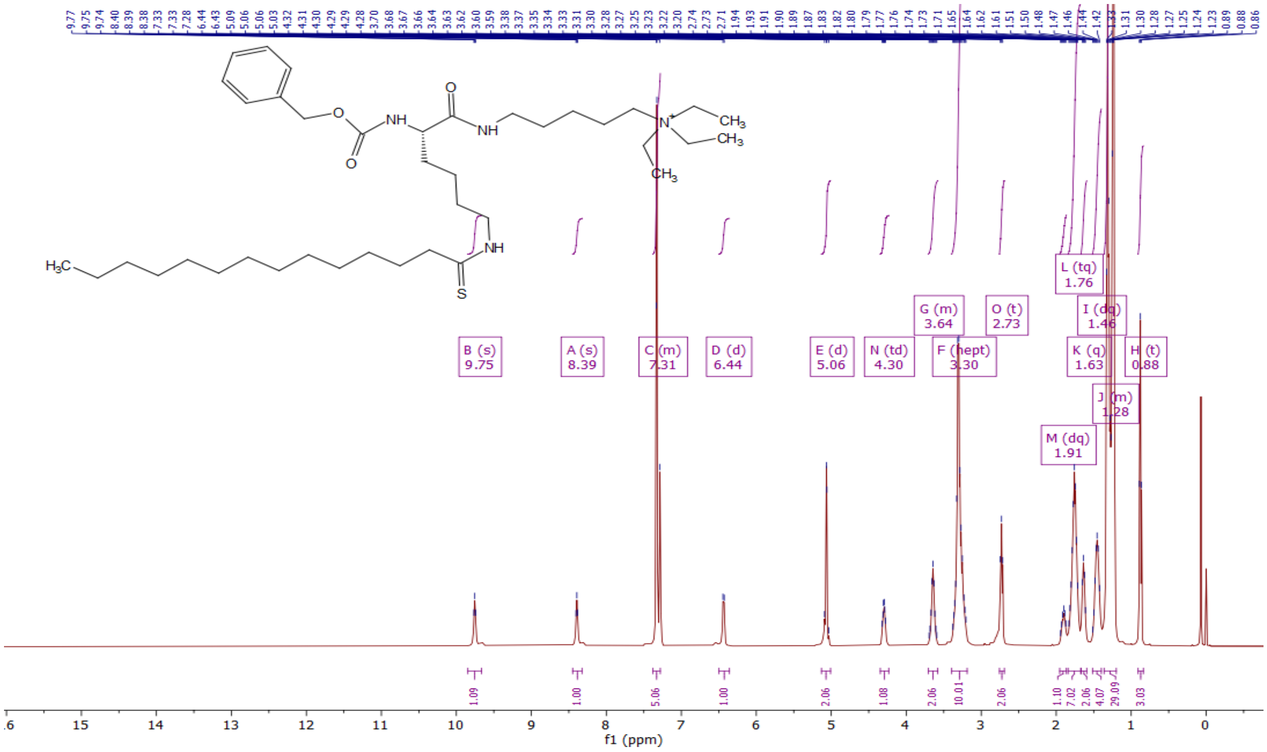

SJ-172 ^{13}C NMR

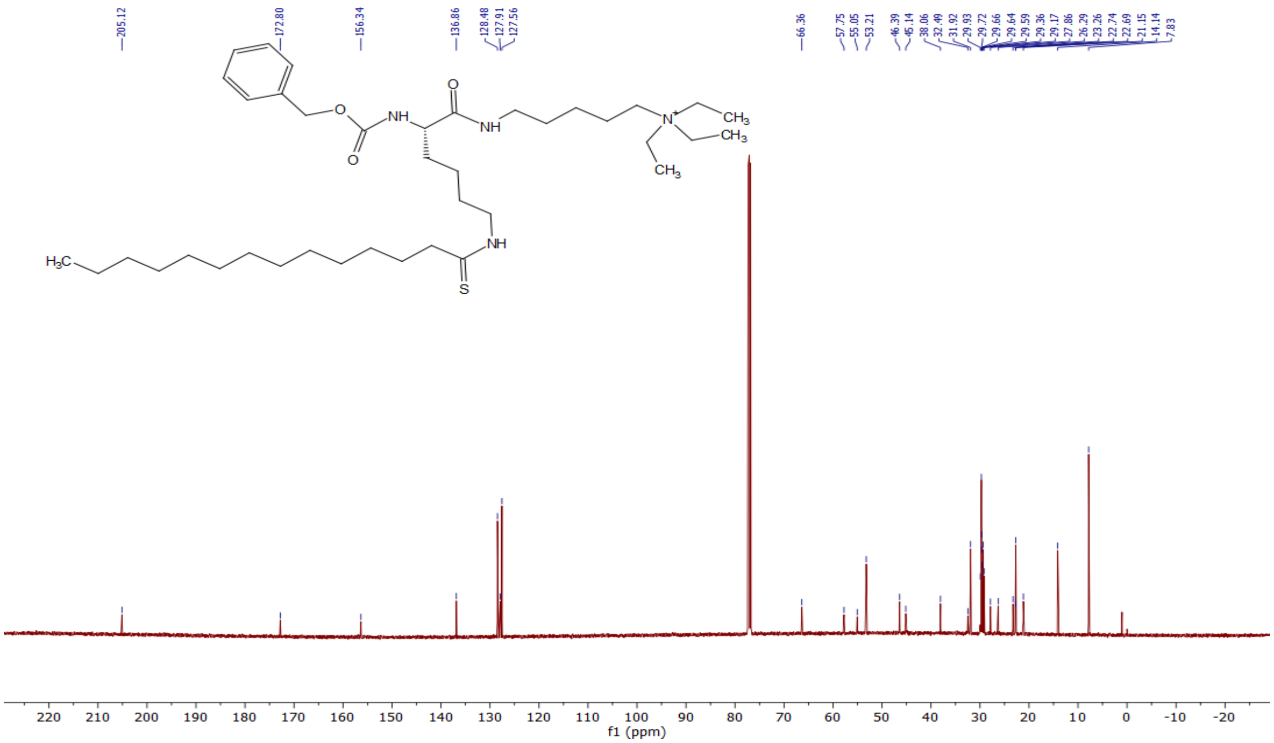

SJ-174 ^{1}H NMR

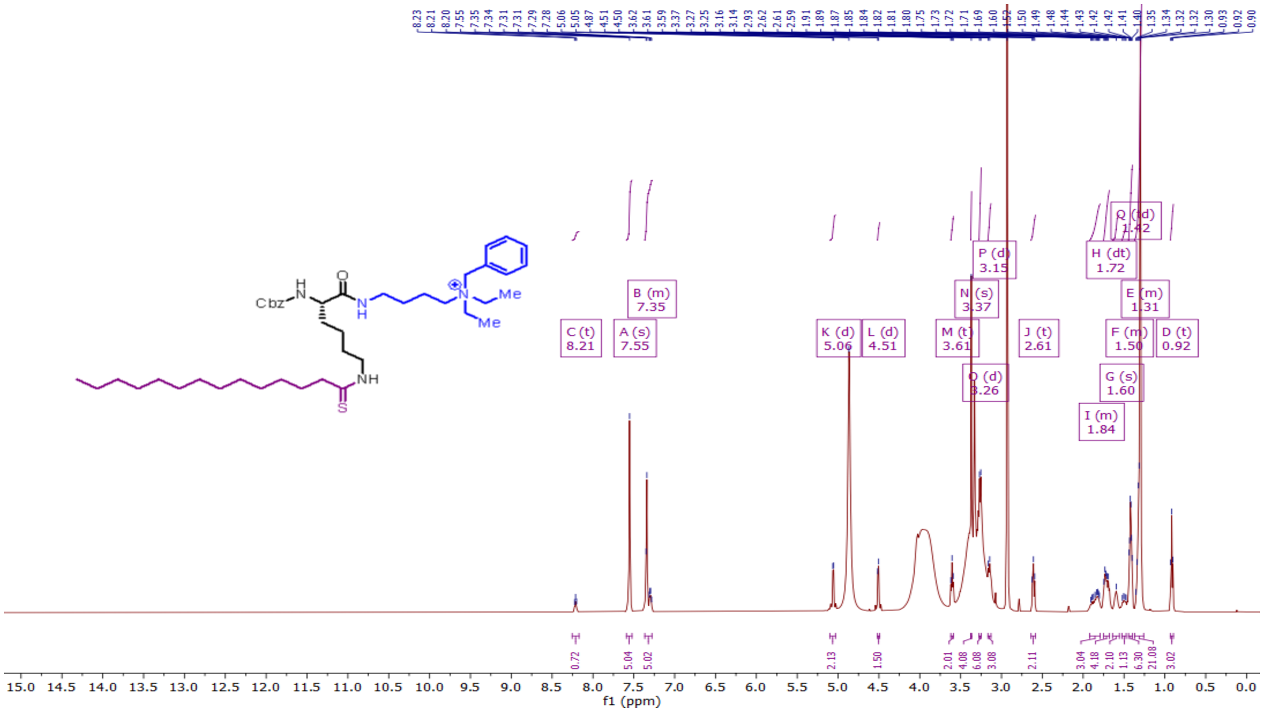

SJ-174 ^{13}C NMR

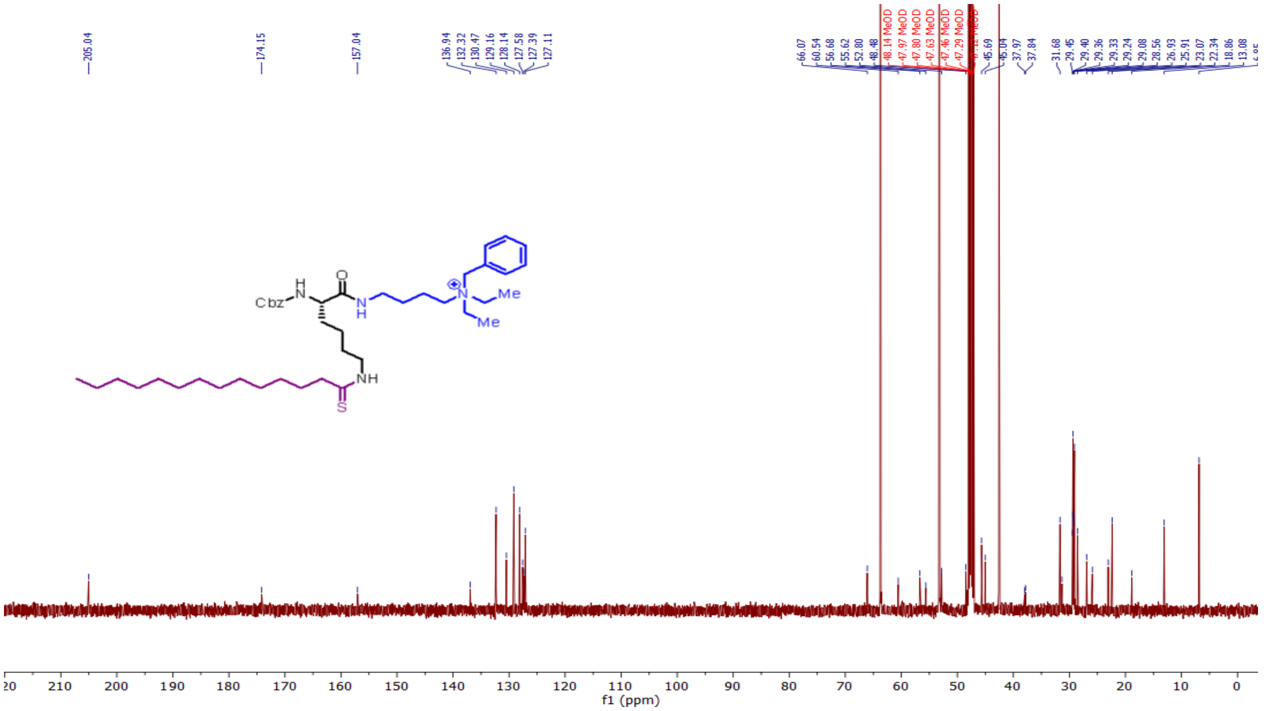

SJ-218 ^{1}H NMR: compound

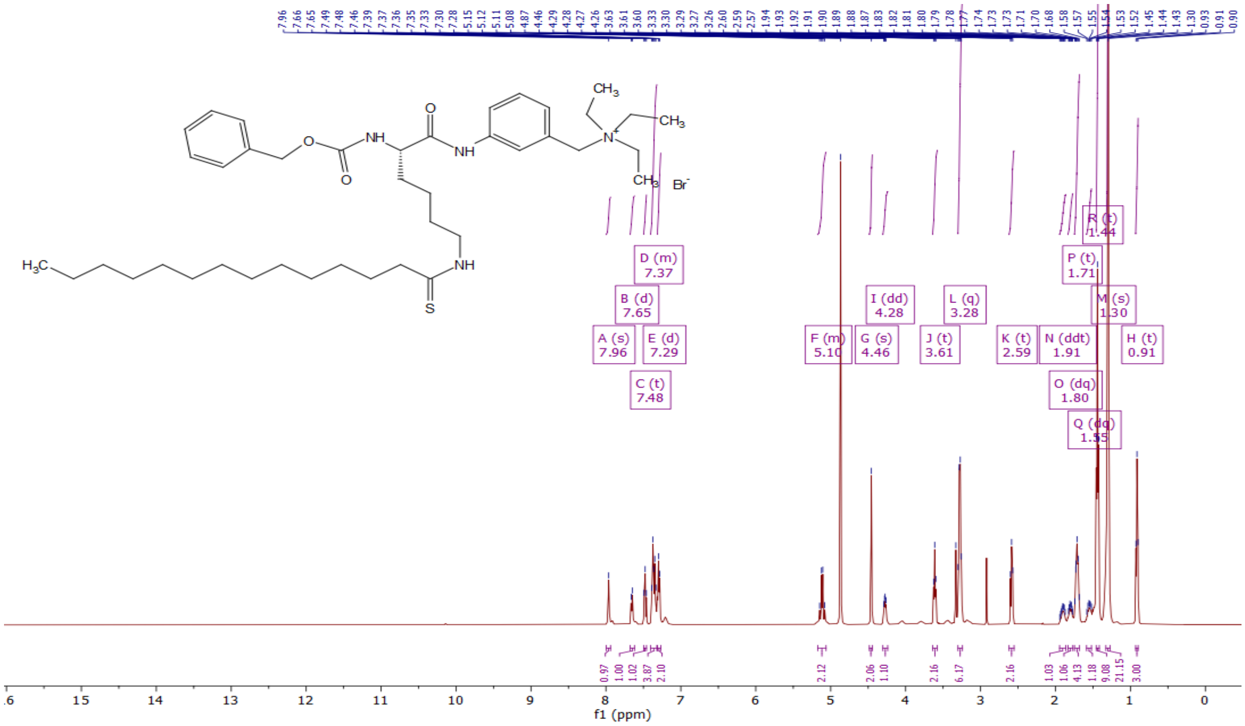

SJ-218 ^{13}C NMR: compound

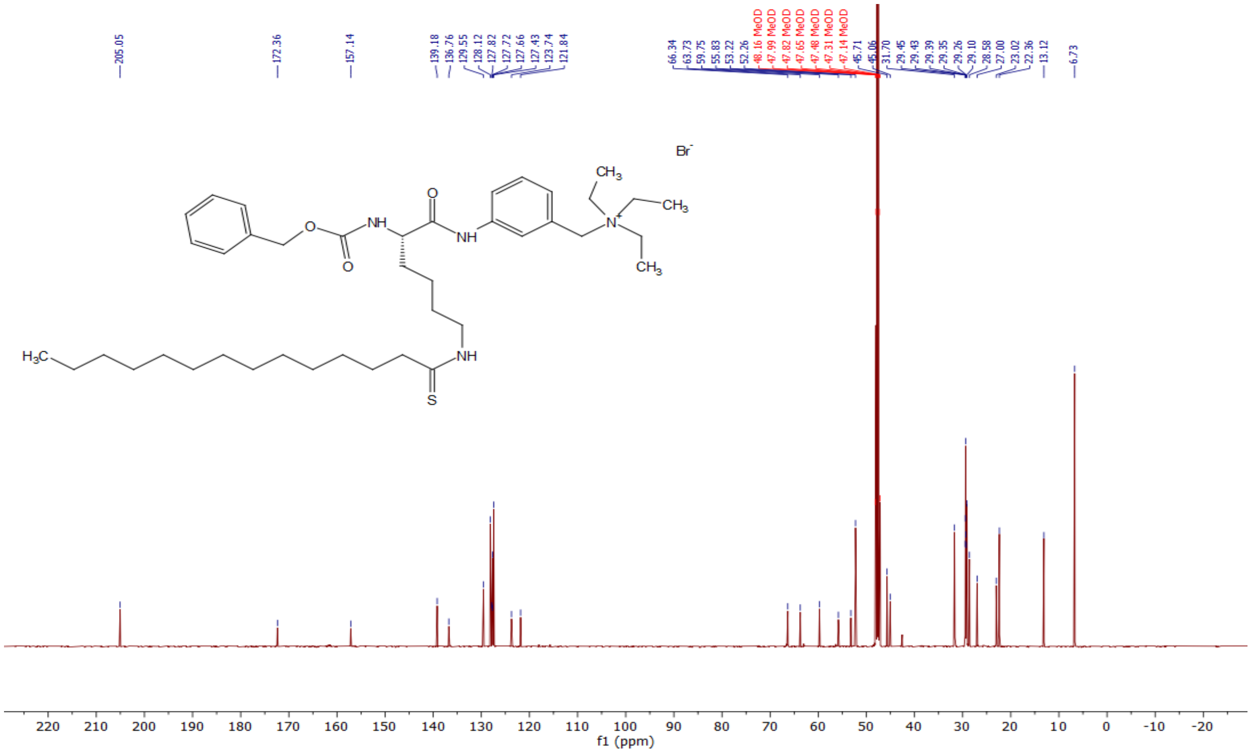

SJ-155 ^{1}H NMR

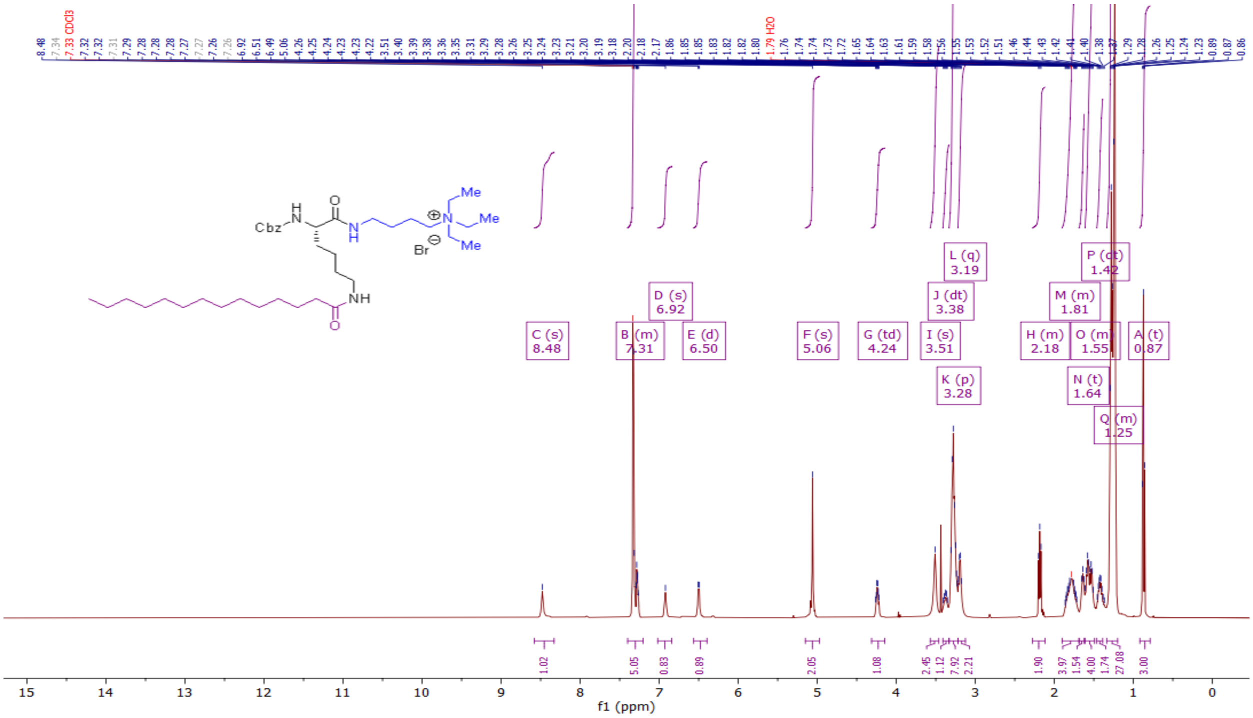

SJ-155 ^{13}C NMR

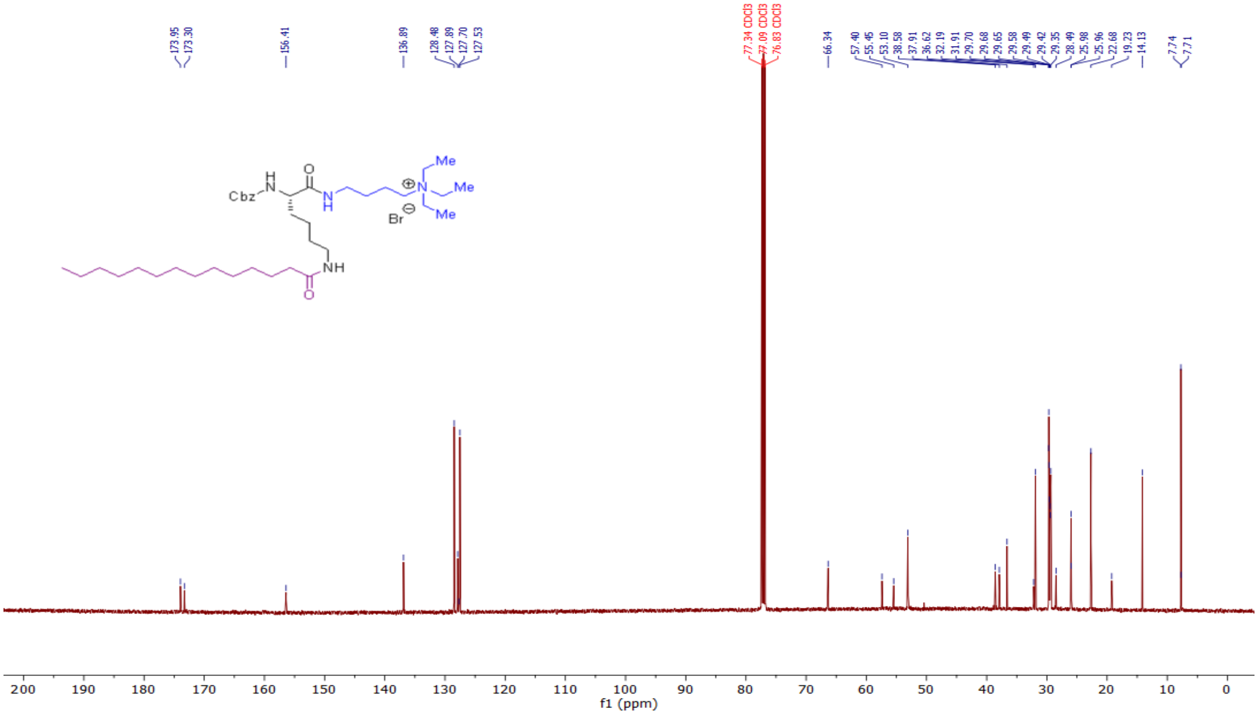

SJ-105 ^{1}H NMR

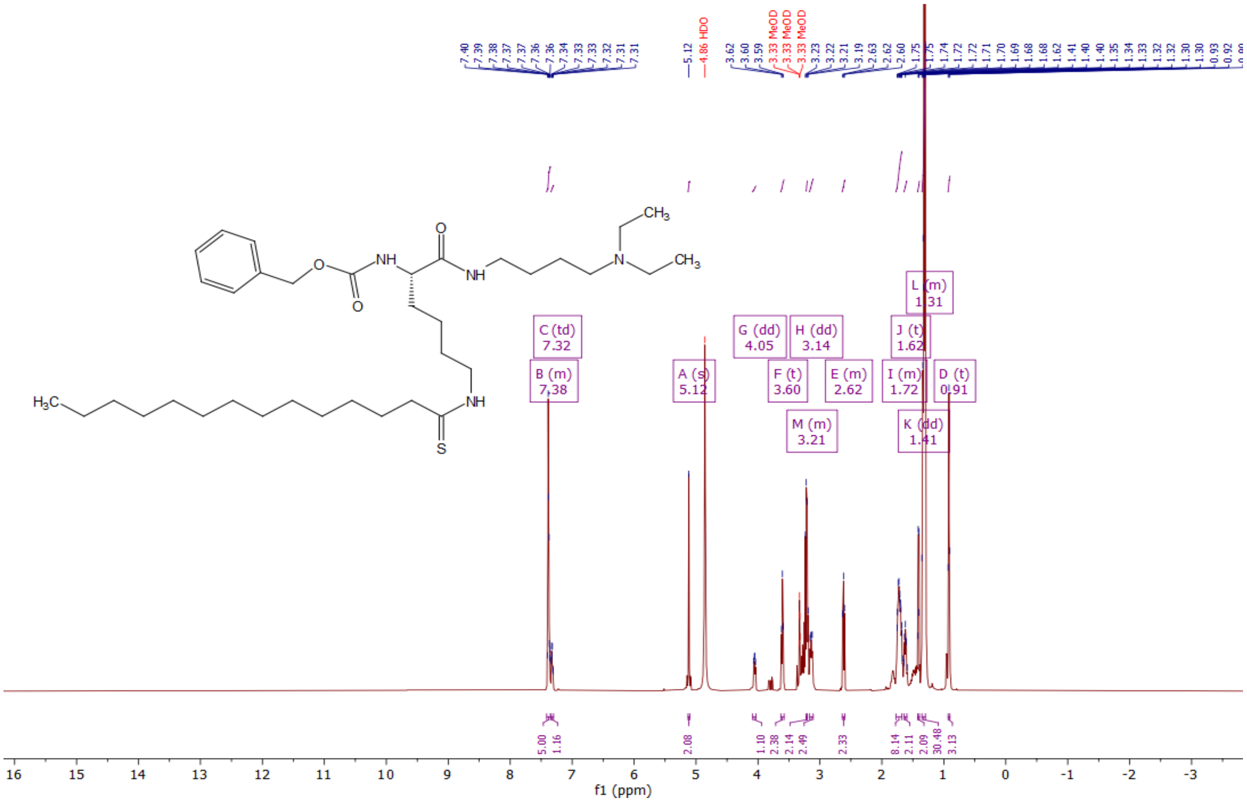

SJ-105 ^{13}C NMR

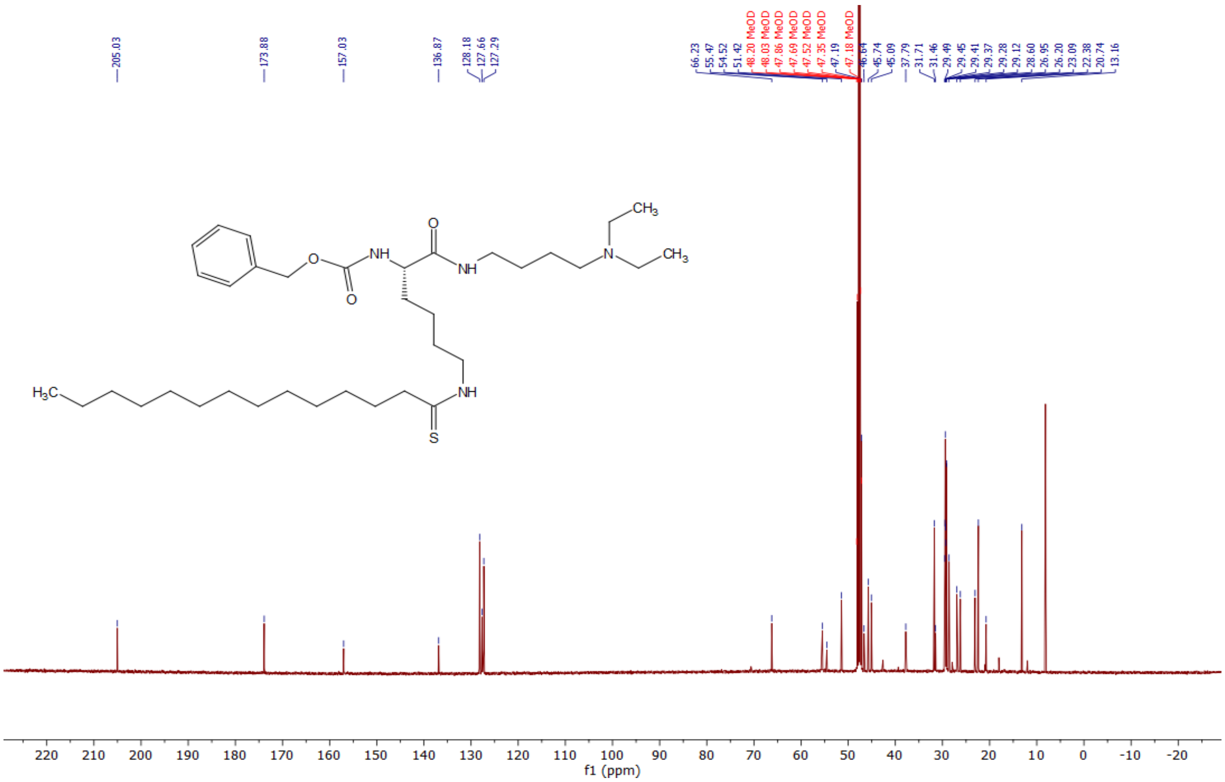

SJ-184 ^{1}H NMR

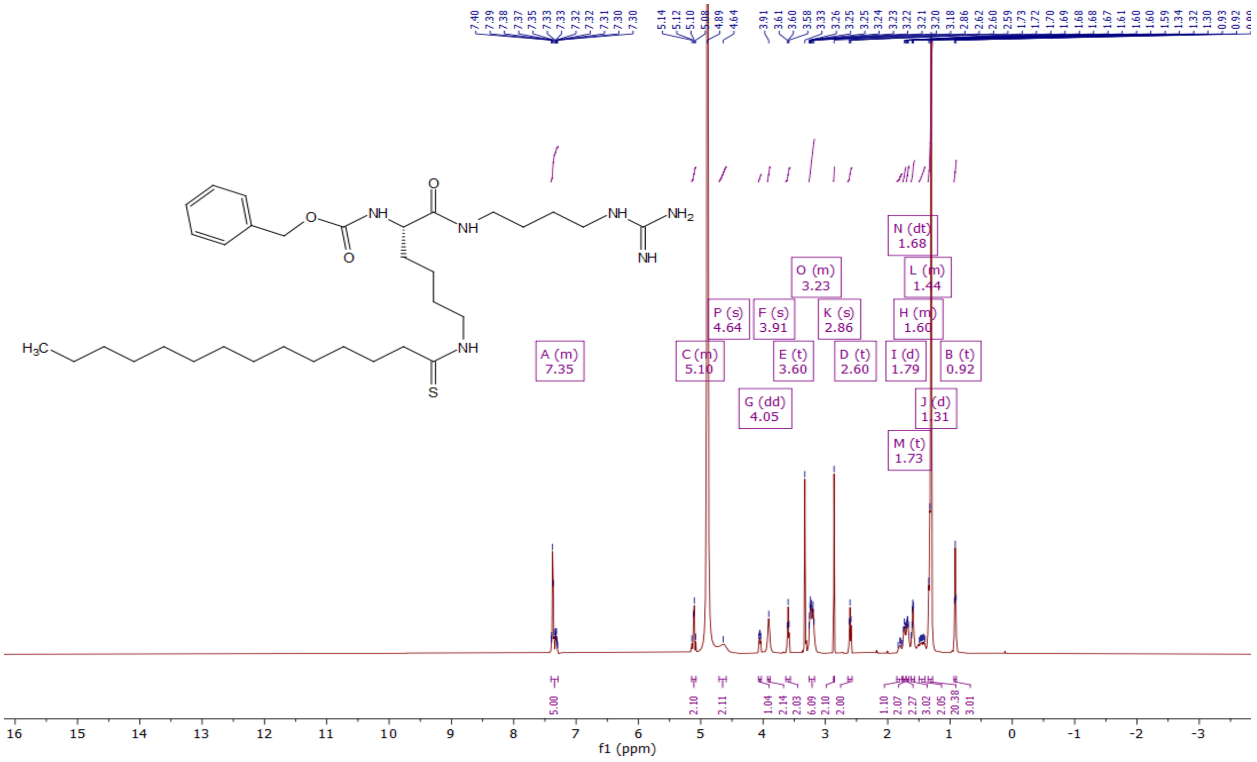

SJ-184 ^{13}C NMR

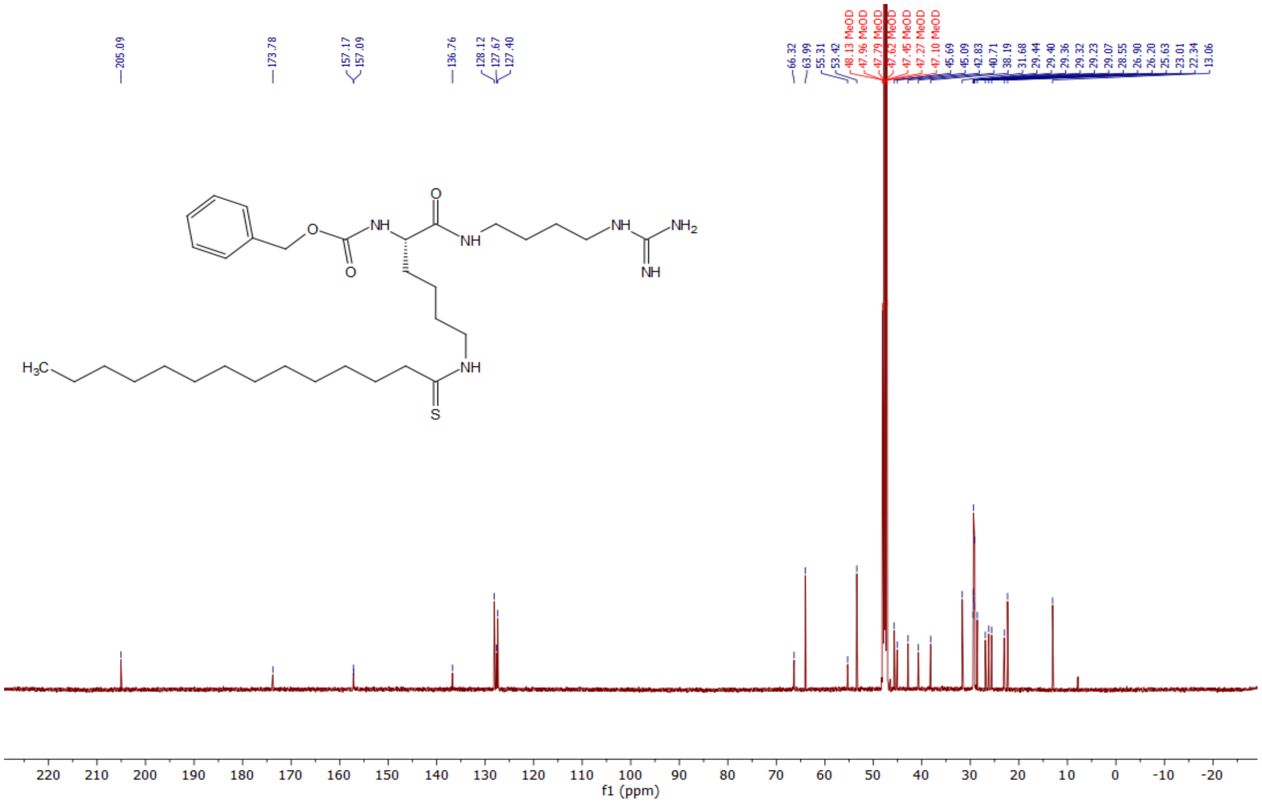

SJ-148 ^{1}H NMR

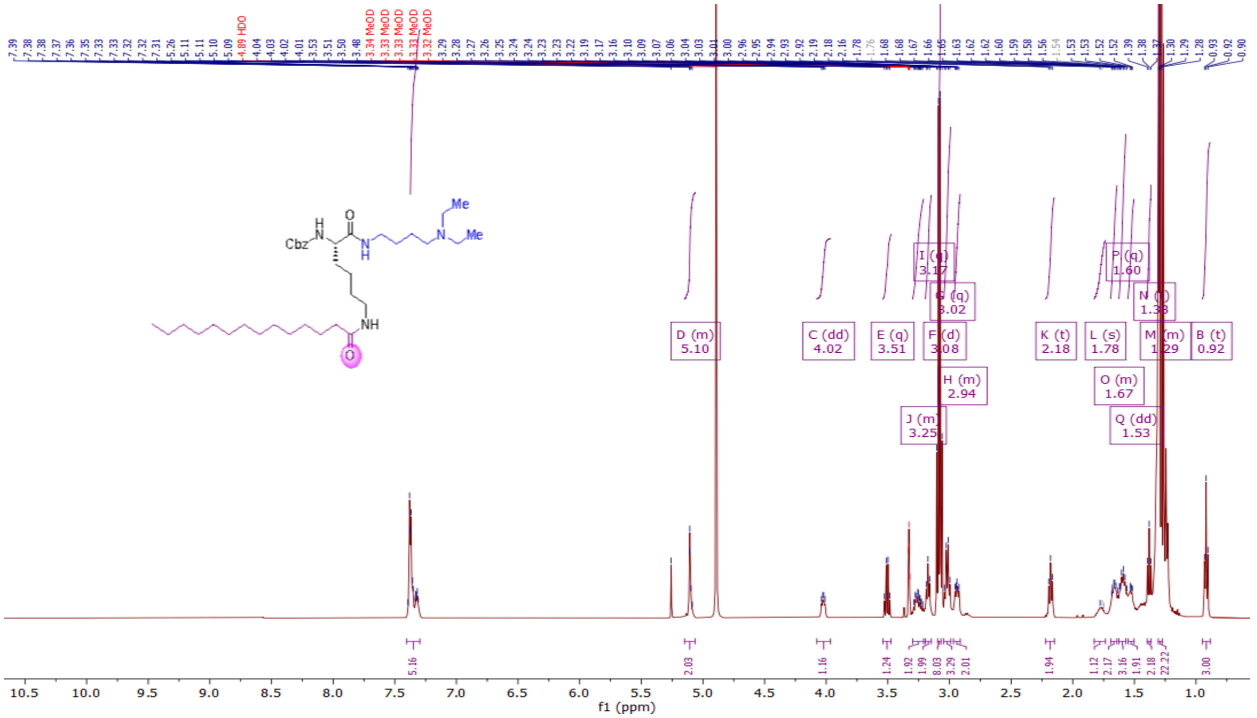

SJ-148 ^{13}C NMR

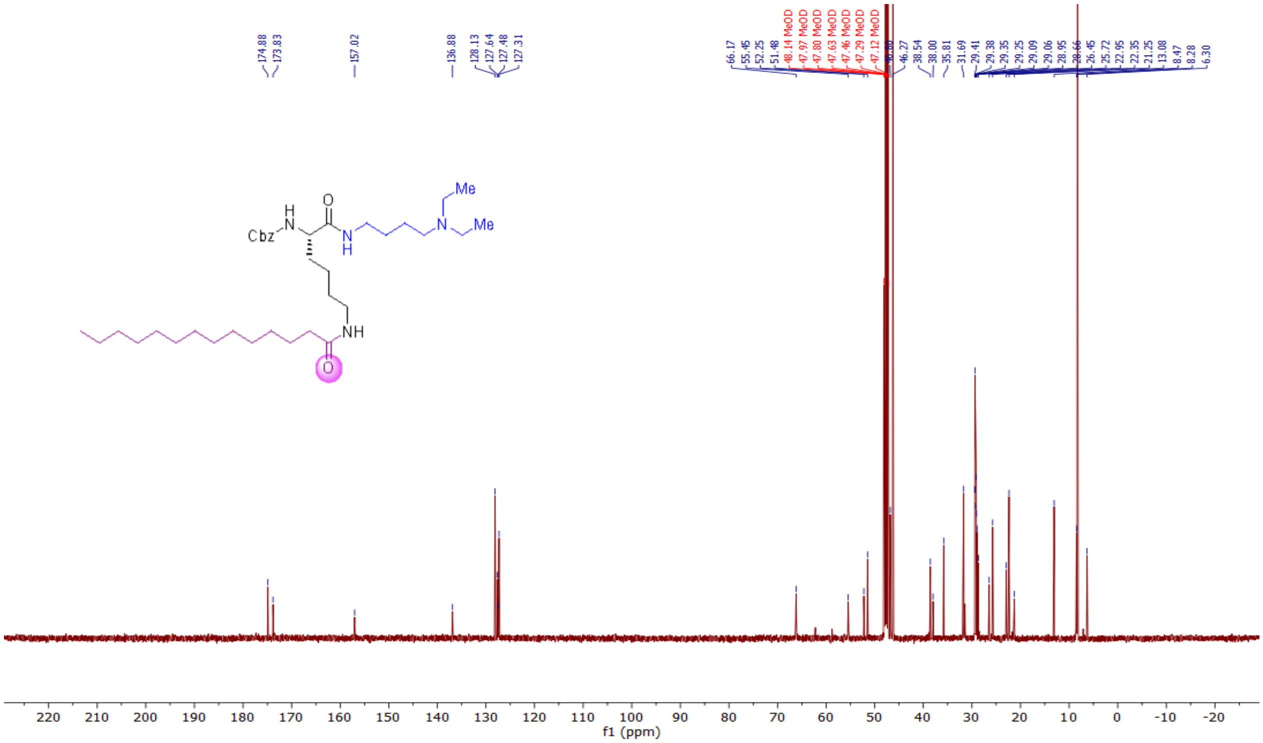

SJ-183 ^{1}H NMR

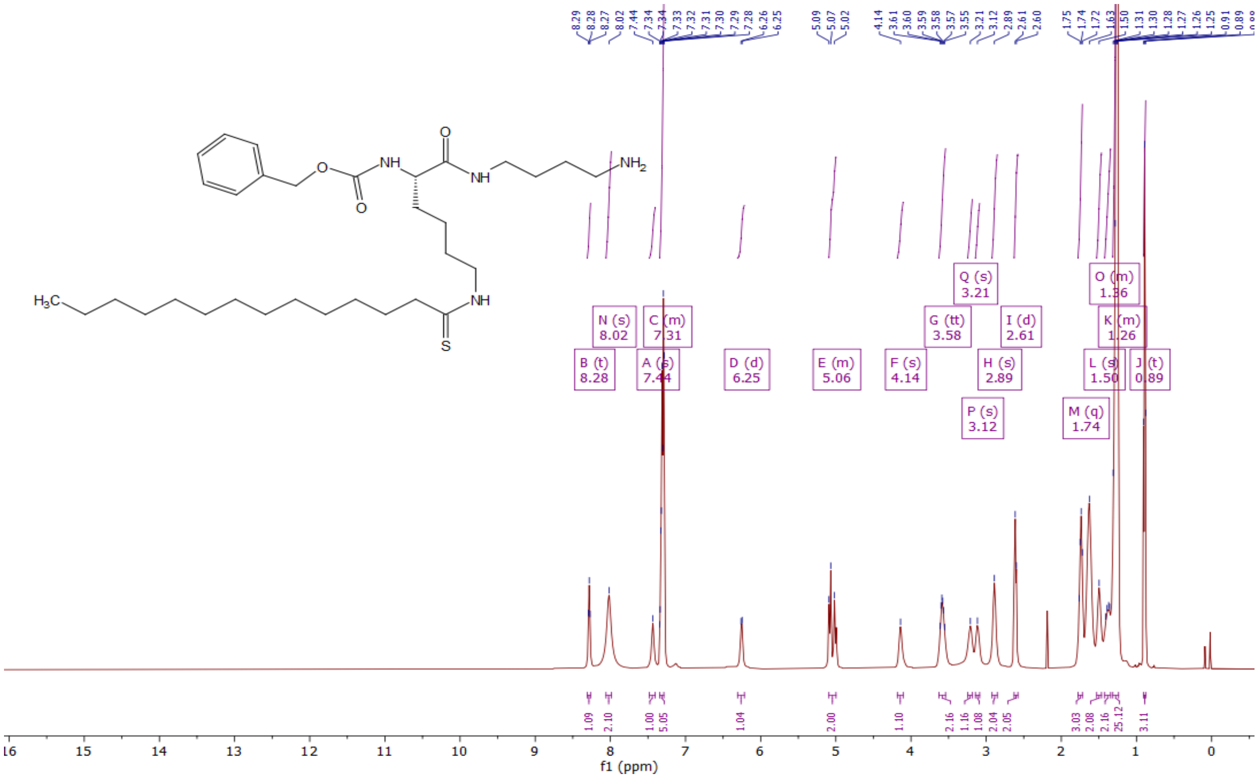

SJ-183 ^{13}C NMR

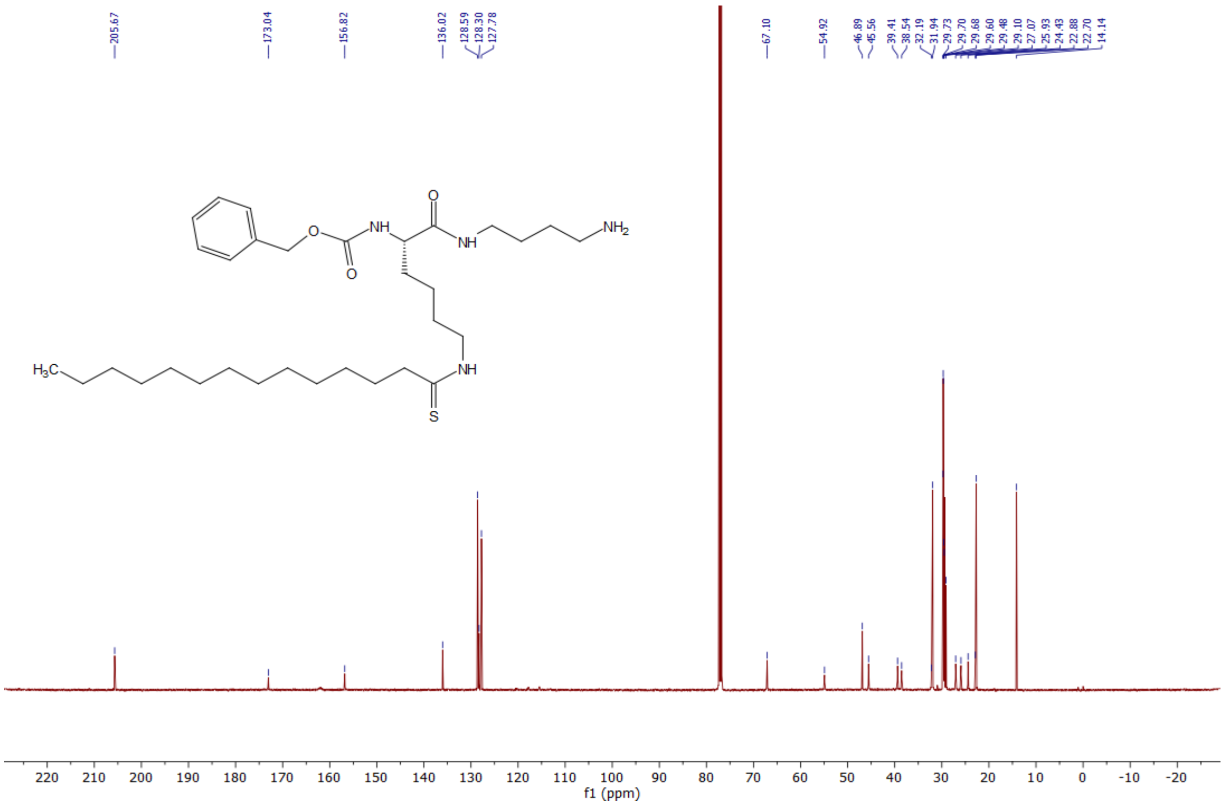

SJ-200 ^{1}H NMR

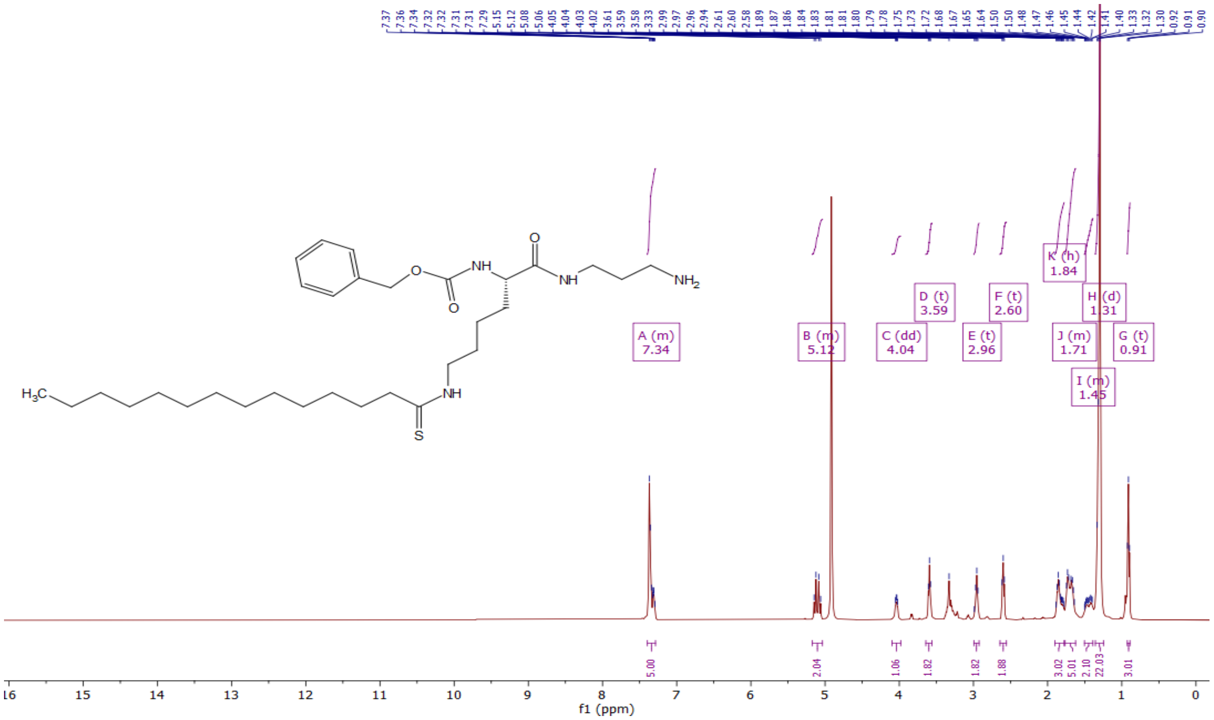

SJ-200 ^{13}C NMR

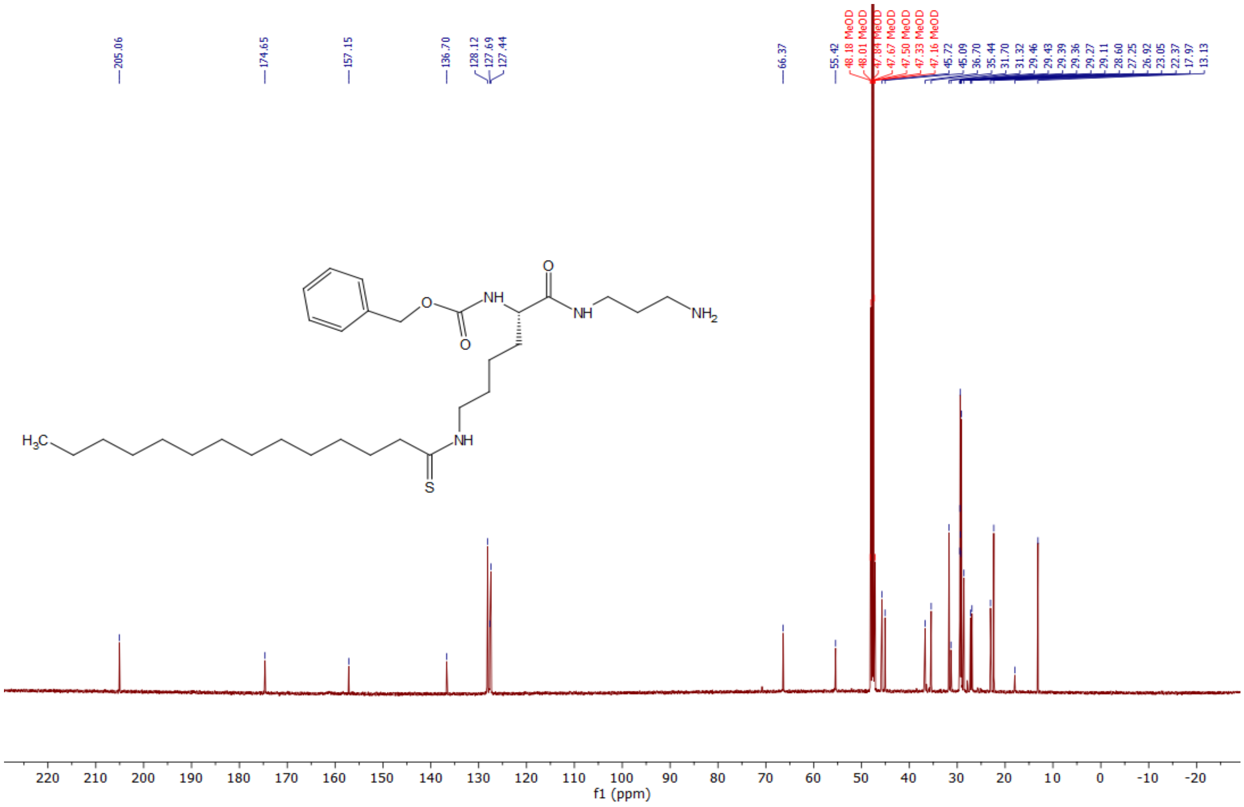

SJ-207R ^{1}H NMR

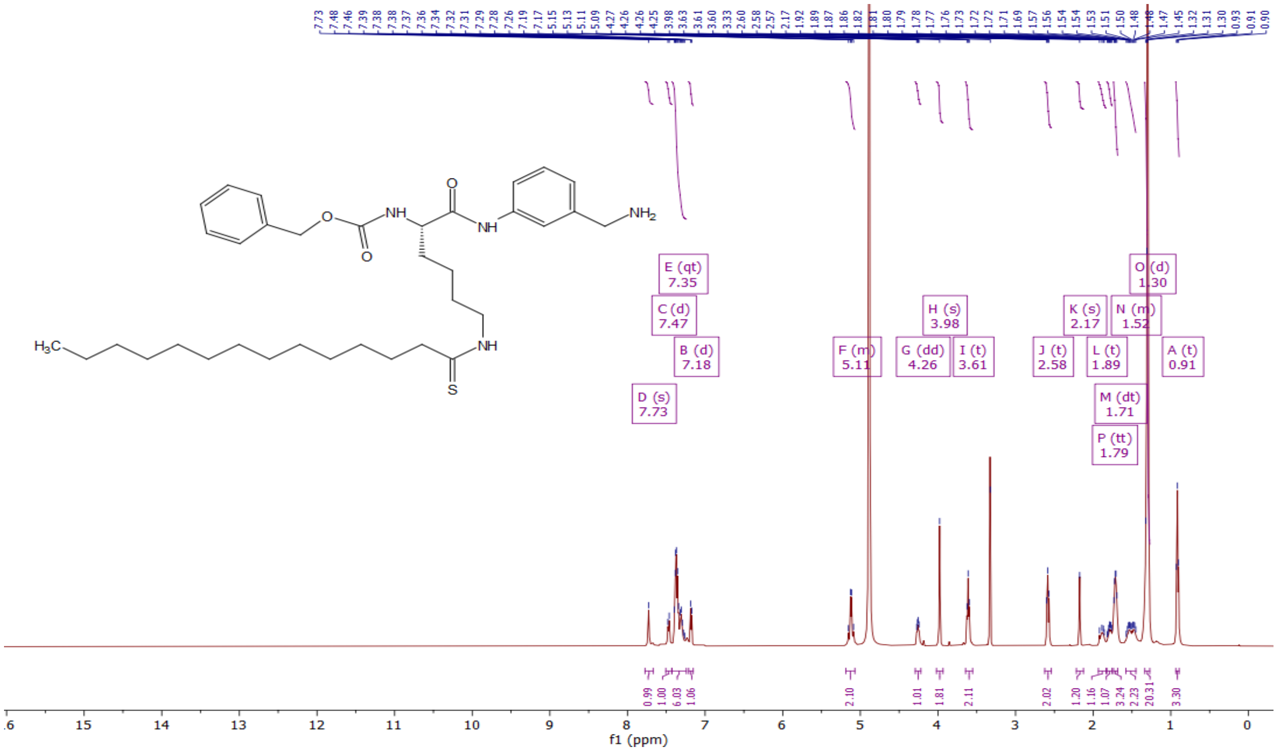

SJ-207R ^{13}C NMR

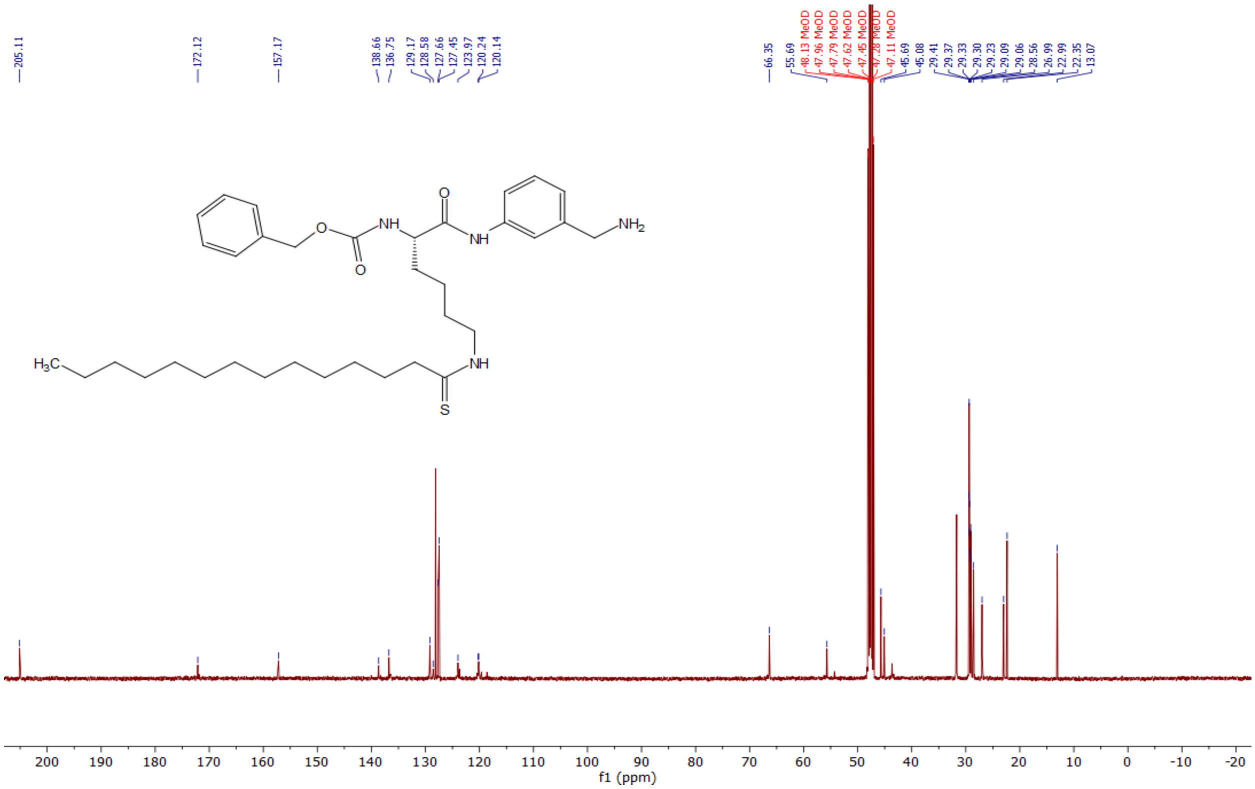

SJ-202 ^{1}H NMR

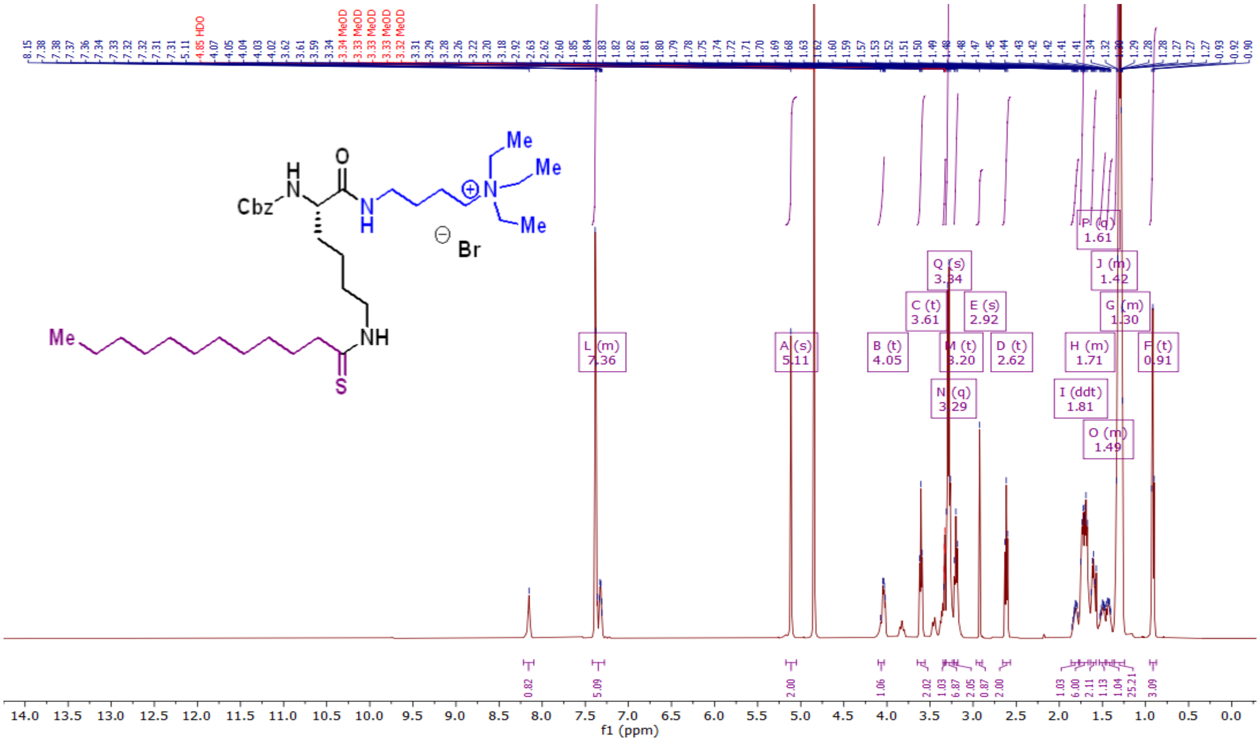

SJ-202 ^{13}C NMR

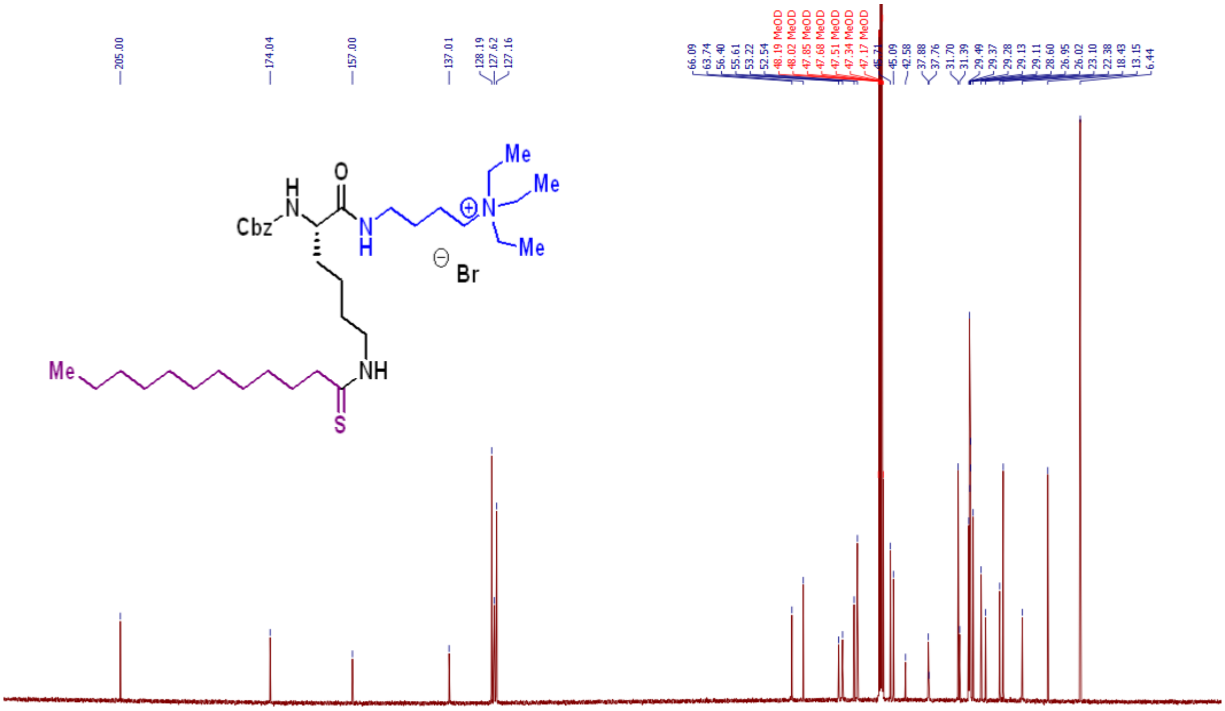

SJ-204 ^{1}H NMR

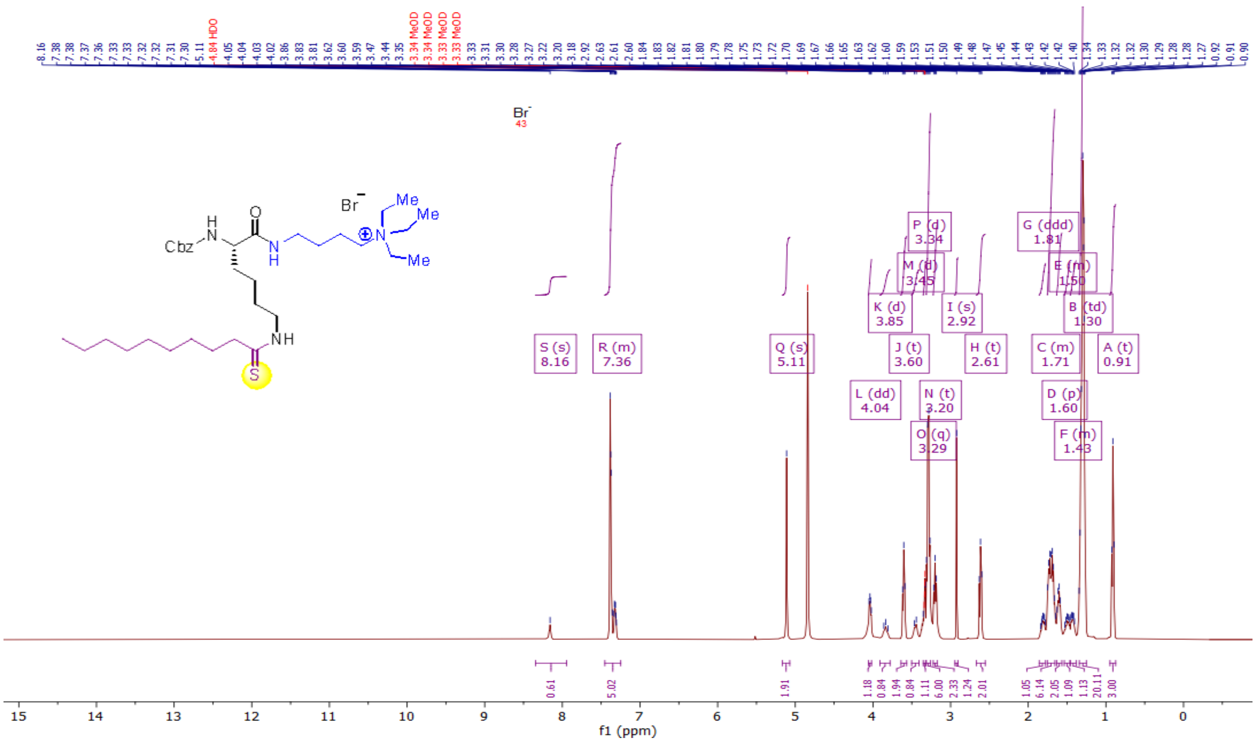

SJ-204 ^{13}C NMR

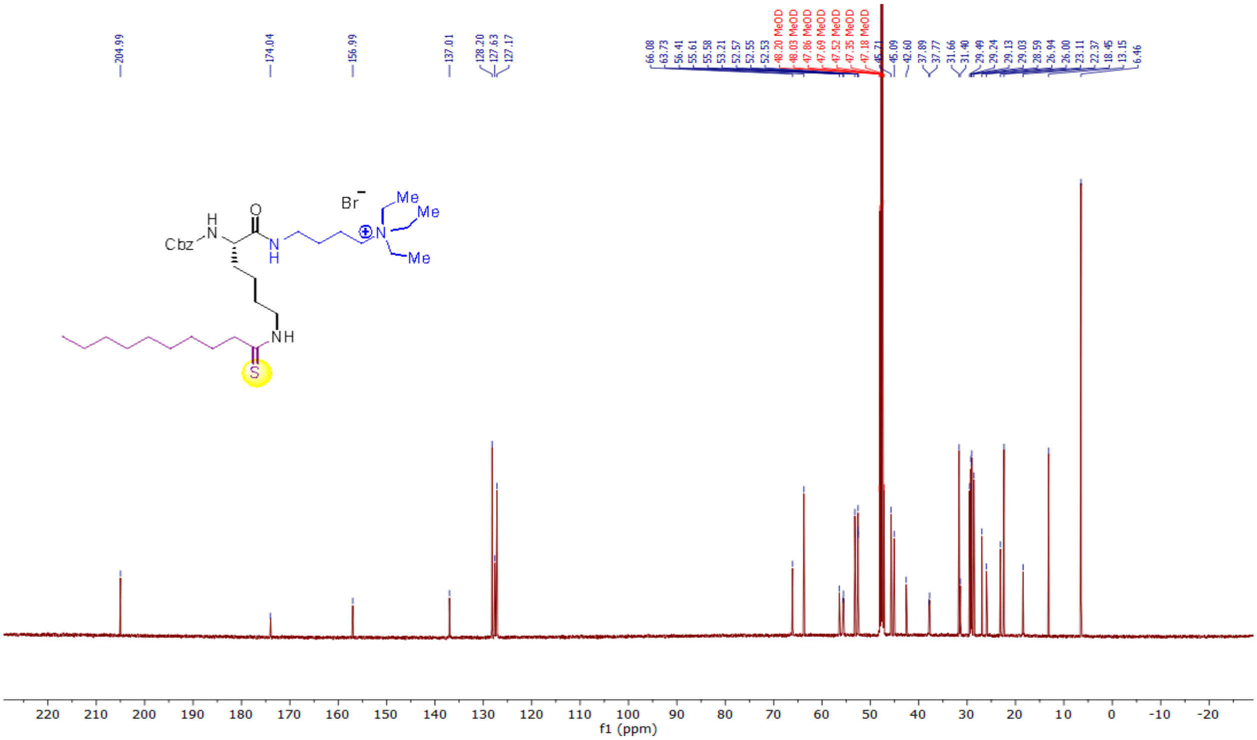

SJ-206 ^{1}H NMR

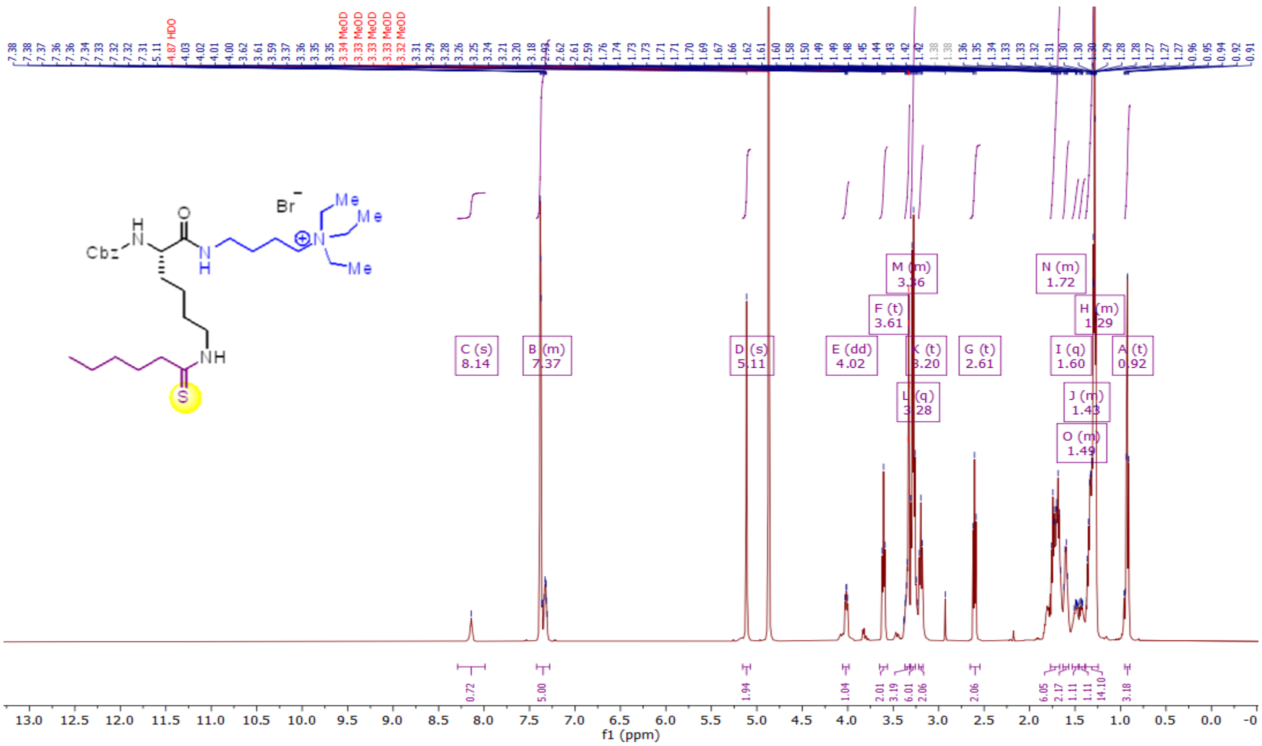

SJ-206 ^{13}C NMR

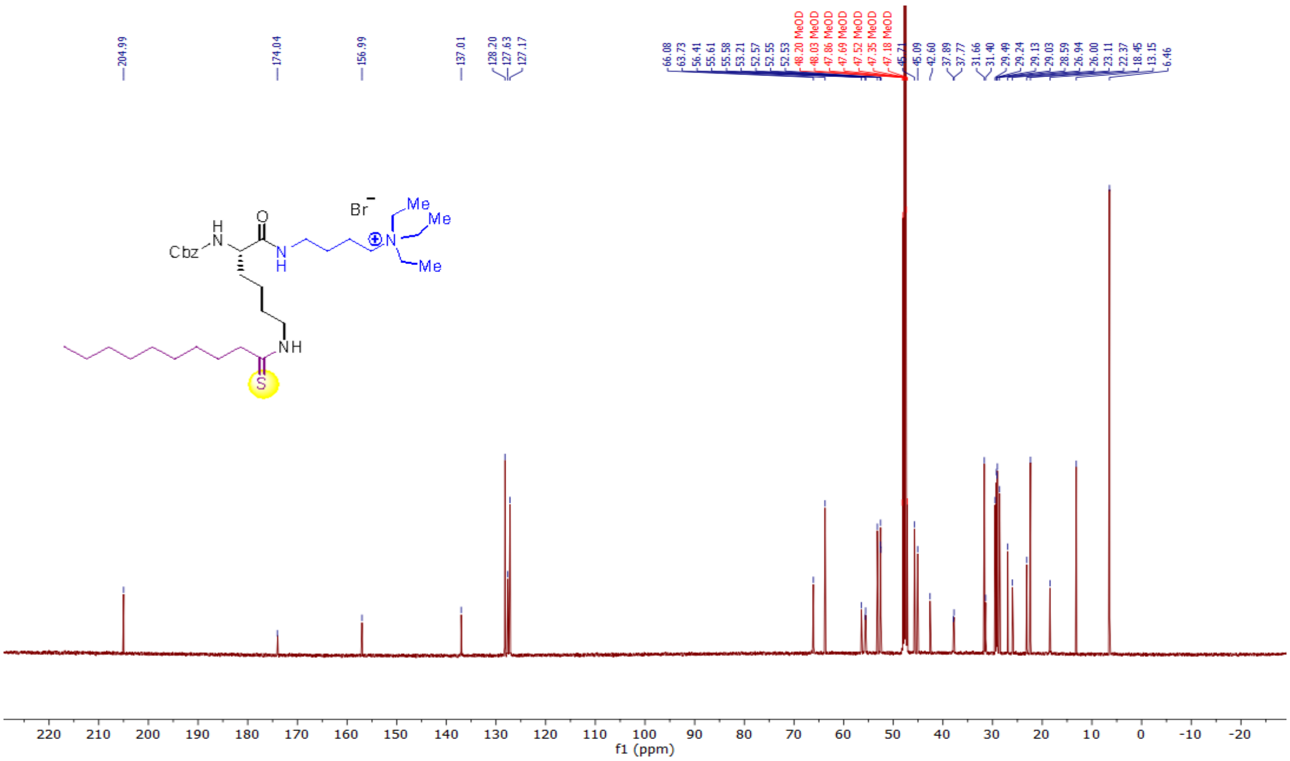

SJ-213 ^{1}H NMR

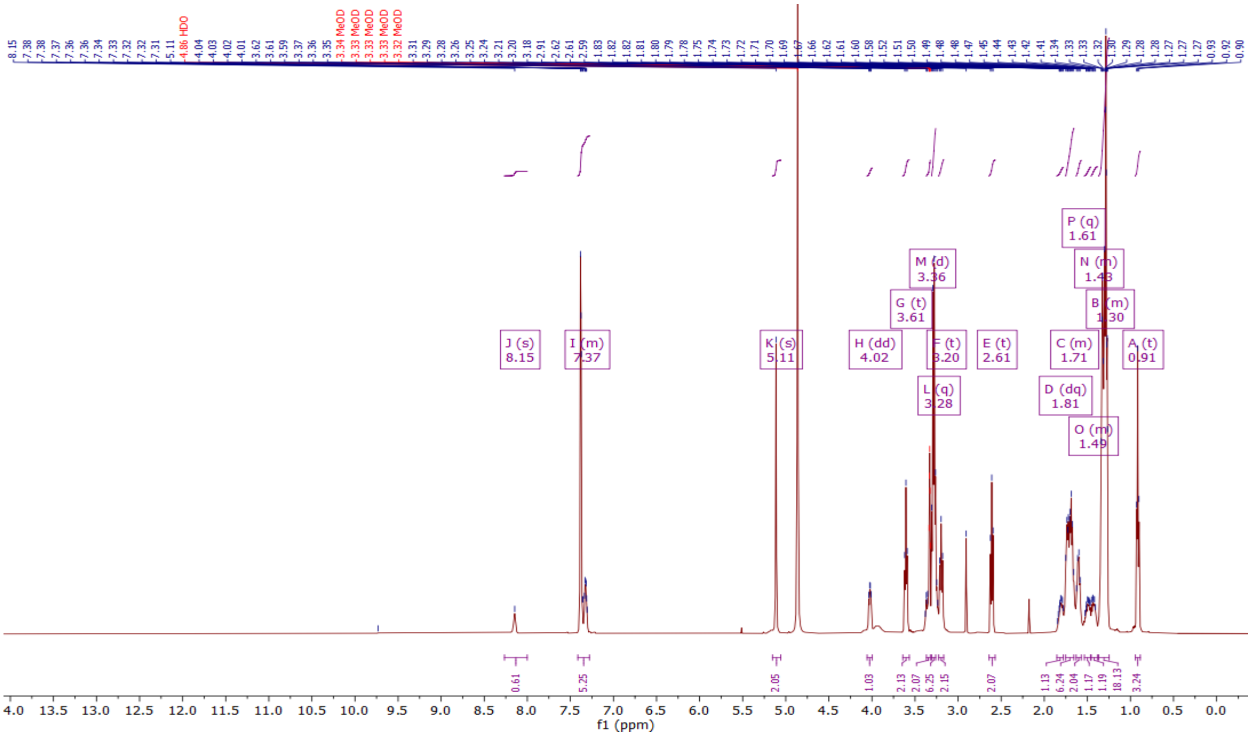

SJ-213 ^{13}C NMR

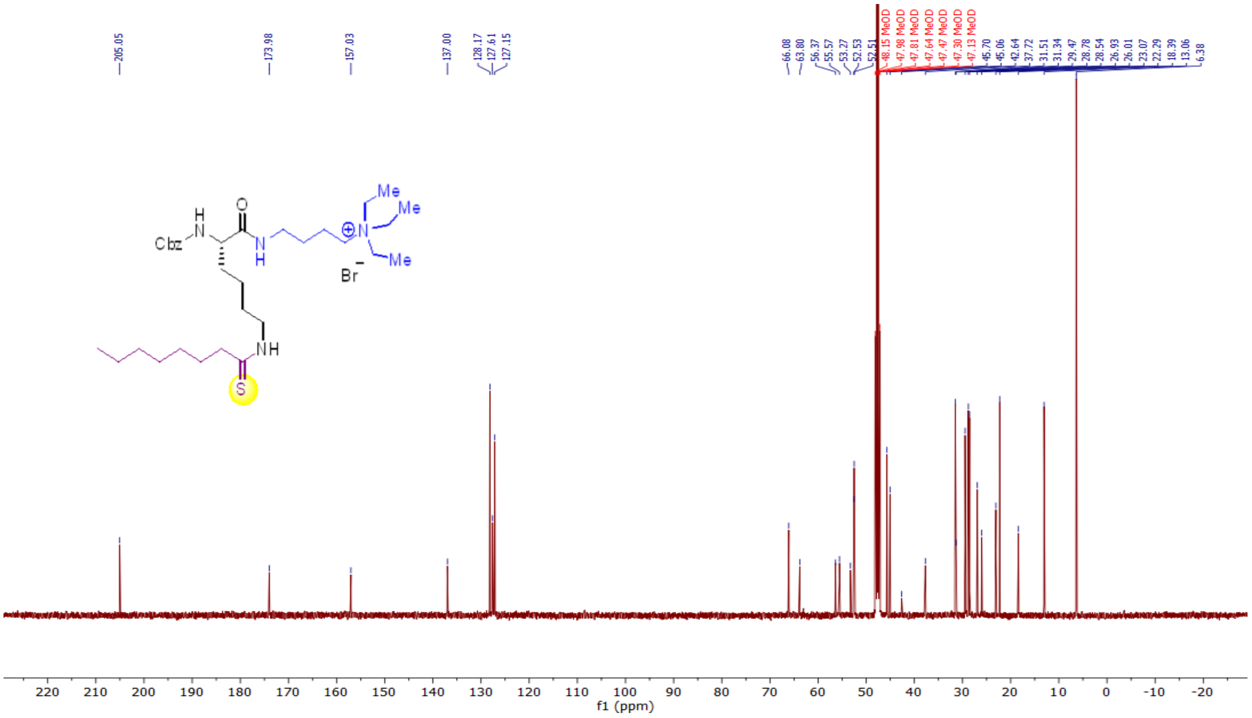

SJ-112A ^{1}H NMR

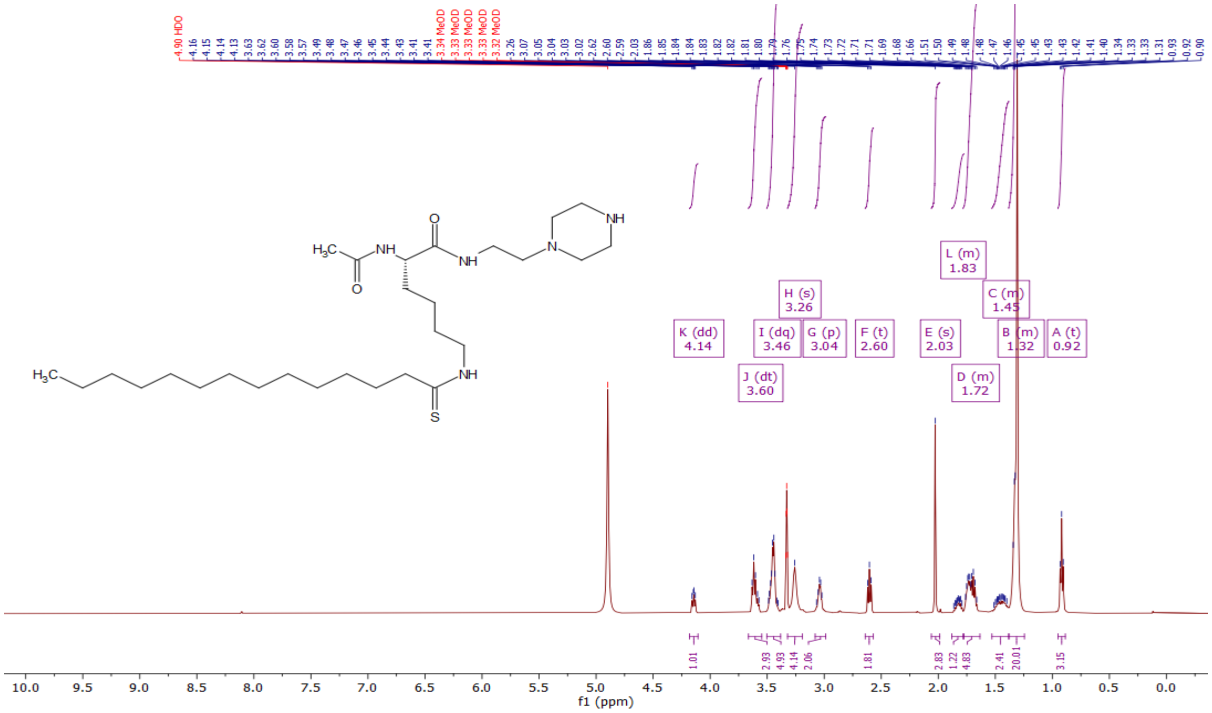

SJ-112A ^{13}C NMR

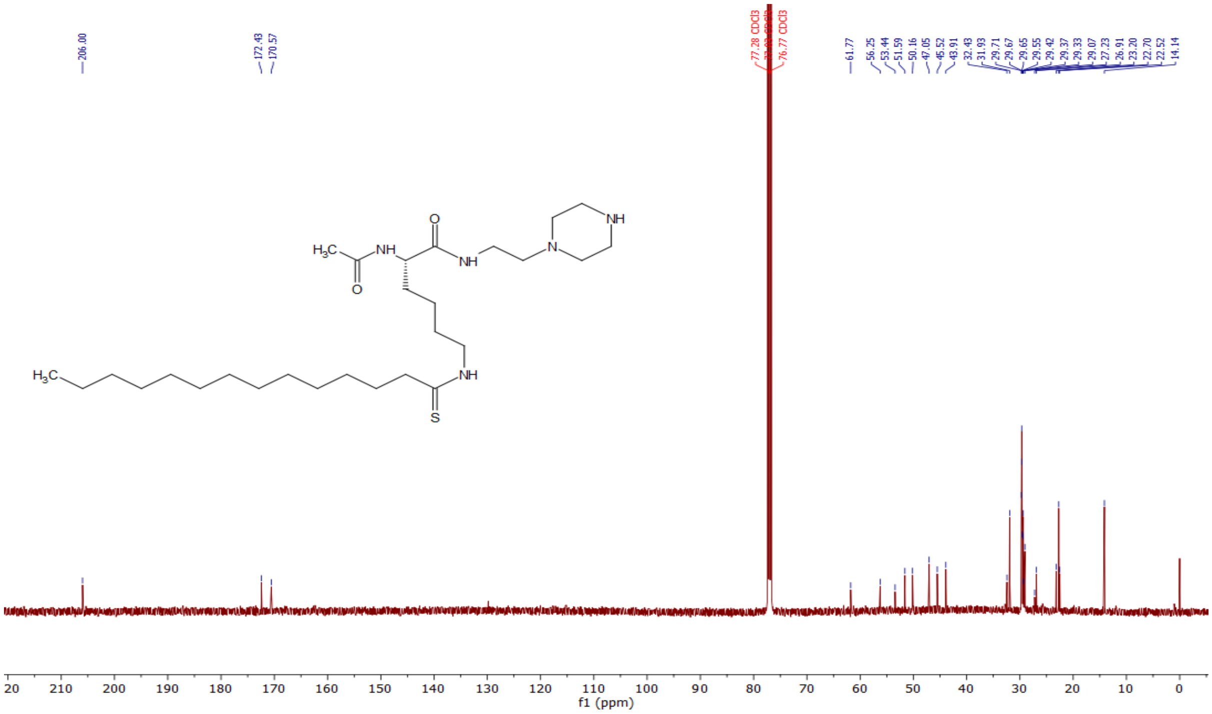

HPLC purity determination for SJ-106C

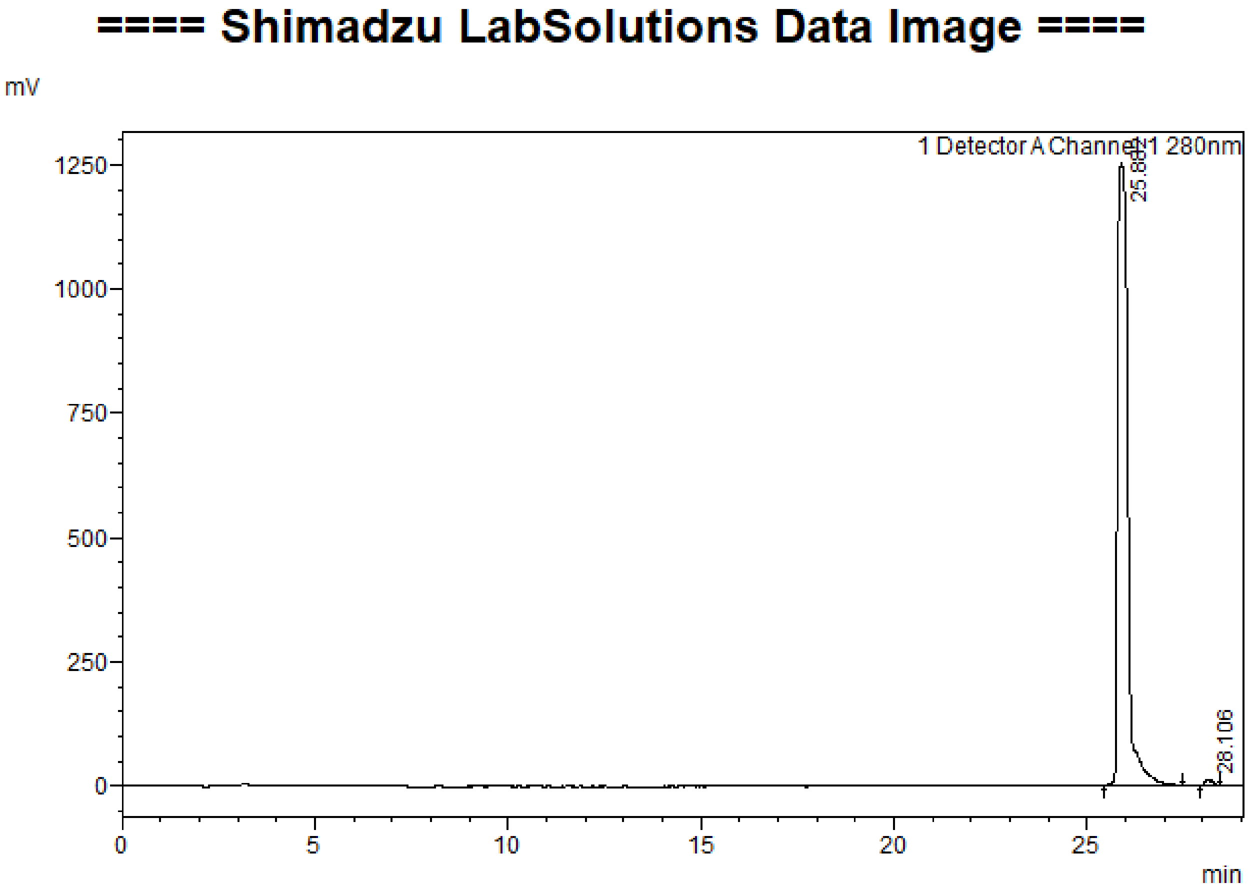

Peak	Ret. time	Area	Height	Area%
1	25.882	23507230	1249329	99.318
2	28.106	161478	13402	0.682
Total		23668708	1262731	100
